# Characterisation of the Broadly-Specific *O*-Methyl-transferase JerF from the Late Stages of Jerangolid Biosynthesis

**DOI:** 10.3390/molecules21111443

**Published:** 2016-10-29

**Authors:** Steffen Friedrich, Franziska Hemmerling, Frederick Lindner, Anna Warnke, Johannes Wunderlich, Gesche Berkhan, Frank Hahn

**Affiliations:** 1 Zentrum für Biomolekulare Wirkstoffe, Leibniz-Universität Hannover, Schneiderberg 38, 30167 Hannover, Germany; steffen.friedrich@oci.uni-hannover.de (S.F.); franziska.hemmerling@oci.uni-hannover.de (F.H.); anna.warnke@oci.uni-hannover.de (A.W.); gesche.berkhan@oci.uni-hannover.de (G.B.); 2 Professur für Organische Chemie (Lebensmittelchemie), Fakultät für Biologie, Chemie und Geowissenschaften, Universitätsstraße 30, 95447 Bayreuth, Germany; frederick.lindner@uni-bayreuth.de (F.L.); johannes.wunderlich1@uni-bayreuth.de (J.W.)

**Keywords:** methyltransferases, enzymes, chemoenzymatic synthesis, natural products, chemoselectivity, methylenolethers

## Abstract

We describe the characterisation of the *O*-methyltransferase JerF from the late stages of jerangolid biosynthesis. JerF is the first known example of an enzyme that catalyses the formation of a non-aromatic, cyclic methylenolether. The enzyme was overexpressed in *E. coli* and the cell-free extracts were used in bioconversion experiments. Chemical synthesis gave access to a series of substrate surrogates that covered a broad structural space. Enzymatic assays revealed a broad substrate tolerance and high regioselectivity of JerF, which makes it an attractive candidate for an application in chemoenzymatic synthesis with particular usefulness for late stage application on 4-methoxy-5,6-dihydro-2*H*-pyran-2-one-containing natural products.

## 1. Introduction

α-Pyrones and γ-pyrones are abundant structures in microbial natural products. Prominent examples for biologically active α-pyrones are coumarin and isocoumarin derivatives, which are extensively used as fragrances, anticoagulants or rodenticides [1]. More recently, 4-hydroxyl-α-pyrones have also been identified as signalling molecules in bacterial communication [2].

**Scheme 1:**
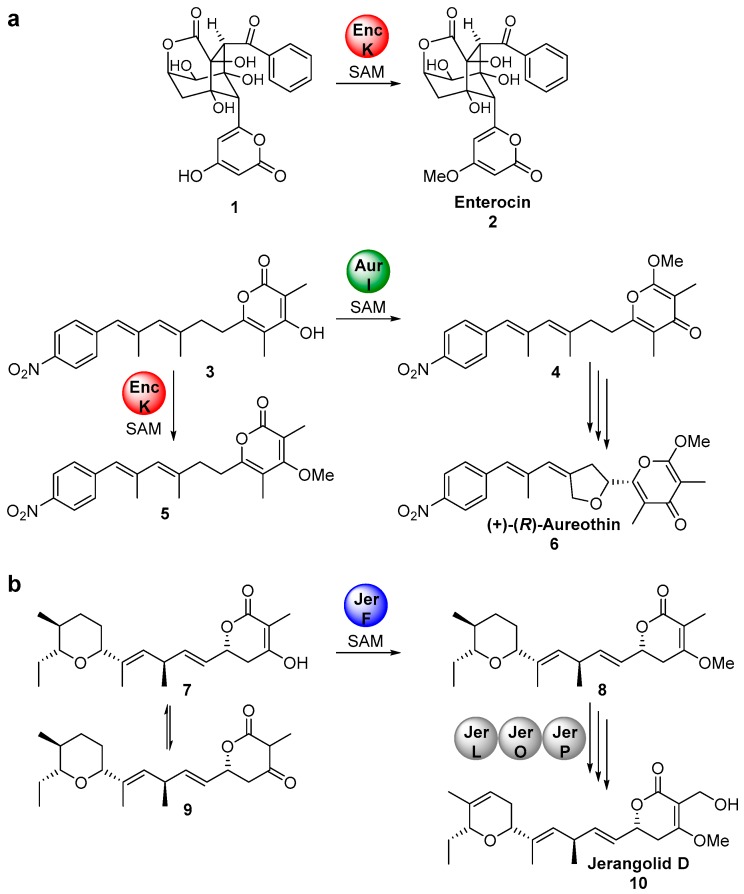
Enterocin (**2**), (+)-(R)-aureothin (**6**) and jerangolid D (**10**) are examples for biologically active bacterial polyketides harbouring *O*-methylated pyrone structures. (**a**) The SAM-dependent *O*-methyltransferases AurI and EncK are known to methylate 4-hydroxyl-pyrones **1** and **3**, respectively; (**b**) JerF is supposed to methylate the enol tautomer **7** in the course of jerangolid biosynthesis [6,7,8,9].

*O*-Methylated pyrones occur in polyketide natural products with highly interesting biological activity such as enterocin (**2**) and (+)-(*R*)-aureothin (**6**), which are produced by *Streptomyces thioluteus* and *Streptomyces maritimus*, respectively (see Scheme 1). For both compounds, methyl transfer takes place during the final tailoring steps of their biosynthesis and is catalyzed by class I *S*-(5′-adenosyl)-l-methionine (SAM)-dependent *O*-methyltransferases (*O*-MTs; EncK for **2**, AurI for **6**) [3]. Although EncK and AurI possess very high sequence similarity, they react with different chemoselectivity on the same structural element [4,5].

Extensive studies by Hertweck and Moore showed that AurI methylates the enol in **3** to form the γ-pyrone **4**, whereas EncK methylates the 5-hydroxyl group of desmethyl-5-deoxyenterocin (**1**) to form the γ-pyrone **2** [10,11,12]. EncK was able to complement *O*-methylation activity in a Δ*aurH* mutant of the aureothin producer. Interestingly, this supplemented strain produced the aureothin derivative **5**, showing that EnkC keeps its natural 4-*O*-selectivity for methylation while at the same time being highly substrate tolerant. Such *O*-methyltransferases are highly interesting candidates for application in combinatorial biosynthesis [5].

Another promising approach for accessing novel natural product derivatives is the application of biosynthesis enzymes for chemoenzymatic synthesis [3,13]. Methyltransferases are particularly attractive candidates for this purpose. Not only do they introduce specific methylation patterns on complex structures and thus enable streamlining a synthetic route by exploiting their high chemo- and regioselectivity. Furthermore, it has been shown on several examples that the tolerance of MTs regarding the transferred group can be used to bring in alternative residues. For example, propargyl groups can be introduced onto specific positions and later addressed for biorthogonal coupling reactions like click chemistry [14,15,16,17]. Apart from natural product synthesis, this strategy has also been used for site specific labelling of biomolecules like DNA, RNA or proteins [18,19,20,21].

The jerangolids (jerangolid D (**10**) is shown in Scheme 1) are myxobacterial, reduced polyketide natural products with unusual structural elements, resulting from extensive tailoring of the primary polyketide synthase (PKS) product. They contain a 3-(hydroxymethyl)-4-methoxy-5,6-dihydro-2*H*-pyran-2-one, which is formed via action of the PKS-thioesterase (TE) domain followed by the *O*-MT JerF, the Rieske-FeS-cluster protein JerL and the monooxygenase JerO [6]. JerF is responsible for the formation of a methylenolether starting from a ketone, thus leading to a cyclic, non-phenolic methyl ether. Apart from the two above-mentioned examples from enterocin (**2**) and aureothin (**6**) biosynthesis, no *O*-MTs that form methylated pyrones have been characterised yet.

## 2. Results

### 2.1. Synthesis of Substrate Surrogates

**Scheme 2:**
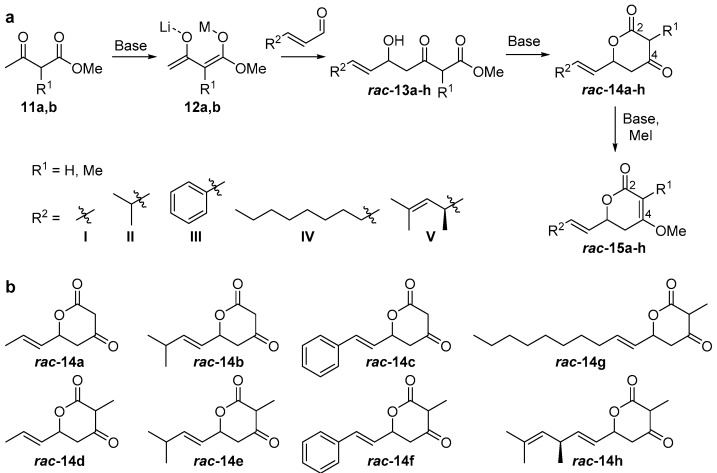
(**a**) General synthetic route to JerF precursor surrogates ***rac****-***14a**–**h**. Product surrogates were obtained by chemical methylation using MeI and base. For exact reaction conditions, see the materials and methods section; (**b**) Substrate surrogates that were applied in the assays.

To confirm the postulated biosynthetic role of JerF and to evaluate its potential for chemoenzymatic synthesis, we set out to investigate the catalytic activity of JerF in vitro. Synthetic precursor surrogates of varying complexity (***rac****-***14a**–**h**) were synthesised that covered a broad structural space (Scheme 2; Figures S1–S26). Two series of compounds were obtained, containing either the 3-methyl-6-vinyldihydro-2*H*-pyran-2,4(3*H*)-dione (***rac****-***14d**–**h**) that is present in the biosynthetic precursor or 3-desmethyl analogs (***rac****-***14a**–**c**). All substrates were readily available by vinylogous aldol reaction of β-ketoester enolates **11a** or **11b**, respectively, followed by lactonization under basic conditions (Scheme 2a) [22,23,24,25]. The relative orientation of the substituents on ring positions 3 and 6 in ***rac****-***14d**–**h** was shown to be predominantly *syn*, according to ^1^H-NMR spectroscopy and NOE correlation spectroscopy (see NMR spectra and Figure S26 in the Supplementary Materials). The methylenolethers ***rac****-***15a**–**g** were synthesised from ***rac****-***14a**–**g** by *O*-methylation using MeI and K_2_CO_3_ or NaH, respectively.

### 2.2. Cloning, Expression and Establishment of Assay Conditions

A codon-optimised gene of *jerF* (GenBank accession number: ABK32292.1) was cloned into *pET28a(+)*, *pET20b(+)* and *pCOLDI* plasmids for overexpression in *E. coli* BL21 (DE3). Only for the C-terminally His_6_-tagged fusion protein (derived from expression of *jerF-pET20b(+)*), minor amounts of purified but catalytically inactive protein were obtained after Ni-affinity chromatography. We therefore decided to conduct bioconversion experiments with the cell-free extracts.

**Figure 1:**
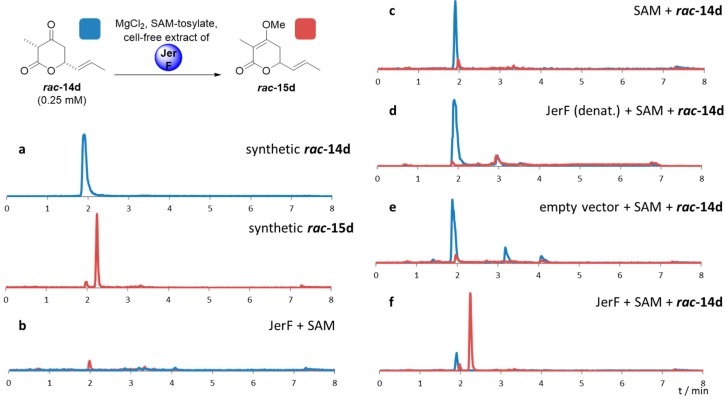
HPLC-MS analysis of in vitro conversions catalyzed by JerF. Chromatograms are shown for *m*/*z* = 191 (blue trace) and *m/z* = 205 (red trace). (**a**) synthetic ***rac****-***14d** and synthetic ***rac****-***15d**; (**b**) cell-free extract containing JerF with 0.94 mM SAM tosylate in reaction buffer (40 mM Tris HCl, 100 mM NaCl, 5 mM MgCl_2_, pH 8.8); (**c**) reaction buffer with SAM tosylate and ***rac****-***14d**; (**d**) heat-inactivated cell-free extract containing JerF in reaction buffer with SAM tosylate and ***rac****-***14d**; (**e**) cell-free extract from expression of *pCOLD-I* (devoid of *jerF*) with SAM tosylate and ***rac****-***14d**; (**f**) cell-free extract from expression of *jerF_pCOLD-I* with SAM tosylate and ***rac****-***14d**. Conditions of the conversion assay in (**f**): 0.25 mM ***rac****-***14d**, 5 mM MgCl_2_, 1.0 mM SAM-tosylate, 0.2 mL cell-free extract (2.7 mg/mL), 28 °C, 16 h; ***rac***-**14d**: [M + Na]^+^ = 191.0684, ***rac***-**15d**: [M + Na]^+^ = 205.0841.

Catalytically active cell-free extracts were obtained from *jerF-pCOLDI* expression in *E. coli* BL21 (DE3) at 16 °C for 16 h in the presence of 0.1 mM IPTG. Cell disruption was achieved by sonication on ice in pH 8.8 reaction buffer (40 mM Tris HCl, 100 mM NaCl). Initial bioconversion experiments were carried out with the racemic mixture of substrate surrogates ***rac***-**14d** (0.25 mM), 5 mM MgCl_2_ and 0.97 mM SAM tosylate for 16 h at 28 °C (Figure 1, for unprocessed spectra see Figures S48–S53) [26,27]. HPLC-MS analysis showed that ***rac***-**14d** was almost completely converted into the respective *O*-methylated product mixture ***rac***-**15d** (Figure 1f).

To unambiguously assign the methylation activity to the *jerF* gene product, we conducted a series of control experiments. Methylation occurred only in the presence of MgCl_2_, SAM tosylate and the lysate from the *jerF-pColdI* expression (Figure 1f). If any of these components was left out, no formation of ***rac****-***15d** was obtained (Figure 1b,c). The same was true if the lysate was denatured by heat treatment prior assaying, which attributes the activity to a component of the cell lysate (Figure 1d). The lysate of a *pColdI* vector expression (devoid of *jerF*) in *E. coli* BL21 did also not cause the formation of ***rac****-***15d**, clearly highlighting that the expression product of *jerF* is responsible for the observed activity (Figure 1e).

### 2.3. Comparative Assaying of Synthetic Substrates and Assay Upscaling

**Figure 2:**
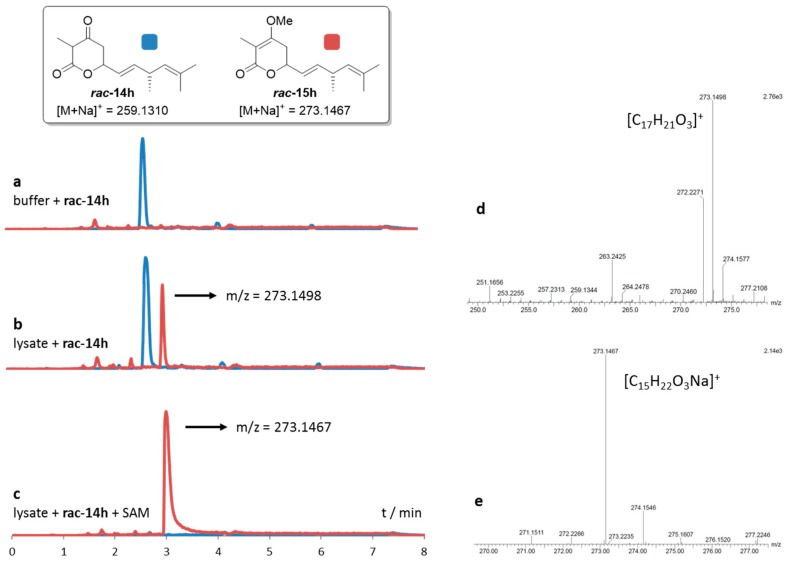
HPLC-MS analysis of in vitro conversions of ***rac-*14h** catalyzed by JerF. Chromatograms are shown for *m*/*z* = 259 (blue trace) and *m*/*z* = 273 (red trace). (**a**) synthetic ***rac-*14h** after overnight incubation in buffer; (**b**) incubation of ***rac-*14h** with cell-free extract containing JerF; (**c**) incubation of ***rac-*14h** with cell-free extract containing JerF and SAM tosylate in reaction buffer. The minimal conversion is probably caused by residual amounts of SAM in the cell-free extract. (**d**) HR-MS analysis of the assay product of (**b**); (**e**) HR-MS analysis of the assay product of (**c**); Conditions of the conversion assay: 0.25 mM ***rac-*14h**, 5 mM MgCl_2_, 1.0 mM SAM-tosylate, 0.2 mL cell-free extract (3.0 mg/mL), 28 °C, 16 h, pH 8.8.

Under the conditions established for ***rac****-***14d**, compound ***rac****-***14h** was completely methylated, highlighting the broad substrate tolerance of JerF (Figure 2). The higher conversion compared to ***rac****-***14d** reflects the closer structural similarity of ***rac****-***14h** to the proposed biosynthetic precursor **9**.

Insertion of the other synthetic substrate surrogates ***rac-*14b**, ***rac-*****14c** and ***rac-*14e-g** under similar conditions showed partial conversion of all substrates containing a 3-Me group (***rac-*14e-g**) and full conversion of the non-branched substrates ***rac-*14b** and ***rac-*14c** (Figures S54–S58). In all cases, an exclusive methylation on the 4-O and no reaction on 2-O or C-3 occurred, showing that JerF acts highly chemo- and regioselectively. However, for the non-branched substrates ***rac-*14b** and ***rac-*14c**, the absolute amount of formed product was unexpectedly low.

Overnight incubation of compounds ***rac-*14b**, ***rac-*****14c** and ***rac-*14e**–**g** in the absence of JerF revealed that all substrates undergo slow, spontaneous degradation at pH 8.8. This trend is more pronounced for the non-branched lactones ***rac-*14b** and ***rac-*14c** and seems to be accelerated by uncharacterised components of the lysate. Accordingly, attempts to conduct the reaction with ***rac-*14d** on the semi-preparative scale (up to 7 mg starting material) led to hardly reproducible results and yields below 10%.

**Figure 3:**
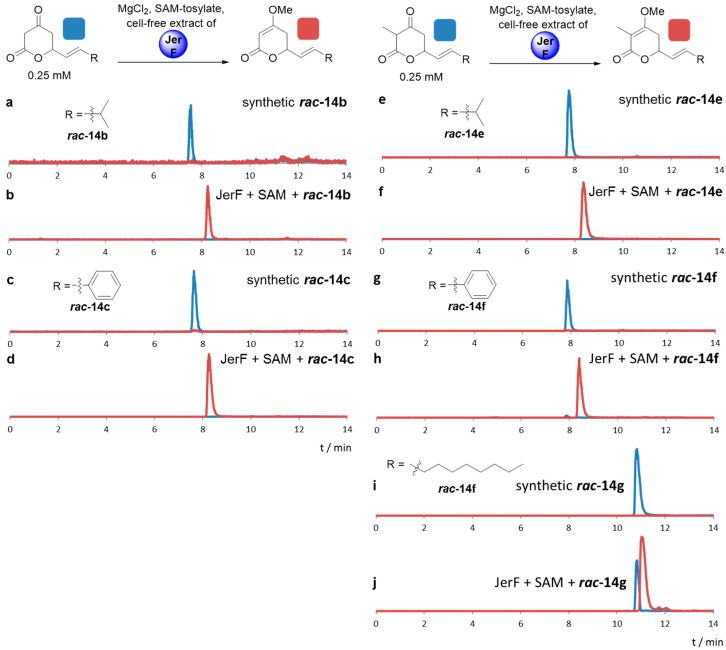
HPLC-MS analysis of in vitro conversions catalyzed by JerF. (**a**–**j**) Chromatograms are shown for *m/z* of substrates ***rac***-**14b**, ***rac***-**14c** and ***rac***-**14e**–**g** (blue traces) and the respective *O*-methylated products ***rac***-**15b**, ***rac***-**15c** and ***rac***-**15e**–**g** (red traces). General conditions of the conversion assays: cell-free extract of the JerF expression (7.8 mg/mL) with 4.18 mM SAM tosylate, 5 mM MgCl_2_ and 0.25 mM substrate incubated at 28 °C for 20 h in reaction buffer (25 mM HEPES, 100 mM NaCl, pH 7.5). ***rac***-**14b**: [M + Na]^+^ = 205.0820; ***rac***-**14e/*rac***-**15b**: [M + Na]^+^ = 219.0976; ***rac***-**15e**: [M + Na]^+^ = 233.1133; ***rac***-**14c**: [M + Na]^+^ = 239.0663; ***rac***-**14f/*rac***-**15c**: [M + Na]^+^ = 253.0820; ***rac***-**15f**: [M + Na]^+^ = 267.0976; ***rac***-**14g**: [M + Na]^+^ = 289.1759; ***rac***-**15g**: [M + Na]^+^ = 303.1915.

A markedly increased stability of the lactones as well as the corresponding methylenolethers ***rac***-**15b**, ***rac***-**15c** and ***rac***-**15e**–**g** was observed at near-neutral pH. In comparative enzymatic assays with compound mixture ***rac****-***14e** and JerF at different pH values, the best results in terms of conversion and compound stability were observed at pH 7.5 (Figure S59). The experiments were thus repeated at this pH value with substrates ***rac***-**14b**, ***rac***-**14c** and ***rac***-**14e**–**g**. Full conversion was reproducibly obtained for compounds ***rac***-**14b**, ***rac***-**14c**, ***rac***-**14e** and ***rac***-**14f** (Figure 3, for unprocessed spectra see Figures S60–S64). The compound mixture ***rac***-**14g** was also methylated to a large extend, however the presence of small amounts of starting material was still visible. The 3-desmethyl substrates ***rac***-**14b** and ***rac***-**14c** were fully methylated according to HPLC-MS analysis. However, complete degradation of ***rac***-**14b** and ***rac***-**14c** without any conversion into ***rac***-**15b** and ***rac***-**15c** was observed in some repetitions of the experiment, indicating that destructive side reactions caused by the lysate could be partially responsible for this positive result.

Reaction upscaling was also more successful at pH 7.5 and gave reproducible results (Table 1, Figures S43–S47). Approximately 4 mg of compounds ***rac***-**14c**, ***rac***-**14e** and ***rac***-**14f** were individually incubated with the cell-free extract from a *jerF* expression in a total assay volume of 10 mL. Conversions of 27%–42% into the respective methylenolethers ***rac***-**15c**, ***rac***-**15e** and ***rac***-**15f** were obtained. The crude products of ***rac***-**15e** and ***rac***-**15f** were partially purified by column chromatography on silica gel. In both cases, an aliphatic impurity was co-purified, which could not be removed.

The crude products of the semi-preparative conversions as well as the samples from column chromatography were analysed by chiral HPLC. In the cases of ***rac***-**14c** and ***rac***-**14e**, the racemic starting material was converted into product enriched in one stereoisomer with an enantiomeric ratio of 92:8 and 71:29, respectively (Table 1). For ***rac***-**14f**, an only negligible enantiomeric excess was observed. JerF thus discriminates between the inserted stereoisomers, however, with a strong dependence on the substrate structure.

## 3. Discussion

**Scheme 3:**
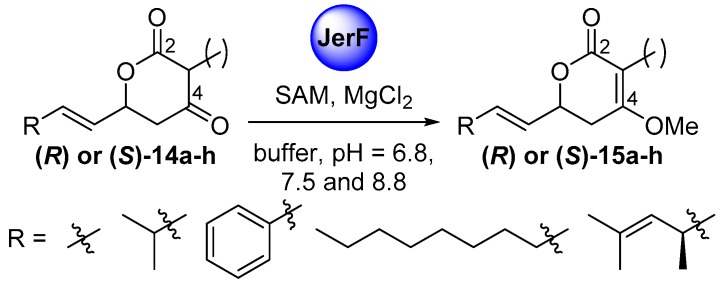
JerF shows broad substrate tolerance in the reaction with diverse 6-vinyldihydro-2*H*-pyran-2,4(3*H*)-diones ***rac***-**14a**–**h**.

We were able to characterise the *O*-methyltransferase JerF from jerangolid biosynthesis by assaying of the enzyme in bioconversion experiments with synthetic substrate surrogates. JerF is the first characterised case of an *O*-methyltransferase that forms a cyclic, non-aromatic methylenolether. The enzyme shows promising substrate tolerance. It accepts aliphatic and aromatic residues (R in Scheme 3) of varying size as well as substrates that lack the methyl group on C-3, which is present on the natural precursor. Furthermore, the enzyme is fully selective for methylation on 4-O and is not reactive towards the other two potential methylation sites at 2-O and C-3. During chemical synthesis of substrates ***rac***-**15a**–**h**, only C-3-methylated side products were obtained, suggesting that an inherent selectivity for 4-*O*-methylation exists under the conditions applied. In this context, it would be interesting to evaluate if the *O*-MT AurI keeps its confirmed 2-*O*-selectivity in the reaction with this kind of substrates.

Reactions with the enzyme could be conveniently performed using the cell-free extract from a *jerF* expression. Reactions on the analytical scale with substrates ***rac***-**14a**–**h** proceeded with high to complete conversion. Problems arising from slow spontaneous degradation during the reaction could be reduced by performing the reaction at pH 7.5.

Upscaling of the enzymatic reactions with substrate mixtures ***rac***-**14c**, ***rac***-**14e** and ***rac***-**14f** was successful at pH 7.5, giving conversions of 27%–42%. A further optimisation of the reaction conditions will probably improve this result. It is furthermore known that SAM-dependent MTs are often inhibited by *S*-adenosylhomocysteine (SAH) that is formed during the reaction. Addition of a SAH-hydrolase or SAH nucleosidase could thus also be helpful. Analysis of the semi-preparative scale conversions by chiral HPLC revealed a discrimination of JerF between the inserted stereoisomers. The degree of selectivity strongly depended on the constitution of the inserted substrates.

The insights gained about relevant features of JerF like substrate tolerance and chemoselectivity suggest further investigations on its applicability in chemoenzymatic synthesis. Future studies will concentrate on a thorough optimisation of the enzyme overexpression and the reaction conditions on the analytical and the (semi)preparative scale. Further studies on its substrate tolerance will help to evaluate the scope of the enzyme. Finally, the enzyme will be applied in the chemoenzymatic total synthesis of 4-methoxy-5,6-dihydro-2*H*-pyran-2-one-containing natural products.

## 4. Materials and Methods

### 4.1. General Information

#### 4.1.1. Chemistry Methods and Materials

All reactions were performed in oven dried glassware under an atmosphere of nitrogen gas unless otherwise stated. Dry solvents were purchased from Sigma-Aldrich Chemie GmbH (Steinheim, Germany) and Acros (Geel, Belgium) or taken out of a solvent system from M. Braun (Garchingen, Germany). Dry reagents were ordered from Sigma-Aldrich, Arcos, abcr GmbH (Karlsruhe, Germany) and Roth (Karlsruhe, Germany). NMR spectra were recorded with DRX-500, DPX-400 and AVANCE-400 instruments (Bruker, Billerica, MA, USA) with the residual solvent signal as internal standard (CHCl_3_ = 7.26 ppm). multiplicities are described using the following abbreviations: s = singlet, d = doublet, t = triplet, q = quartet, m = multiplet, b = broad. ^13^C-NMR spectra are reported as values in ppm relative to residual solvent signal (CHCl_3_ = 77.06 ppm) as internal standards. The multiplicities are elucidated using the distortionless enhancement by polarisation transfer (DEPT) spectral editing technique, with secondary pulses at 90° and 135°. Multiplicities are reported using the following abbreviations: q (quarternary carbon), t (tertiary carbon = methine), s (secondary carbon = methylene), p = (primary carbon = methyl). High resolution mass spectra are obtained with a Micromass LCT via loop-mode injection from an Alliance 2695 HPLC system (Waters, Milford, MA, USA). Alternatively, a Micromass Q-TOF in combination with a Waters Acquity Ultra performance LC system is employed. Ionisation is achieved by ESI or APCI. Modes of ionisation, calculated and found mass are given. Reversed phase-HPLC-applications were performed with membrane-filtrated and double distilled water as well as commercial available HPLC-grade solvents (methanol or acetonitrile). Semi-preparative HPLC was performed with a Merck Hitachi HPLC system (Darmstadt, Germany; Pump L-7150, Interface D-7000, Diode Array Detector L-7450) under use of C18-SP stationary phase. Solvents, columns, operating procedures and retention times (t_R_) are given with the corresponding experimental and analytical data. (Abbreviations: PE = petroleum ether; EtOAc = ethyl acetate). Chiral HPLC-applications were performed with a Waters Alliance HPLC (Waters 2695 Separation Module, Waters 2487 Dual λ Absorbance Detector). In all cases the flow was 0.8 mL/min and the detection wavelength was 215 nm. Gradient conditions were: 1: OD-3, *n*-hexane:*i*-PrOH = 93:7; 2: AD-H, *n*-hexane:*i-*PrOH = 90:10; 3: AD-H, *n*-hexane:*i-*PrOH = 95:5; 4: OD-3, *n*-hexane:*i-*PrOH = 80:20; 5: OD-3, *n*-hexane:*i-*PrOH = 85:15; 6: AD-H, *n*-hexane:*i-*PrOH = 90:10. The retention times (t_R_) are given with the corresponding experimental and analytical data (abbreviations for columns: OD-3: Daicel Chiralcel^®^ OD-3; AD-H: Daicel Chiralpak^®^ AD-H). Samples were applied in form of 10 µL of membrane-filtrated solution in a concentration of approximately 1 mg/mL in *n*-hexane:*i*PrOH = 85:15.

#### 4.1.2. Biochemistry Methods and Materials

All chemicals and antibiotics were purchased from Sigma-Aldrich and Roth. Cell disruption was conducted by sonication (Sonoplus Typ UW3100) from Bandelin (Berlin, Germany). His-bind nickel chelate chromatography resin was purchased from Novagen. Millipore Amicon^®^ ultra centrifugal filters (10,000 and 30,000 MW Cut-off) and PD-10 desalting columns from GE Healthcare (Buckinghamshire, UK) were used for protein concentration and buffer exchange respectively.

### 4.2. Synthesis of Substrate Surrogates

#### 4.2.1. General Procedures

##### Aldol Reaction

A solution of LDA was freshly prepared by adding *n*-BuLi (2.5 M in hexane, 2.5 equiv.) to diisopropylethylamine (0.7 M, 2.5 equiv.) in THF at −78 °C. The solution was stirred at room temperature for 30 min and after cooling to −78 °C, DMPU (1.0 equiv.) was added. Methyl-2-methyl-3-oxobutanoate or methyl-3-oxobutanoate (1 M, 1.0 equiv.), respectively, in THF was added and the solution was stirred for further 50 min. The aldehyde (1.1 equiv.) was added and the solution stirred for 2 h. The reaction was quenched by addition of 2 M HCl. After separation of the layers, the aqueous layer was extracted by Et_2_O and the combined organic layers were dried over MgSO_4_. The solvent was removed in vacuo.

##### Lactonization

The product of the aldol reaction was dissolved in 1 M KOH and stirred at room temperature for 5 h. After cooling to 0 °C, 2 M HCl was added until a pH value of 0 was reached. The resulting solid was filtrated, washed with H_2_O and the desired product purified by column chromatography.

##### *O*-Methylation of the Dihydropyran-2,4-diones

After dissolving the lactone (0.2 M, 1.0 equiv.) in DMF_abs_, the solution was cooled to 0 °C and MeI (1.0 equiv.) and K_2_CO_3_ (1.5 equiv.) were added. After 1 h, the solution was warmed to room temperature and stirred overnight. The layers were separated and the aqueous layer was extracted with EtOAc. The combined organic layers were washed with brine, dried over MgSO_4_ and the solvent was removed in vacuo. The crude product was purified by column chromatography.

##### *O*-Methylation of the 3-Methyldihydropyran-2,4-diones

After dissolving the lactone (0.2 M, 1.0 equiv.) in THF_abs_, the solution was cooled to 0 °C and NaH (60% in mineral oil, 1.3 equiv.) was added. After 1 h, MeI (1.2 equiv.) was added and the solution was warmed to room temperature. After stirring overnight, the reaction was quenched by the addition of H_2_O. After separation of the layers, the aqueous layer was extracted with EtOAc, the combined organic layers were washed with brine and dried over MgSO_4_. The solvent was removed in vacuo and the crude product was purified by column chromatography.

#### 4.2.2. Substrate Synthesis

**Scheme 4:**
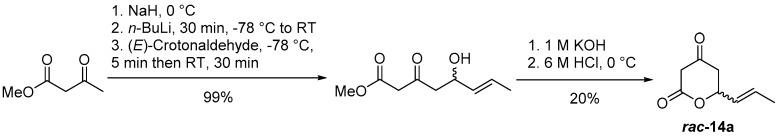
Synthesis of compound ***rac***-**14a** [28,29,30].

**Scheme 5:**
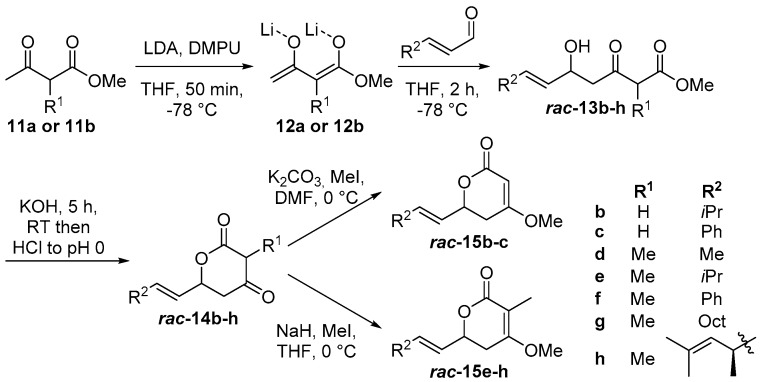
General synthetic route to substrates ***rac***-**14b**–**g** and reference compounds ***rac***-**15b**–**g**.

**Scheme 6:**
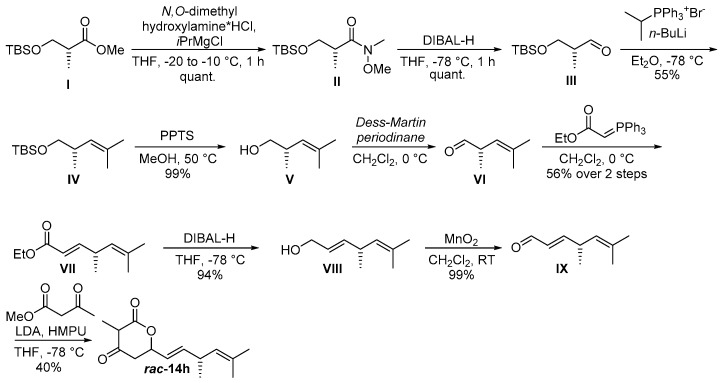
Synthesis of dione ***rac***-**14h** from TBS-protected *Roche ester* **I**.

Compounds ***rac*-14a**–**g** and ***rac*-15b**–**h** were synthesised according to the route shown in Scheme 4, Scheme 5 and Scheme 6.

##### (*E*)-6-(Prop-1-en-1-yl)dihydro-2*H*-pyran-2,4(3*H*)-dione (***rac***-**14a**)

Under nitrogen atmosphere, sodium hydride (60% suspension, 900 mg, 22.5 mmol, 1.2 equiv.) was suspended in 40 mL dry THF and cooled to 0 °C. To this reaction mixture, methyl acetoacetate (2.0 mL, 18.7 mmol, 1.0 equiv.) was added dropwise. After gas evolution had ceased, the reaction was cooled to −78 °C followed by dropwise addition of *n*-BuLi (8.3 mL, 20.6 mmol, 1.1 equiv., 2.5 M in hexanes). The reaction was allowed to warm to 0 °C for 30 min and then cooled again to −78 °C. (*E*)-crotonaldehyde (1.7 mL, 20.6 mmol, 1.1 equiv.) was added dropwise over 5 min and the reaction was stirred for 30 min at r.t. The reaction was quenched by addition of 20 mL saturated NH_4_Cl/H_2_O (1:1) at 0 °C. The aqueous solution was washed twice with 50 mL EtOAc and then acidified to pH 1 using concentrated hydrochloric acid. The resulting precipitate was dissolved in 20 mL EtOAc and the aqueous phase was extracted twice with 20 mL EtOAc. The combined organic phases were dried over Na_2_SO_4_ and filtered. The solvents were removed under reduced pressure. Compound ***rac***-**14a** was obtained as a pale-yellow solid (20%, 588 mg, 4.50 mmol).

##### Methyl-(*E*)-5-hydroxy-2-methyl-3-oxooct-6-enoate (***rac***-**13d**)

Under argon atmosphere, diisopropylamine (1.9 mL, 13.5 mmol, 2.5 equiv.) was dissolved in 20 mL dry THF and cooled to −78 °C. To this solution, *n*-BuLi (5.3 mL, 13.5 mmol, 2.5 M in hexanes, 2.5 equiv.) was added dropwise. The reaction was allowed to warm to 0 °C for 30 min. At −78 °C, DMPU (670 µL, 5.40 mmol, 1.0 equiv.) was added dropwise and the resulting mixture was stirred for 30 min. Methyl-3-oxobutanoate (702 µL, 5.40 mmol, 1.0 equiv.) was dissolved in 5 mL dry THF and this solution was added to the reaction mixture followed by stirring for 50 min at −78 °C. (*E*)-crotonaldehyde (490 µL, 5.90 mmol, 1.1 equiv.) was then added followed by stirring for 2 h at −78 °C. The reaction was quenched by addition of 30 mL 2 M hydrochloric acid at −78 °C and allowed to warm to r.t. The aqueous phase was extracted three times with 50 mL Et_2_O. The combined organic phases were dried over Na_2_SO_4_ and filtered. The solvents were removed under reduced pressure. 1.06 g of crude ***rac***-**13d** were obtained and used without further purification.

##### (*E*)-3-Methyl-6-(prop-1-en-1-yl)dihydro-2*H*-pyran-2,4(3*H*)-dione (***rac***-**14d**)

Crude methyl ester ***rac***-**13d** (414 mg, 2.10 mmol, 1.0 equiv.) was dissolved in 20 mL 1 M potassium hydroxide solution and was stirred at r.t. for 7 h. The solution was cooled to 0 °C followed by addition of 6 M hydrochloric acid to pH 1.0. The resulting precipitate was filtered using a glass frit and the obtained crystals were dried under reduced pressure to give compound ***rac***-**14d** as yellow crystals (263 mg, 1.50 mmol, 70%, syn:anti = 5:1).

##### (*E*)-4-Methoxy-3-methyl-6-(prop-1-en-1-yl)-5,6-dihydro-2*H*-pyran-2-one (***rac***-**15d**)

Under nitrogen atmosphere, (*E*)-3-Methyl-6-(prop-1-en-1-yl)dihydro-2*H*-pyran-2,4(3*H*)-dione (**25a**, 50.0 mg, 300 μmol, 1.0 equiv.) was dissolved in 3.0 mL dry THF and the resulting solution was cooled to 0 °C. NaH (60% suspension, 13.9 mg, 350 μmol, 1.2 equiv.) was added and the reaction mixture was stirred for 30 min followed by addition of MeI (22 μL, 360 μmol, 1.2 equiv.). The reaction mixture was stirred for 1 h at r.t. and then quenched by the addition of 3 mL H_2_O. The aqueous phase was extracted three times with 10 mL EtOAc. The combined organic phases were washed twice with brine and then dried over Na_2_SO_4_. After filtration, the solvents were removed under reduced pressure. Purification by flash column chromatography (petroleum ether (PE/EtOAc = 4:1 to 2:1) yielded the product (***rac***-**15d**, 30 mg, 165 μmol, 55%) as a pale-yellow solid. 

##### (*E*)-6-(3-Methylbut-1-en-1-yl)dihydro-2*H*-pyran-2,4(3*H*)-dione (***rac***-**14b**)

Following the general procedures 1 and 2, dione ***rac***-**14b** was prepared from LDA (816 mg, 7.65 mmol, 2.5 equiv.), DMPU (333 mL, 2.78 mmol, 1.0 equiv.), methyl-3-oxobutanoat (299 mL, 2.78 mmol, 1.0 equiv.) and (*E*)-4-methylpent-2-enal (300 mg, 3.06 mmol, 1.1 equiv.). After column chromatography on silica gel (PE/EtOAc = 5:1 → 2:1), the mixture of the diones ***rac***-**14b** (113 mg, 622 μmol, 20%) was obtained as a yellow solid.

##### (*E*)-4-Methoxy-6-(3-methylbut-1-en-1-yl)-5,6-dihydro-2*H*-pyran-2-one (***rac***-**15b**)

Following the general procedure 3, methoxy-pyran-2-one ***rac***-**15b** was prepared from dione ***rac***-**14b** (50.0 mg, 270 μmol, 1.0 equiv.), MeI (17.0 μL, 270 μmol, 1.0 equiv.) and K_2_CO_3_ (56.0 mg, 400 μmol, 1.5 equiv.). After column chromatography on silica gel (PE/EtOAc = 4:1 → 2:1), the mixture of the methoxy-pyran-2-ones ***rac***-**15b** (9.40 mg, 48.0 μmol, 18%) was obtained as a yellow solid.

##### (*E*)-3-Methyl-6-(3-methylbut-1-en-1-yl)dihydro-2*H*-pyran-2,4(3*H*)-dione (***rac***-**14e**)

Following the general procedures 1 and 2, dione ***rac***-**14e** was prepared from LDA (546 mg, 5.10 mmol, 2.5 equiv.), DMPU (244 mL, 2.04 mmol, 1.0 equiv.), methyl-2-methyl-3-oxobutanoate (265 mg, 2.04 mmol, 1.0 equiv.) und (*E*)-4-methylpent-2-enal (200 mg, 2.04 mmol, 1.0 equiv.). After column chromatography on silica gel (PE/EtOAc = 2:1), the mixture of the diones ***rac***-**14e** (222 mg, 1.13 mmol, 55%, *d*.*r*. = 10:1) was obtained as a yellow solid.

##### (*E*)-4-Methoxy-3-methyl-6-(3-methylbut-1-en-1-yl)-5,6-dihydro-2*H*-pyran- 2-one (***rac***-**15e**)

Following the general procedure 4, methoxy-pyran-2-one ***rac***-**15e** was prepared from dione ***rac***-**14e** (*E*)-3-methyl-6-(3-methylbut-1-en-1-yl)dihydro-*2H*-pyran-2,4(*3H*)-dion (50.0 mg, 260 μmol, 1.0 equiv.), NaH (13.2 mg, 330 μmol, 1.3 equiv.) and MeI (20.0 μL, 312 μmol, 1.2 equiv.). After column chromatography on silica gel (PE/EtOAc = 5:1 → 2:1), the mixture of the methoxy-pyran-2-ones ***rac***-**15e** (5.20 mg, 25.0 μmol, 10%) was obtained as a yellow solid.

##### Methyl-(*E*)-5-hydroxy-3-oxo-7-phenylhept-6-enoate (***rac***-**13c**)

NaH (60% in mineral oil, 440 mg, 11.0 mmol, 1.1 equiv.) was solved in 25 mL THF_abs_ at 0 °C and methyl-3-oxobutanoate (1.08 mL, 10.0 mmol, 1.0 equiv.) was added. After 20 min, the solution was cooled to −78 °C, *n*-BuLi (2.5 M in hexane, 4.40 mL, 11.0 mmol, 1.1 equiv.) was added and it was stirred for 1 h at this temperature. After adding cinnamic aldehyde (1.39 mL, 11.0 mmol, 1.1 equiv.) to the reaction mixture, the solution was stirred for 3 h at −78 °C. The reaction was quenched by the addition of 2 M HCl. After separation of the layers, the aqueous layer was extracted with Et_2_O (3 × 50 mL), the combined organic layers were dried over MgSO_4_ and the solvent was removed in vacuo. After purification by column chromatography on silica gel (PE/EtOAc = 4:1), the desired alcohol ***rac***-**13c** (632 mg, 2.72 mmol, 27% yield) was obtained as an orange solution.

##### (*E*)-6-Styryldihydro-2*H*-pyran-2,4(3*H*)-dione (***rac***-**14c**)

Methyl-(*E*)-5-hydroxy-3-oxo-7-phenylhept-6-enoate (***rac***-**13c**, 100 mg, 430 μmol, 1.0 equiv.) was solved in 2 mL MeOH_abs_ and K_2_CO_3_ (89.3 mg, 650 μmol, 1.5 equiv.) was added at room temperature. The reaction mixture was stirred for 3 h at r.t., the resulting solid was filtered and washed with H_2_O. After purification by column chromatography on silica gel (PE/EtOAc = 4:1 → 2:1), the desired lactone ***rac***-**13c** (58.8 mg, 272 μmol, 63% yield) was obtained as a colourless solid.

##### (*E*)-4-Methoxy-6-styryl-5,6-dihydro-2*H*-pyran-2-one (***rac***-**15c**)

Following the general procedure 3, methoxy-pyran-2-one ***rac***-**15c** was prepared from dione ***rac***-**14c** (50.0 mg, 230 μmol, 1.0 equiv.), MeI (14.5 μL, 230 μmol, 1.0 equiv.) and K_2_CO_3_ (47.7 mg, 350 μmol, 1.5 equiv.). After column chromatography on silica gel (PE/EtOAc = 4:1 → 1:1), the mixture of the methoxypyran-2-ones ***rac***-**15c** (17.0 mg, 130 μmol, 56%) was obtained as a yellow solid.

##### (*E*)-3-Methyl-6-styryldihydro-2*H*-pyran-2,4-(3*H*)-dione (***rac***-**14f**)

Following the general procedures 1 and 2, dione ***rac***-**14f** was prepared from LDA (205 mg, 1.92 mmol, 2.5 equiv.), DMPU (118 mL, 799 μmol, 1.0 equiv.), methyl-2-methyl-3-oxobutanoate (100 mg, 0.77 mmol, 1.0 equiv.) and cinnamic aldehyde (101 μL, 799 μmol, 1.1 equiv.). After column chromatography on silica gel (PE/EtOAc = 2:1), the mixture of the diones ***rac***-**14f** (67.3 mg, 292 μmol, 37%) was obtained as a white solid.

##### (*E*)-4-Methoxy-3-methyl-6-styryl-5,6-dihydro-2*H*-pyran-2-one (***rac***-**15f**)

Following the general procedure 4, methoxy-pyran-2-one ***rac***-**15f** was prepared from dione ***rac***-**14f** (25.0 mg, 109 μmol, 1.0 equiv.), NaH (6.00 mg, 131 μmol, 1.2 equiv.) and MeI (8.00 μL, 131 μmol, 1.2 equiv.). After column chromatography on silica gel (PE/EtOAc = 4:1 → 2:1), the mixture of the methoxypyran-2-ones ***rac***-**15f** (3.50 mg, 14.3 μmol, 13%) was obtained as a white solid.

##### (*E*)-6-(Dec-1-en-1-yl)-3-methyldihydro-2*H*-pyran-2,4(3*H*)-dione (***rac***-**14g**)

Following the general procedures 1 and 2, dione ***rac***-**14g** was prepared from LDA (402 mg, 3.75 mmol, 2.5 equiv.), DMPU (180 mL, 1.50 mmol, 1.0 equiv.), methyl-2-methyl-3-oxobutanoat (195 mg, 1.5 mmol, 1.0 equiv.) und (*E*)-undec-2-enal (278 mg, 1.70 mmol, 1.1 equiv.). After column chromatography on silica gel (PE/EtOAc = 10:1 → 1:1), the mixture of the diones ***rac***-**14g** (279 mg, 1.05 mmol, 62%, *d*.*r*. = 12.5:1) was obtained as a yellow solid.

##### (*E*)-6-(Dec-1-en-1-yl)-4-methoxy-3-methyl-5,6-dihydro-2*H*-pyran-2-on (***rac***-**15g**)

Following the general procedure 4, methoxy-pyran-2-one ***rac***-**15g** was prepared from dione ***rac***-**14f** (50.0 mg, 190 μmol, 1.0 equiv.), NaH (69.80 mg, 240 μmol, 1.3 equiv.) and MeI (14.0 μL, 230 μmol, 1.2 equiv.). After column chromatography on silica gel (PE/EtOAc = 5:1 → 2:1), the mixture of the methoxypyran-2-ones ***rac***-**15g** (50 mg, 30.3 μmol, 16%) was obtained as a white solid.

##### Methyl-*(R)*-3-((*tert*-Butyldimethylsilyl)oxy)-2-methylpropanoate (**I**)

(*R*)-Methyl-3-hydroxy-2-methylpropionate (10.0 mL, 90.0 mmol, 1.0 equiv.), DMAP (110 mg, 0.90 mmol, 0.01 equiv.) and imidazole (9.9 g, 144 mmol, 1.6 equiv.) were dissolved in 100 mL CH_2_Cl_2_. TBSCl was dissolved in 20 mL CH_2_Cl_2_ and added dropwise to the reaction mixture at 0 °C. The resulting suspension was stirred at r.t. for 1 h. The reaction was quenched by the addition of 150 mL H_2_O. The aqueous phase was extracted three times with 50 mL CH_2_Cl_2_. The combined organic phases were washed once with H_2_O and dried over MgSO_4_. After filtration, the solvent was removed under reduced pressure. The compound **I** was obtained as a pale-yellow oil (20.1 g) and was used without further purification.

##### *(R)*-3-((*tert*-butyldimethylsilyl)oxy)-*N*-methoxy-*N*,2-dimethylpropanamide (**II**)

TBS-protected *Roche ester* **I** (5.00 g, 21.51 mmol, 1 equiv.) was solved in 40 mL THF. *N*,*O*-Dimethylhydroxylamine hydrochloride (3.27 g, 33.6 mmol, 1.56 equiv.) was added and the resulting solution cooled to −20 °C. Isopropylmagnesium chloride (32 mL, 64.7 mmol, 3.01 equiv.) was added dropwise and stirred at −10 °C for 1 h. The reaction was quenched by the addition of 15 mL saturated NH_4_Cl solution and three times extracted with Et_2_O. The combined organic layers were dried over MgSO_4_ and the solvent removed in vacuo. After column chromatography on silica gel (15% Et_2_O in hexane), the amide **II** (5.62 g, 21.5 mmol, 99% yield) was obtained as a yellow oil.

##### *(R)*-3-((*tert*-Butyldimethylsilyl)oxy)-2-methylpropanal (**III**)

Amide **II** (2.51 g, 9.60 mmol, 1 equiv.) was solved in 10 mL THF and cooled to −78 °C. DIBAL-H solution (19.2 mL, 19.20 mmol, 2 equiv., 1 M in hexane) was added dropwise. After 1 h, the reaction was quenched by pouring it into 20 mL of a saturated solution of K-Na-tartrate and stirring for 1 h. The aqueous layer was three times extracted with Et_2_O and the combined organic layers were dried over MgSO_4_. After removal of the solvents under reduced pressure and column chromatography on silica gel, (PE/Et_2_O = 10:1) the aldehyde **III** (1.94g, 9.60 mmol, 99% yield) was obtained as a pale-yellow oil.

##### *(S)-tert*-Butyl-((2,4-dimethylpent-3-en-1-yl)oxy)dimethylsilane (**IV**)

To a suspension of bromo(isopropyl)triphenyl-λ5-phosphane (2.95 g, 7.65 mmol, 2 equiv.) in 15 mL THF was slowly added *n*-BuLi (2.60 mL, 6.50 mmol, 1.7 equiv., 2.5 M in hexane) at −78 °C. It was stirred at room temperature for 30 min until a dark-red colour appeared. It was cooled to −78 °C and aldehyde **III** (774 mg, 3.83 mmol, 1 equiv.) in a small amount of THF was added dropwise. After 16 h, water was added to the white suspension. The aqueous layer was three times extracted with CH_2_Cl_2_. The combined organic layers were dried over MgSO_4_ and the solvent removed in vacuo. After column chromatography on silica gel (PE), compound **IV** (600 mg, 2.63 mmol, 69% yield) was obtained.

##### (*S*)-2,4-Dimethylpent-3-en-1-ol (**V**)

Alkene **IV** (570 mg, 2.50 mmol, 1 equiv.) was solved in 10 mL MeOH and PPTS (3.76 g, 14.97 mmol, 6 equiv.) was added. After stirring for 24 h at 50 °C, the solvent was removed under reduced pressure. The residual solid was washed with 3 mL water and the aqueous layer was three times extracted with CH_2_Cl_2_. The combined organic layers were washed with saturated NaHCO_3_ solution and saturated NaCl solution and then dried over MgSO_4_. After removal of the solvent under reduced pressure and column chromatography on silica gel (PE/Et_2_O = 5:1), alcohol **V** (262 mg, 2.30 mmol, 92% yield) was obtained as a colourless oil.

##### (*R,E*)-Ethyl-4,6-dimethylhepta-2,5-dienoate (**VII**)

Alkohol **V** (560 mg, 4.90 mmol, 1 equiv.) was solved in 20 mL CH_2_Cl_2_ at 0 °C and NaHCO_3_ (1.24 g, 14.7 mmol, 3 equiv.) and DMP (3.83 g, 9.03 mmol, 1.8 equiv.) added. After 5 h, a cooled solution (0 °C) of ethyl-2-(triphenylphosphoranylidene)acetate (7.28 g, 20.89 mmol, 4.3 equiv.) in 5 mL CH_2_Cl_2_ was added and stirred for further 4 h. The solution was diluted with 20 mL CH_2_Cl_2_ and 10 mL of a saturated NaHCO_3_ solution was slowly added. The aqueous layer was three times extracted with CH_2_Cl_2_. The combined organic layers were washed with saturated Na_2_S_2_O_3_ solution, dried over MgSO_4_ and the solvent was removed under reduced pressure. After purification by column chromatography on silica gel (PE/Et_2_O = 20:1), ethyl ester **VII** (500 mg, 2.74 mmol, 56% yield over two steps) was obtained as a colourless oil.

##### (*R,E*)-4,6-Dimethylhepta-2,5-dien-1-ol (**VIII**)

Ester **VII** (65 mg, 0.36 mmol, 1 equiv.) was solved in 6 mL THF and cooled to −78 °C. A solution of DIBAL-H (1.10 mL, 1.10 mmol, 3.08 equiv., 1 M in hexane) that was cooled to 0 °C was added dropwise. It was stirred for 2 h at −78 °C, for 1 h at room temperature, 3 mL of a solution of saturated K-Na-tartrate solution was added together with 1 mL Et_2_O. It was stirred for further 23 h. The aqueous layer was three times extracted with Et_2_O. The combined organic layers were washed with saturated NaCl solution, dried over MgSO_4_ and the solvent was removed under reduced pressure. After column chromatography on silica gel (PE/Et_2_O = 5:1), alcohol **VIII** (47 mg, 0.34 mmol, 94% yield) was isolated.

##### (*R,E*)-4,6-Dimethylhepta-2,5-dienal (**IX**)

Alkohol **VIII** (20.5 mg, 150 µmol, 1 equiv.) was solved in 3 mL CH_2_Cl_2_ and MnO_2_ (240 mg, 2.76 mmol, 19 equiv.) added. After 24 h, the reaction mixture was filtrated over diatomaceous earth, the residual solid was washed with CH_2_Cl_2_ and the solvent was removed under reduced pressure. Aldehyde **IX** (20 mg, 0.15 mmol, 99% crude yield) was obtained without further purification.

##### ((*R*,*E*)-3,5-Dimethylhexa-1,4-dien-1-yl)-3-methyldihydro-2*H*-pyran-2,4(3*H*)-dione (***rac***-**14h**)

Under argon atmosphere, diisopropylamine (52 µL, 0.37 mmol, 2.5 equiv.) was dissolved in 0.5 mL dry THF and the resulting solution was cooled to −78 °C. To this solution, *n*-BuLi (150 µL, 0.37 mmol, 2.5 M in hexanes, 2.5 equiv.) was added dropwise. The reaction was allowed to warm to 0 °C for 30 min. At −78 °C, DMPU (19 µL, 0.15 mmol, 1.0 equiv.) was added dropwise and the resulting mixture was stirred for 30 min. Methyl-3-oxobutanoate (19.4 mg, 0.15 mmol, 1.0 equiv.) was dissolved in 250 µL dry THF and this solution was added to the reaction mixture followed by stirring for 1 h at −78 °C. Aldehyde **IX** (21 mg, 0.15 mmol, 1.0 equiv.), dissolved in 250 µL dry THF, was then added followed by stirring for 2 h at −78 °C. The reaction was quenched by addition of 1 mL 3 M hydrochloric acid at −78 °C and allowed to warm to r.t. The aqueous phase was extracted three times with 10 mL Et_2_O. The combined organic phases were dried over Na_2_SO_4_ and filtered. The solvents were removed under reduced pressure. The crude product was taken up in 4 mL 1 M potassium hydroxide solution and the resulting solution was stirred for 7 h at r.t. At 0 °C, the solution was acidified to pH 1.0 by addition of 6 M hydrochloric acid. The aqueous phase was extracted three times with 10 mL EtOAc. The combined organic phases were dried over Na_2_SO_4_ and filtered. The solvents were removed under reduced pressure. Purification by flash column chromatography (EtOAc/PE = 1:10) yielded 40% of compound ***rac***-**14h** as a pale-yellow oil (16 mg, 0.15 mmol).

#### 4.2.3. Analytical Data


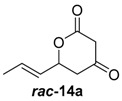


*(E)-6-(Prop-1-en-1-yl)dihydro-2H-pyran-2,4(3H)-dione* (***rac*-14a**): *R*_f_ (EtOAc/PE = 1:4): 0.1; ^1^H-NMR (400 MHz, CDCl_3_): δ_H_ = 5.95–5.86 (m, 1H, 7-C*H*), 5.61–5.55 (m, 1H, 6-C*H*), 5.13–5.08 (m, 1H, 5-C*H*), 3.54 (d, 1H, ^2^*J =* 19.2 Hz, 2-C*H*_2_), 3.44 (d, 1H, ^2^*J =* 19.1 Hz, 2-C*H*_2_), 2,76 (dd, 1H, ^2^*J =* 18.2 Hz, ^3^*J =* 3.6 Hz, 4-C*H*_2_), 2,62 (dd, 1H, ^2^*J =* 18.1 Hz, ^3^*J =* 9.5 Hz, 4-C*H*_2_), 1.77 (m, 3H, 8-C*H*_3_); ^13^C-NMR (100 MHz, CDCl_3_): δ_C_ = 17.92 (8-*C*H_3_), 43.57 (4-*C*H_2_), 47.12 (2-*C*H_2_), 75.64 (5-*C*H), 126.67 (6-*C*H), 132.03 (7-*C*H), 167.14 (1-*C*), 199.96 (3-*C*); HRMS (ESI): *m/z*: calc. for C_8_H_10_NaO_3_ [M + Na]^+^ 177.0528, found 177.0529 [M + Na]^+^.


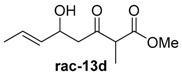


*Methyl-(E)-5-hydroxy-2-methyl-3-oxooct-6-enoate* (***rac*-13d**): ^1^H-NMR (400 MHz, CDCl_3_): δ_H_ = 5.77–5.68 (m, 1H, 8-C*H*), 5.52–5.45 (m, 1H, 7-C*H*), 4.57–4.50 (m, 1H, 6-C*H*), 3.73 (s, 3H, 10-C*H*_3_), 3.55 (q, 1H, ^3^*J* = 7.1 Hz, 2-C*H*), 2.83–2.72 (m, 2H, 5-C*H*_2_), 2.70–2.68 (m, 1H, O*H*), 1.70–1.68 (m, 3H, 9-C*H*_3_), 1.35 (dd, 3H, ^3^*J* = 7.1 Hz, ^5^*J* = 2.4 Hz, 3-C*H*_3_); ^13^C-NMR (100 MHz, CDCl_3_): δ_C_ = 206.2, 206.1 (4-*C*), 170.8, 170.7 (1-*C*), 131.9, 131.9 (8-*C*H), 127.6, 127.6 (7-*C*H), 68.7, 68.6 (6-*C*H), 53.5, 53.4 (10-*C*H_3_), 52.7, 52.7 (2-*C*H), 48.3, 48.1 (5-*C*H_2_), 17.8 (9-*C*H_3_), 12.7, 12.7 (3-*C*H_3_). HRMS (ESI): *m/z*: calc. for C_10_H_16_O_4_Na [M + Na]^+^ 223.0945, found 223.0946 [M + Na]^+^.


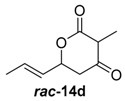


*(E)-3-Methyl-6-(prop-1-en-1-yl)dihydro-2H-pyran-2,4(3H)-dione* (***rac*-14d**): ^1^H-NMR (400 MHz, CDCl_3_): δ_H_ = 5.98–5.89 (m, 1H, 7-C*H*), 5.60–5.54 (m, 1H, 6-C*H*), 5.16–5.11 (m, 1H, 5-C*H*), 3.58 (q, 1H, ^3^*J* = 6.6 Hz, 2-C*H*), 2.76 (dd, 1H, ^2^*J* = 19.0 Hz, ^3^*J* = 3 Hz, 4-C*H*), 2.55 (dd, 1H, ^2^*J* = 19.0 Hz, ^3^*J* = 11.6 Hz, 4-C*H*), 1.78 (dd, 3H, ^3^*J* = 6.5 Hz, ^4^*J* = 1.6 Hz, 8-C*H*_3_), 1.37 (d, 3H, ^3^*J* = 6.5 Hz, 9-C*H*_3_); ^13^C-NMR (100 MHz, CDCl_3_): δ_C_ = 201.4 (3-*C*), 169.7 (1-*C*), 132.2 (7-*C*H), 126.6 (6-*C*H), 74.8 (5-*C*H), 51.9 (2-*C*H), 43.5 (4-*C*H_2_), 17.9 (8-*C*H_3_), 8.0 (9-*C*H_3_); HRMS (ESI): *m/z*: calc. for C_9_H_12_NaO_3_ [M + Na]^+^ 191.0684, found 191.0685 [M + Na]^+^.


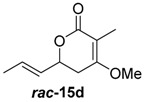


*(E)-4-Methoxy-3-methyl-6-(prop-1-en-1-yl)-5,6-dihydro-2H-pyran-2-one* (***rac*-15d**): ^1^H-NMR (200 MHz, CDCl_3_): δ_H_ = 5.96–5.71 (m, 1H, 8-C*H*), 5.68–5.54 (m, 1H, 7-C*H*), 4.80–4.68 (m, 1H, 6-C*H*), 3.78 (s, 3H, 10-C*H*_3_), 2.62–2.53 (m, 2H, 5-C*H*_2_), 1.79–1.77 (m, 3H, 3-C*H*_3_), 1.76–1.72 (m, 3H, 9-C*H*_3_).


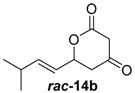


*(E)-6-(3-Methylbut-1-en-1-yl)dihydro-2H-pyran-2,4(3H)-dione* (***rac*-14b**): *R*_f_ (PE/EtOAc = 2:1): 0.20; ^1^H-NMR (400 MHz, CDCl_3_): δ = 5.86 (ddd, *J* = 15.6, 6.6, 1.2 Hz, 1H, CHC*H*CH(CH_3_)_2_), 5.50 (ddd, *J* = 15.6, 6.2, 1.4 Hz, 1H, C*H*CHCH(CH_3_)_2_), 5.11 (ddd, *J* = 9.7, 6.2, 3.6 Hz, 1H, CH_2_C*H*), 3.49 (q, *J* = 19.1 Hz, 2H, COC*H*CO), 2.77 (dd, *J* = 18.2, 3.6 Hz, 1H, C*H*_2_), 2.63 (dd, *J* = 18.2, 9.5 Hz, 1H, C*H*_2_), 2.42–2.29 (m, 1H, C*H*(CH_3_)_2_), 1.02 (dd, *J* = 6.8, 1.4 Hz, 6H, CH(C*H*_3_)_2_); ^13^C-NMR (100 MHz, CDCl3): δ = 199.9 (q, CHCH_2_*C*O), 167.2 (q, O*C*O), 143.7 (t, CH*C*HCH(CH_3_)_2_), 122.6 (t, *C*HCHCH(CH_3_)_2_), 75.8 (t, CH_2_*C*H), 47.1 (s, CO*C*H_2_CO), 43.7 (s, CH*C*H_2_), 31.0 (t, *C*H(CH_3_)_2_), 22.0 (p, C*H*_3_), 21.9 (p, C*H*_3_); HRMS (ESI): *m/z* for C_10_H_15_O*_3_* [M + Na]^+^: calculated 183.1024, observed: 183.1021.


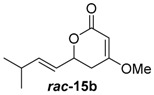


*(E)-4-Methoxy-6-(3-methylbut-1-en-1-yl)-5,6-dihydro-2H-pyran-2-one* (***rac*-15b**): *R*_f_ (PE/EtOAc = 2:1): 0.32; ^1^H-NMR (400 MHz, CDCl_3_): δ = 5.81 (ddd, *J* = 15.5, 6.5, 1.0 Hz, 1H, CHC*H*CH(CH_3_)_2_), 5.51 (ddd, *J* = 15.5, 6.7, 1.3 Hz, 1H, C*H*CHCH(CH_3_)_2_), 5.15 (d, *J* = 1.4 Hz, 1H, C*H*COCH_3_), 4.81 (ddd, *J* = 11.0, 6.7, 4.2 Hz, 1H, CH_2_CHO), 3.74 (s, 3H, OC*H*_3_), 2.61–2.50 (m, 1H, C*H*_2_), 2.40 (dd, *J* = 17.1, 4.2 Hz, 1H, C*H*_2_), 2.37–2.25 (m, 1H, C*H*(CH_3_)_2_), 1.01 (d, *J* = 1.3 Hz, 3H, CH(C*H*_3_)_2_), 0.99 (d, *J* = 1.3 Hz, 3H, CH(C*H*_3_)_2_); ^13^C-NMR (100 MHz, CDCl_3_): δ = 172.7 (q, *C*OCH_3_), 167.2 (q, O*C*O), 142.6 (t, CH*C*HCH(CH_3_)_2_), 123.8 (t, *C*HCHCH(CH_3_)_2_), 90.6 (t, *C*HCOCH_3_), 76.5 (t, CH_2_*C*HO), 56.2 (p, O*C*H_3_), 33.5 (s, CH_2_), 30.9 (t, *C*H(CH_3_)_2_), 22.0 (p, CH(*C*H_3_)_2_), 22.0 (p, CH(*C*H_3_)_2_); HRMS (ESI): *m/z* for C11H_16_O_3_ [M + Na]^+^: calculated 219.0997, observed: 219.0997.


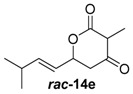


*(E)-3-Methyl-6-(3-methylbut-1-en-1-yl)dihydro-2H-pyran-2,4(3H)-dione* (***rac*-14e**): *R*_f_ (PE/EtOAc = 2:1): 0.24; ^1^H-NMR (400 MHz, CDCl3): δ = 5.97 (ddd, *J* = 15.5, 6.5, 0.9 Hz, 1H, CHCHCH(CH_3_)_2_), 5.58 (ddd, *J* = 15.5, 6.8, 1.4 Hz, 1H, CHCHCH(CH_3_)_2_), 5.31–5.14 (m, 1H, CH_2_CH), 3.65 (q, *J* = 6.7 Hz, 1H, COCHCH_3_), 2.85 (dd, *J* = 19.0, 3.1 Hz, 1H, CH_2_), 2.64 (dd, *J* = 19.0, 11.6 Hz, 1H, CH_2_), 2.52–2.35 (m, 1H, CH(CH_3_)_2_), 1.45 (d, *J* = 6.6 Hz, 3H, COCHCH_3_), 1.11 (d, *J* = 1.6 Hz, 3H, CH(CH_3_)_2_), 1.10 (d, *J* = 1.5 Hz, 3H, CH(CH_3_)_2_); ^13^C-NMR (100 MHz, CDCl_3_): δ = 201.3 (q, CH_2_COCH), 169.8 (q, OCO), 143.8 (t, CHCHCH(CH_3_)_2_), 122.5 (t, CHCHCH(CH_3_)_2_), 74.9 (t, CH_2_CH), 51.9 (t, COCHCH_3_), 43.6 (s, CH_2_), 30.9 (p, COCHCH_3_), 22.0 (p, CH(CH_3_)_2_), 21.9 (p, CH(CH_3_)_2_), 8.0 (p, COCHCH_3_); HRMS (ESI): *m*/*z* for C_11_H_16_O_3_ [M + Na]^+^: calculated 219.0999, observed: 219.0997.


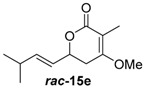


*(E)-4-Methoxy-3-methyl-6-(3-methylbut-1-en-1-yl)-5,6-dihydro-2H-pyran-2-one* (***rac*-15e**): *R*_f_ (PE/EtOAc = 2:1): 0.78; ^1^H-NMR (400 MHz, CDCl_3_): δ = 5.82 (ddd, *J* = 15.5, 6.5, 1.0 Hz, 1H, CHC*H*CH(CH_3_)_2_), 5.54 (ddd, *J* = 15.5, 6.9, 1.4 Hz, 1H, C*H*CHCH(CH_3_)_2_), 4.84–4.67 (m, 1H, CH_2_C*H*), 3.79 (s, 3H, OC*H*_3_), 2.75–2.47 (m, 2H, C*H*_2_), 2.45–2.23 (m, 1H, C*H*(CH_3_)_2_), 1.78 (dd, *J* = 1.8, 1.2 Hz, 3H, CCH_3_), 1.02 (d, *J* = 0.7 Hz, 3H, CH(C*H*_3_)_2_), 1.00 (d, *J* = 0.7 Hz, 3H, CH(C*H*_3_)_2_); ^13^C-NMR (100 MHz, CDCl_3_): δ *=* 168.5 (q, *C*OCH_3_), 165.2 (q, O*C*O), 142.6 (t, CH*C*HCH(CH_3_)_2_), 124.1 (t, *C*HCHCH(CH_3_)_2_), 103.8 (q, *C*CH_3_), 75.4 (t, O*C*H), 55.6 (p, O*C*H_3_), 30.9 (s, *C*H_2_), 29.9 (t, *C*H(CH_3_)_2_), 22.1 (p, CH(*C*H_3_)_2_), 22.1 (p, CH(*C*H_3_)_2_), 9.0 (p, C*C*H_3_); HRMS (ESI): *m/z* for C_12_H_19_O_3_ [M + Na]^+^: calculated 211.1330, observed: 211.1334.


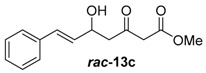


*Methyl-(E)-5-hydroxy-3-oxo-7-phenylhept-6-enoate* (***rac*-13c**): ^1^H-NMR (400 MHz, CDCl_3_): δ = 7.44–7.21 (m, 5H, *H*_aromat._), 6.68 (dd, *J* = 15.9, 1.0 Hz, 1H, *H*_olefin._), 6.23 (dd, *J* = 15.9, 6.2 Hz, 1H, *H*_olefin._), 4.87–4.74 (m, 1H, C*H*OH), 3.77 (s, 3H, OC*H*_3_), 3.55 (s, 2H, COC*H*_2_CO), 2.90 (d, *J* = 6.0 Hz, 2H, CHOHC*H*_2_CO). The analytical data was consistent with the literature [30].


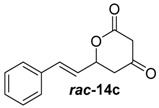


*(E)-6-Styryldihydro-2H-pyran-2,4(3H)-dione* (***rac*-14c**): ^1^H-NMR (400 MHz, CDCl_3_): δ = 7.47–7.29 (m, 5H, *H*_aromat._), 6.77 (dd, *J* = 16.0, 1.1 Hz, 1H, CC*H*CHCHO), 6.24 (dd, *J* = 16.0, 6.0 Hz, 1H, CCHC*H*CHO), 5.40–5.29 (m, 1H, C*H*CH_2_), 3.56 (q, *J* = 19.2 Hz, 2H, C*H*_2_COO), 2.89 (dd, *J* = 18.2, 3.6 Hz, 1H, CHC*H*_2_CO), 2.75 (dd, *J* = 18.2, 9.6 Hz, 1H, CHC*H*_2_CO). The analytical data was consistent with the literature [30].


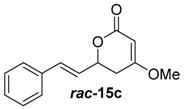


*(E)-4-Methoxy-6-styryl-5,6-dihydro-2H-pyran-2-one* (***rac*-15c**): *R*_f_ (PE/EtOAc = 2:1): 0.21; ^1^H-NMR (200 MHz, CDCl_3_): δ = 7.51–7.24 (m, 5H, *H*_aromat._), 6.78 (dd, *J* = 16.0, 1.0 Hz, 1H, PhC*H*CH), 6.30 (dd, *J* = 16.0, 6.2 Hz, 1H, PhCHC*H*), 5.24 (d, *J* = 1.1 Hz, 1H, OCC*H*), 5.11 (ddd, *J* = 11.2, 5.5, 1.2 Hz, 1H, CH_2_C*H*), 3.81 (s, 3H, OC*H*_3_), 2.87–2.47 (m, 2H, C*H*_2_); ^13^C-NMR (100 MHz, CDCl_3_): δ = 172.4 (q, *C*OCH_3_), 166.9 (q, O*C*O), 135.9 (t, *C*_aromat._), 133.3 (t, *C*_aromat._), 128.8 (t, *C*_aromat._), 128.5 (t, Ph*C*HCH), 126.884 (t, *C*_aromat._), 125.6 (t, PhCH*C*H), 90.7 (t, OC*C*H), 76.0 (t, O*C*H), 56.3 (p, O*C*H_3_), 33.5 (s, *C*H_2_); HRMS (ESI): *m/z* for C_14_H_15_O_3_ [M + Na]^+^: calculated 231.1021, observed: 231.1018.


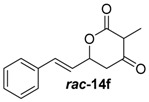


*(E)-3-Methyl-6-styryldihydro-2H-pyran-2,4-(3H)-dione* (***rac*-14f**): *R*_f_ (PE/EtOAc = 1:1): 0.44; ^1^H-NMR (400 MHz, CDCl_3_) δ = 7.58–7.28 (m, 5H, *H*_aromat._), 6.80 (d, *J* = 15.9 Hz, 1H, PhC*H*), 6.23 (dd, *J* = 15.9, 6.5 Hz, 1H, CCHC*H*), 5.49–5.20 (m, 1H, CH_2_C*H*), 3.65 (q, *J* = 6.6 Hz, 1H, COC*H*), 3.09–2.82 (m, 1H, C*H*_2_), 2.79–2.52 (m, 1H, C*H*_2_), 1.41 (d, *J* = 6.6 Hz, 3H, C*H*_3_); ^13^C-NMR (100 MHz, CDCl_3_): δ = 200.9 (q, *C*OCH_2_), 269.5 (q, O*C*O), 135.4 (1C, PhC*H*), 134.4 (1C, PhCHC*H*), 129.0 (1C, *C*_aromat._), 128.9 (2C, *C*_aromat._), 127.0 (2C, *C*_aromat._), 123.8 (1C, *C*_aromat._), 74.6 (1C, CH_2_*C*H), 52.0 (1C, *C*H_2_CO*C*HCO), 43.6 (1C, *C*H_2_), 8.1 (1C, *C*H_3_); HRMS (ESI): *m/z* for C_14_H_14_O_3_ [M + Na]^+^: calculated 253.0840, observed: 253.0841.


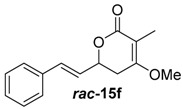


*(E)-4-Methoxy-3-methyl-6-styryl-5,6-dihydro-2H-pyran-2-one* (***rac*-15f**) *R*_f_ (PE/EtOAc = 2:1): 0.44; ^1^H-NMR (400 MHz, CDCl_3_): δ = 7.45–7.28 (m, 5H, *H*_aromat._), 6.75 (d, *J* = 16.0 Hz, 1H, PhC*H*CH), 6.29 (dd, *J* = 16.0, 6.4 Hz, 1H, PhCHC*H*), 5.04–4.91 (m, 1H), 3.81 (s, 3H, OC*H*_3_), 2.81–2.58 (m, 2H, C*H*_2_), 1.82 (dd, *J* = 1.9, 1.2 Hz, 3H, CC*H*_3_); ^13^C-NMR (100 MHz, CDCl_3_): δ = 165.0 (q, CH_2_*C*O), 135.9 (q, O*C*O), 133.3 (t, Ph*C*HCH), 128.9 (t, *C*_aromat._), 128.5 (t, PhCH*C*H), 127.2 (t, OCHCHCH*C*H), 126.8 (t, *C*_aromat._), 125.9 (t, *C*_aromat._), 104.0 (p, *C*CH_3_), 74.9 (t, O*C*H), 55.7 (p, O*C*H_3_), 29.9 (s, *C*H_2_), 9.1 (p, C*C*H_3_); HRMS (ESI): *m/z* for C_15_H_16_O_3_ [M + Na]^+^: calculated 267.0996, observed: 267.0997.


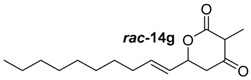


*(E)-6-(Dec-1-en-1-yl)-3-methyldihydro-2H-pyran-2,4(3H)-dion* (***rac*-14g**): *R*_f_ (PE/EtOAc = 20:1): 0.11; ^1^H-NMR (400 MHz, CDCl_3_): δ = 6.04–5.83 (m, 1H, CH_2_C*H*CHCHO), 5.54 (ddt, *J* = 15.4, 6.9, 1.4 Hz, 1H, CH_2_CHC*H*CHO), 5.31–5.04 (m, 1H, CH_2_C*H*O), 3.57 (q, *J* = 6.6 Hz, 1H, C*H*_3_CH), 2.77 (dd, *J* = 19.0, 3.0 Hz, 1H, C*H*_2_), 2.56 (dd, *J* = 19.0, 11.6 Hz, 1H, C*H*_2_), 2.13–2.01 (m, 2H, CHCHC*H*_2_), 1.37 (d, *J* = 6.6 Hz, 3H, CHC*H*_3_), 1.25 (t, *J* = 11.0 Hz, 12H, C*H*_2_C*H*_2_C*H*_2_C*H*_2_C*H*_2_C*H*_2_), 0.88 (t, *J* = 6.8 Hz, 3H, CH_2_C*H*_3_); ^13^C-NMR (100 MHz, CDCl_3_): δ = 201.4 (q, CH_3_CH*C*O), 169.7 (q, O*C*O), 137.3 (t, CH_2_*C*HCHCHO), 125.2 (t, CH_2_CH*C*HCHO), 74.8 (t, CH*C*HO), 51.9 (t, CH_3_*C*H), 43.6 (s, CO*C*H_2_), 32.3 (s, CHCH*C*H_2_), 32.0 (s, CHCH_2_*C*H_2_), 29.5 (s, *C*H_2_), 29.4 (s, *C*H_2_), 29.3 (s, *C*H_2_), 28.8 (s, *C*H_2_), 22.8 (s, *C*H_2_), 14.3 (p, CH*C*H_3_), 8.0 (p, CH_2_*C*H_3_); HRMS (ESI): *m/z* for C_16_H_26_O_3_ [M + Na]^+^: calculated 289.1781, observed: 289.1780.


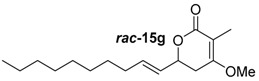


*(E)-6-(Dec-1-en-1-yl)-4-methoxy-3-methyl-5,6-dihydro-2H-pyran-2-on* (***rac*-15g**): *R*_f_ (PE/EtOAc = 2:1): 0.53; ^1^H-NMR (400 MHz, CDCl_3_): δ = 5.84 (ddd, *J* = 7.6, 7.2, 3.8 Hz, 1H, CH_2_C*H*CHCHO), 5.58 (ddt, *J* = 15.4, 6.8, 1.4 Hz, 1H, CH_2_CHC*H*CHO), 4.80–4.64 (m, 1H, CCH_2_C*H*O), 3.78 (s, 3H, OC*H*_3_), 2.68–2.48 (m, 2H, C*H*_2_COCH_3_), 2.06 (dd, *J* = 13.9, 7.0 Hz, 2H, CHCHCHC*H*_2_), 1.78 (d, *J* = 0.6 Hz, 3H, CC*H*_3_), 1.33–1.18 (m, 12H, C*H*_2_C*H*_2_C*H*_2_C*H*_2_C*H*_2_C*H*_2_), 0.88 (t, *J* = 6.8 Hz, 3H, CH_2_C*H*_3_); ^13^C-NMR (100 MHz, CDCl_3_): δ = 168.5 (q, *C*OCH_3_), 165.1 (q, O*C*O), 136.0 (t, CH_2_*C*HCHCHO), 126.9 (t, CH_2_CH*C*HCHO), 103.8 (q, *C*CH_3_), 75.3 (t, CH*C*HO), 55.6 (p, O*C*H_3_), 32.3 (s, CHO*C*H_2_), 32.0 (s, CHCH*C*H_2_), 29.9 (s, *C*H_2_*C*H_2_*C*H_2_*C*H_2_*C*H_2_), 29.6 (s, *C*H_2_*C*H_2_*C*H_2_*C*H_2_*C*H_2_), 29.4 (s, *C*H_2_*C*H_2_*C*H_2_*C*H_2_*C*H_2_), 29.3 (s, *C*H_2_*C*H_2_*C*H_2_*C*H_2_*C*H_2_), 29.0 (s, *C*H_2_*C*H_2_*C*H_2_*C*H_2_*C*H_2_), 22.8 (s, *C*H_2_*C*H_2_*C*H_2_*C*H_2_*C*H_2_), 14.3 (p, C*C*H_3_), 9.0 (p, CH_2_*C*H_3_); HRMS (ESI): *m/z* for C_17_H_28_O_3_ [M + Na]^+^: calculated 303.1936, observed: 303.1936.


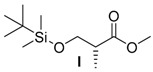


*Methyl-(R)-3-((tert-Butyldimethylsilyl)oxy)-2-methylpropanoate* (**I**): *R*_f_ = 0.9 (petroleum ether/Et_2_O 10:1); ${\left[\alpha \right]}_{D}^{23}$ = −0.1 (*c* = 0.86, CH_2_Cl_2_); ^1^H-NMR (400 MHz, CDCl_3_): δ_H_ = 3.78 (dd, 1H, ^2^*J* = 9.7 Hz, ^3^*J* = 6.9 Hz, 3-C*H*), 3.67 (s, 3H, 5-C*H*_3_), 3.64 (dd, 1H, ^2^*J* = 9.8 Hz, ^3^*J* = 6.0 Hz, 3-C*H*), 2.65 (sext, 1H, ^3^*J* = 6.8 Hz, 2-C*H*), 1.13 (d, 3H, ^3^*J* = 6.8 Hz, 4-C*H*_3_), 0.87 (s, 9H, 7-C*H*_3_), 0.03 (d, 6H, ^3^*J* = 1.4 Hz, 6-C*H*_3_); ^13^C-NMR (100 MHz, CDCl_3_): δ*_C_* = 175.6 (1-*C*), 65.4 (3-*C*H_2_), 51.6 (5-*C*H_3_), 42.7 (2-*C*H), 25.9 (7-*C*H_3_), 18.3 (8-*C*), 13.6 (4-*C*H_3_), −5.4 (6-*C*H_3_).


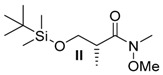


*(R)-3-((tert-Butyldimethylsilyl)oxy)-N-methoxy-N,2-dimethylpropanamide* (**II**): *R*_f_ (PE/EE 3.5:1): 0.50; ${\left[\alpha \right]}_{D}^{20}$: −15.8 (*c* = 1.1, CH_2_Cl_2_); ^1^H-NMR (400 MHz, CDCl_3_): δ = 3.83 (d, *J* = 9.6, 8.2 Hz, 1H, CH_2_-OTBS), 3.71 (s, 3H, OMe), 3.53 (d, *J* = 9.6, 6.2 Hz, 1H, CH_2_-OTBS), 3.19 (m, 4H, CH-CH_3_, N-Me), 1.07 (d, *J* = 6.8 Hz, 3H, CH_3_-CH_2_), 0.87 (m, 9H, OTBS, 0.04 (s, 3H, OTBS), 0.03 (s, 3H, OTBS) ppm; ^13^C-NMR (100 MHz, CDCl_3_): δ = 171.3 (q, CO-N), 65.7 (s, CH_2_-OTBS), 61.5 (p, OMe), 38.0 (t, CH-CH_3_), 31.6 (p, NMe), 25.9 (p, OTBS), 18.3 (q, OTBS), 13.8 (p, CH_3_-CH), −5.5 (p, OTBS) ppm; HRMS [ESI] *m/z* for C_12_H_27_NO_3_SiNa [M + Na]^+^: ber. 284.1658 gef. 284.1659.


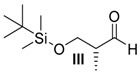


*(R)-3-((tert-Butyldimethylsilyl)oxy)-2-methylpropanal* (**III**): ${\left[\alpha \right]}_{D}^{23}$ = −0.1 (*c* = 0.68, CH_2_Cl_2_); ^1^H-NMR (200 MHz, CDCl_3_): δ_H_ = 9.74 (d, 1H, ^3^*J* = 1.6 Hz, 1-C*H*), 3.83 (m, 2H, 3-C*H*_2_), 2.61–2.44 (m, 1H, 2-C*H*), 1.09 (d, 3H, ^3^*J* = 7.0 Hz, 4-C*H*_3_), 0.87 (s, 9H, 6-C*H*_3_), 0.05 (s, 6H, 5-C*H*_3_); ^13^C-NMR (100 MHz, CDCl_3_): δ_C_ = 204.9 (1-*C*H), 65.0 (3-*C*H_2_), 42.0 (2-*C*H), 25.9 (6-*C*H_3_), 18.3 (7-*C*), 13.2 (4-*C*H_3_), −5.4 (5-*C*H_3_).


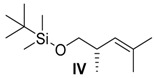


*(S)-tert-Butyl-((2,4-dimethylpent-3-en-1-yl)oxy)dimethylsilane* (**IV**): *R*_f_ = 0.5 (petroleum ether); ${\left[\alpha \right]}_{D}^{23}$ = +0.1 (*c* = 0.8, CH_2_Cl_2_); ^1^H-NMR (400 MHz, CDCl_3_): δ_H_ = 4.88–4.85 (m, 1H, 3-C*H*), 3.43 (dd, 1H, ^2^*J* = 9.7 Hz, ^3^*J* = 6.0 Hz, 1-C*H*_2_), 3.31 (dd, 1H, ^2^*J* = 9.8 Hz, ^3^*J* = 7.6 Hz, 1-C*H*_2_), 2.57–2.46 (m, 1H, 2-C*H*), 1.68 (d, 3H, ^4^*J* = 1.2 Hz, 5-C*H*_3_), 1.63 (d, 3H, ^4^*J* = 1.2 Hz, 5-C*H*_3_), 0.92 (d, 3H, ^3^*J* = 6.6 Hz, 6-C*H*_3_), 0.89 (s, 9H, 9-C*H*_3_), 0.03 (s, 6H, 7-C*H*_3_); ^13^C-NMR (100 MHz, CDCl_3_): δ_C_ = 132.0 (4-*C*), 127.8 (3-*C*H), 68.3 (1-*C*H_2_), 35.7 (2-*C*H), 26.1 (9-*C*H_3_), 18.5 (8-*C*), 18.2 (5-*C*H_3_), 17.6 (6-*C*H_3_), −5.1 (7-*C*H_3_).


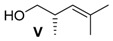


*(S)-2,4-Dimethylpent-3-en-1-ol* (**V**): *R*_f_ (PE:EE 5:1): 0.26; ^1^H-NMR (400 MHz, CDCl_3_): δ = 4.88 (dquin, *J* = 9.5, 1.4 Hz, 1H, CH=C), 3.51–3.43 (m, 1H, CH_2_-CH), 3.34–3.28 (m, 1H, CH_2_-CH), 2.66–2.54 (m, 1H, CH-CH_3_), 1.73 (d, *J =* 1.3 Hz, 3H, Me-C), 1.67 (d, *J* = 1.0 Hz, 3H, Me-C), 1.38 (bs, 1H, OH), 0.92 (d, *J* = 6.5 Hz, 3H, CH_3_-CH) ppm; ^13^C-NMR (100 MHz, CDCl_3_): δ = 134.3 (q, C=CH), 127.1 (t, CH=C), 67.9 (s, CH_2_-OH), 35.5 (t, CH-CH_3_), 25.9 (p, Me-C), 18.2 (p, CH_3_-CH), 17.0 (p, Me-C) ppm; ${\left[\alpha \right]}_{D}^{21}$: −39.4 (*c* = 1.1, CH_2_Cl_2_).


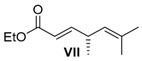


*(R,E)-Ethyl-4,6-dimethylhepta-2,5-dienoate* (**VII**): *R*_f_ (PE/EE 5:1): 0.65; ^1^H-NMR (400 MHz, CDCl_3_): δ = 6.87 (dd, *J* = 15.7, 6.5 Hz, 1H, CH=CH), 5.75 (dd, *J* = 15.7, 1.4 Hz, 1H, CH=CH), 4.94 (dquin, *J* = 8.7, 1.4 Hz, 1H, CH=C), 4.18 (q, *J* = 7.1 Hz, 2H, OEt), 3.19 (dddd, *J* = 15.3, 13.7, 6.8, 1.5 Hz, 1H, CH-CH_3_) 1.70 (d, *J* = 1.4 Hz, 3H, Me-C), 1.62 (d, *J* = 1.4 Hz, 3H, Me-C), 1.28 (t, *J* = 7.2 Hz, 3H, OEt), 1.10 (d, *J* = 7.2 Hz, 3H, CH_3_-CH) ppm; ^13^C-NMR (100 MHz, CDCl_3_): δ = 167.1 (q, CO_2_Et), 153.1 (t, CH=CH), 133.0 (q, C=CH), 126.4 (t, CH=C), 128.9 (t, CH=CH), 60.2 (s, OEt), 35.4 (CH-CH_3_), 25.8 (p, Me-C), 20.1 (p, CH_3_-CH), 18.0 (p, Me-C), 14.3 (p, OEt) ppm; HRMS [ESI] *m/z* for C_11_H_18_O_2_Na [M + Na]^+^: ber. 205.1204 gef. 205.1206; ${\left[\alpha \right]}_{D}^{22}$: +124.0 (*c* = 1.3, CH_2_Cl_2_).


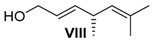


*(R,E)-4,6-Dimethylhepta-2,5-dien-1-ol* (**VIII**): *R*_f_ (PE/EE 5:1): 0.21; ^1^H-NMR (400 MHz, CDCl_3_): δ = 5.60 (ddt, *J* = 5.0, 3.8, 9.7 Hz, 2H, CH=CH), 4.95 (dquin, *J* = 9.0, 1.4 Hz, 1H, CH=C), 4.09 (m, 2H, CH_2_-OH), 3.11–3.01 (m, 1H, CH-CH_3_), 1.69 (d, *J* = 1.2 Hz, 3H, Me-C), 1.62 (d, *J* = 1.0 Hz, 3H, Me-C), 1.25 (bs, 1H, OH), 1.03 (d, *J =* 6.8 Hz, 3H, CH_3_-CH) ppm; ^13^C-NMR (100 MHz, CDCl3): δ = 137.7 (t, CH-CH_2_OH), 131.1 (q, C=CH), 128.4 (t, CH=CHCH_2_), 126.5 (t, CH=C), 63.9 (s, CH_2_-OH), 35.1 (CH-CH_3_), 25.7 (p, Me-C), 20.9 (p, CH_3_-CH), 17.9 (p, Me-C) ppm; ${\left[\alpha \right]}_{D}^{22}$: +74.7 (*c* = 1.0, CH_2_Cl_2_).


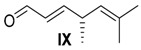


*(R,E)-4,6-Dimethylhepta-2,5-dienal* (**IX**): *R*_f_ (PE/EE 5:1): 0.58; ^1^H-NMR (400 MHz, CDCl_3_): δ = 9.51 (d, *J* = 7.8 Hz, 1H, H-CO), 6.73 (dd, *J =* 15.5, 6.3 Hz, 1H, CH=CHCO), 6.06 (ddd, *J* = 15.6, 7.9, 1.4 Hz, 1H, CH=CHCH), 4.96 (dquin., *J =* 8.8, 1.4 Hz, 1H, CH=C), 3.33 (dqd, *J =* 15.2, 6.8, 1.3 Hz, 1H, CH-CH_3_), 1.72 (d, *J =* 1.4 Hz, 3H, Me-C), 1.63 (d, *J* = 1.4 Hz, 3H, Me-C), 1.15 (d, *J* = 6.8 Hz, 3H, CH_3_-CH) ppm; ^13^C-NMR (100 MHz, CDCl_3_): δ = 194.5 (t, CO), 162.5 (t, CH=CHCO), 133.9 (t, CH-CO), 130.5 (q, C=CH), 125.6 (t, CH=C), 35.9 (CH-CH_3_), 25.7 (p, Me-C), 19.8 (p, CH_3_-CH), 18.0 (p, Me-C) ppm.


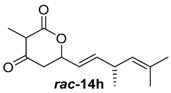


*((R,E)-3,5-Dimethylhexa-1,4-dien-1-yl)-3-methyldihydro-2H-pyran-2,4(3H)-dione* (***rac*-14h**): *R*_f_ (EtOAc/PE = 1:10): 0.3, ^1^H-NMR (500 MHz, CDCl_3_): δ_H_ = 5.83 (ddd, 1H, ^3^*J* = 9.3 Hz, ^3^*J* = 6.2 Hz, ^4^*J* = 0.9 Hz, 8-C*H*), 5.49 (ddt, 1H, ^3^*J* = 15.5 Hz, ^3^*J* = 6.8 Hz, ^4^*J* = 1.6 Hz, 7-C*H*), 5.17–5.12 (m, 1H, 6-C*H*), 4.93 (d, 1H, ^3^*J* = 9.0 Hz, 11-C*H*), 3.57 (q, 1H, ^3^*J* = 6.6 Hz, 2-C*H*), 3.11 (ddt, 1H, ^3^*J* = 7.0 Hz, ^3^*J* = 1.7 Hz, 9-C*H*), 2.77 (dd, 1H, ^2^*J* = 19.0 Hz, ^3^*J* = 2.7 Hz, 5-C*H*_2_), 2.56 (dd, 1H, ^2^*J* = 19.0 Hz, ^3^*J* = 11.7 Hz, 5-C*H*_2_), 1.70 (d, 3H, ^3^*J* = 1.1 Hz, 13-C*H*_3_), 1.62 (s, 3H, 13-C*H*_3_), 1.37 (d, 3H, ^3^*J* = 6.7 Hz, 3-C*H*_3_), 1.06 (dd, 3H, ^3^*J* = 6.9 Hz, ^4^*J* = 1.3 Hz, 10-C*H*_3_); ^13^C-NMR (125 MHz, CDCl_3_): δ_C_ = 201.3 (4-*C*), 169.8 (1-*C*), 141.3 (8-*C*H), 132.4 (12-*C*), 127.3 (11-*C*H), 122.9 (7-*C*H), 74.9 (6-*C*H), 51.9 (2-*C*H), 43.6 (5-*C*H_2_), 35.3 (9-*C*H), 25.9 (13-*C*H_3_), 20.7 (10-*C*H_3_), 18.9 (13-*C*H_3_), 8.0 (3-*C*H_3_). HRMS (ESI): *m/z*: calc. for C_14_H_20_NaO_3_ [M + Na]^+^ 259.1310, found 259.1312 [M + Na]^+^.

### 4.3. Cloning and Enzyme Expression

#### 4.3.1. Molecular Cloning

A codon-optimised gene for jerF was obtained as a plasmid jerF_pMK-T (Invitrogen, Waltham, MA, USA) provided with the NdeI (5′-CAT ATG-3′) and EcoRI (5′-GAA TTC-3′) restriction sites used for standard restriction cloning into pET-28a(+) (Novagen, Billerica, MA, USA) and pCOLD-I (Takara Bio USA Inc., Mountain View, CA, USA). For this purpose, jerF_pMK-T was double digested by NdeI (New England Biolabs, Ipswich, MA, USA) and EcoRI (New England Biolabs). The gel-purified insert was ligated into NdeI/EcoRI-treated pET-28a(+) and pCOLD-I respectively using T4 DNA Ligase (Thermo Fisher Scientific, Waltham, MA, USA) to generate recombinant plasmids jerF_pET-28a(+) and jerF pCOLD-I for expression of N-terminal His_6_-tagged fusion proteins.

For cloning into pET-20b(+) (Novagen), jerF was amplified by PCR under standard conditions using jerF_pMK-T as a template with primers as follows: forward, 5′-AGG CTC GAG TGC CGG ACT TTC GGT GC-3′; reverse, 5′-TGA GAT CTC ATA TGC GTA CCA GTG ATG C-3′. Gel-purified amplicons were double digested using XhoI (New England Biolabs) and NdeI, followed by the ligation into XhoI/NdeI-treated pET-20b(+) vector using T4 DNA Ligase to generate recombinant plasmid jerF_pET-20b(+) for expression of C-terminal His_6_-tagged fusion protein. All recombinant plasmids were transformed into E. coli TOP10 chemically competent cells for plasmid propagation.

#### 4.3.2. Protein Sequence

##### >JerF

MRTSDAVWAGAAGYTRARLQVYDFFIYGFNSPVAWKCPGEELLENYNRHVSGNHLDVGVGTGYLLDRCRFPTAKPRVFLMDLNPDALQVTAQRLHRFQPQTLRRNVLDPIRFDGEPFDSIGMNYLMHCVPGSIPEKAVMFDHLSALLKPGGVIFGSTVLSEGVDKGIVARAIMDRFNKKGIFSNTRDAASDLTRALEERFDDVSVRVVGCVGLFSARKRTCAGTESPA

#### 4.3.3. Enzyme Expression

Recombinant plasmids jerF_pET-28a(+), jerF_pET-20b(+) and jerF_pCOLD-I were used to transform E. coli BL21 (DE3) chemically competent cells. The resulting transformants were used to generate overnight cultures in LB broth under appropriate antibiotic selection (50 µg·mL^−1^ kanamycin for jerF_pET-28a(+), 50 µg mL^−1^ carbenicillin for the others). The next day, these cultures were used to inoculate 50 mL LB broth in 250-mL-flasks containing appropriate antibiotics to an initial OD_600_ of 0.05. The inoculated cultures were grown at 37 °C, 180 rpm for 2–3 h until OD_600_ reached 0.5–1.0. Isopropyl-β-d-thiogalactopyranoside (IPTG) was then added to a final concentration of 0.1 mM to induce gene expression. For jerF_pCOLD-I, the cells were cultured at 16 °C overnight. The cells were harvested at 10,000 g for 45 min at 4 °C. The cell pellet was resuspended in 10 mL/g reaction buffer (40 mM Tris HCl, 100 mM NaCl, pH 8.8) and applied to sonication. Cell debris was removed by centrifugation at 10,000 g for 40 min at 4 °C to obtain cell-free extract which were immediately used in enzyme assays.

### 4.4. Enzyme Assays

#### 4.4.1. Establishment of Assay Conditions

Initial enzyme assays with substrate **rac**-**14d** were carried out in 200 µL of reaction buffer (40 mM Tris HCl, 100 mM NaCl, 5 mM MgCl_2_, pH 8.8) containing JerF (2.7 mg/mL total protein), 0.25 mM **rac**-**14d** and 0.97 mM SAM tosylate as methyl donor, incubated at 28 °C for 16 h. The reaction was quenched by the addition of 100 µL brine followed by extraction with EtOAc (2 × 400 µL). The resulting organic extracts were dried and redissolved in 1 mL MeCN. LC-MS analysis were carried out with a Q-ToF Premier (Waters) in combination with a Waters Acquity Ultra performance LC system (H_2_O/MeCN = 95:5 + 0.1% FA → 5:95 + 0.1% FA, 0.4 mL/min, 8 min).

#### 4.4.2. Comparative Assaying of Synthetic Substrate Surrogates

Enzymatic substrate conversions were carried out in 200 µL of reaction buffer (25 mM HEPES, 100 mM NaCl, 5 mM MgCl_2_, pH 7.5) containing JerF (8.4 mg/mL total protein), 0.25 mM substrate surrogates and 4.18 mM SAM tosylate as methyl donor, incubated at 28 °C for 20 h. The reaction was quenched by the addition of 100 µL brine followed by extraction with EtOAc (2 × 200 µL). The resulting organic extracts were dried and redissolved in 200 µL MeCN. LC-MS analysis were carried out with a micromass LCT via loop-mode injection from a Waters Alliance 2695 HPLC system.

#### 4.4.3. Semi-preparative Scale Conversions

Semi-preparative scale JerF assays were carried out in a total volume of 10 mL, containing 4.3–4.6 mg of substrate and 3.6–4.0 mg/mL of total protein in reaction buffer (25 mM HEPES, 100 mM NaCl, 5 mM MgCl_2_, pH 7.5) and 1.67 mM SAM tosylate as methyl donor, incubated at 28 °C for 16 h. The reaction was quenched by the addition of 5 mL brine followed by extraction with EtOAc (3 mL × 15 mL). The resulting organic extracts were dried, redissolved in deuterated chloroform and subjected to ^1^H-NMR analysis and HPLC-MS analysis.

## Figures and Tables

**Table 1:** Conversions, yields and scale of the semi-preparative conversions with JerF.

Compound	Conversion ^a^	Crude yield	e.e. ^b^	Scale
***rac*-14c**	42%	n.d.	84%	4.6 mg
***rac*-14e**	27%	38%^c^	42%	4.6 mg
***rac*-14f**	35%	13%^c^	<10%	4.3 mg

^a^ The conversion was determined by integration of the signals of the protons at position 6 of the dione in the ^1^H-NMR spectra; ^b^ The e.e. was calculated from the peak areas of the chiral HPLC chromatograms. ^c^ Crude yield is given after column chromatography on silica gel. A non-removable, aliphatic impurity was co-purified (see Figures S45 and S47).

## Supplementary Materials

Supplementary materials can be accessed at: http://www.mdpi.com/1420-3049/21/11/1443/s1.
