# Supplementary material for: Characterisation of the Broadly-Specific O-Methyl-transferase JerF from the Late Stages of Jerangolid Biosynthesis
Source: Molecules. 2016 Oct 29;21(11):1443. doi: 10.3390/molecules21111443 (PMC6273487; doi:10.3390/molecules21111443)
Supplement: Supplementary file 1 [file molecules-21-01443-s001.pdf]

Supplementary Materials: Characterisation of the Broadly-Specific *O*-Methyltransferase JerF from the Late Stages of Jerangolid Biosynthesis

Steffen Friedrich, Franziska Hemmerling, Anna Warnke, Gesche Berkhan, Frederick Lindner and Frank Hahn

1. NMR Spectra

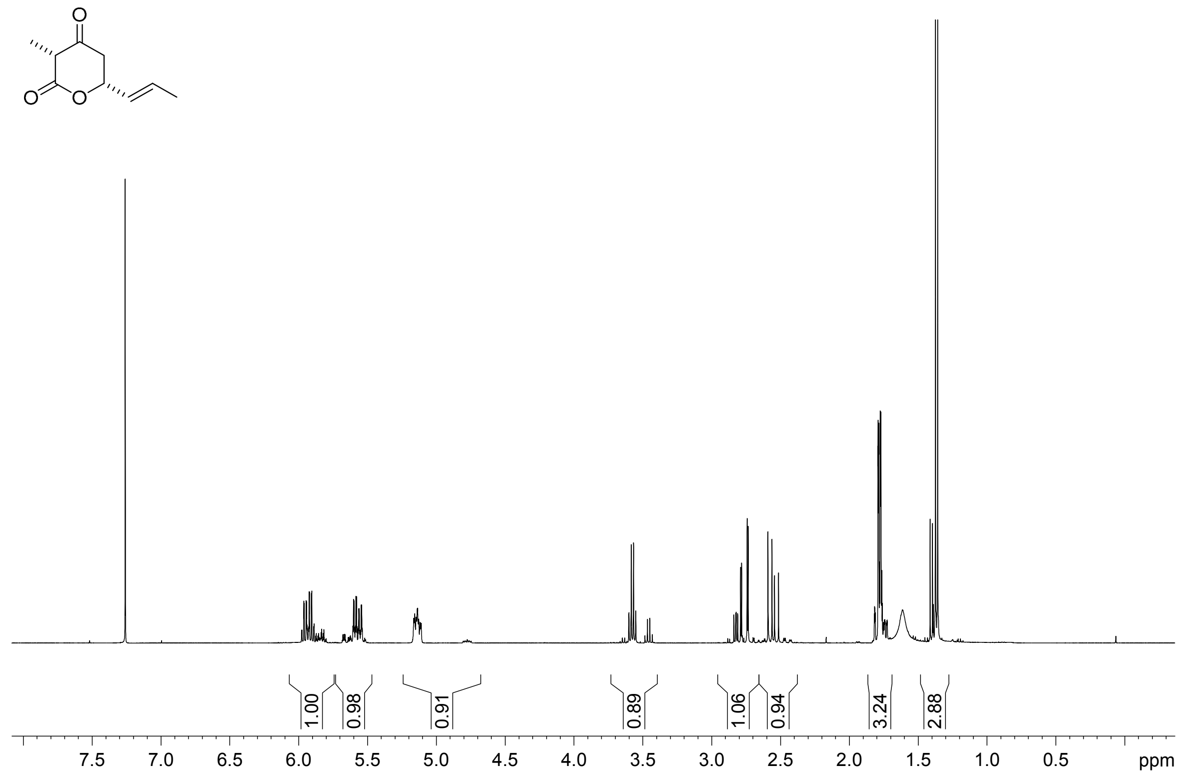

Figure S1. ^{1}H-NMR spectrum of compound ***rac*-14d**. The experiment was conducted at 400 MHz in $CDCl_3$.

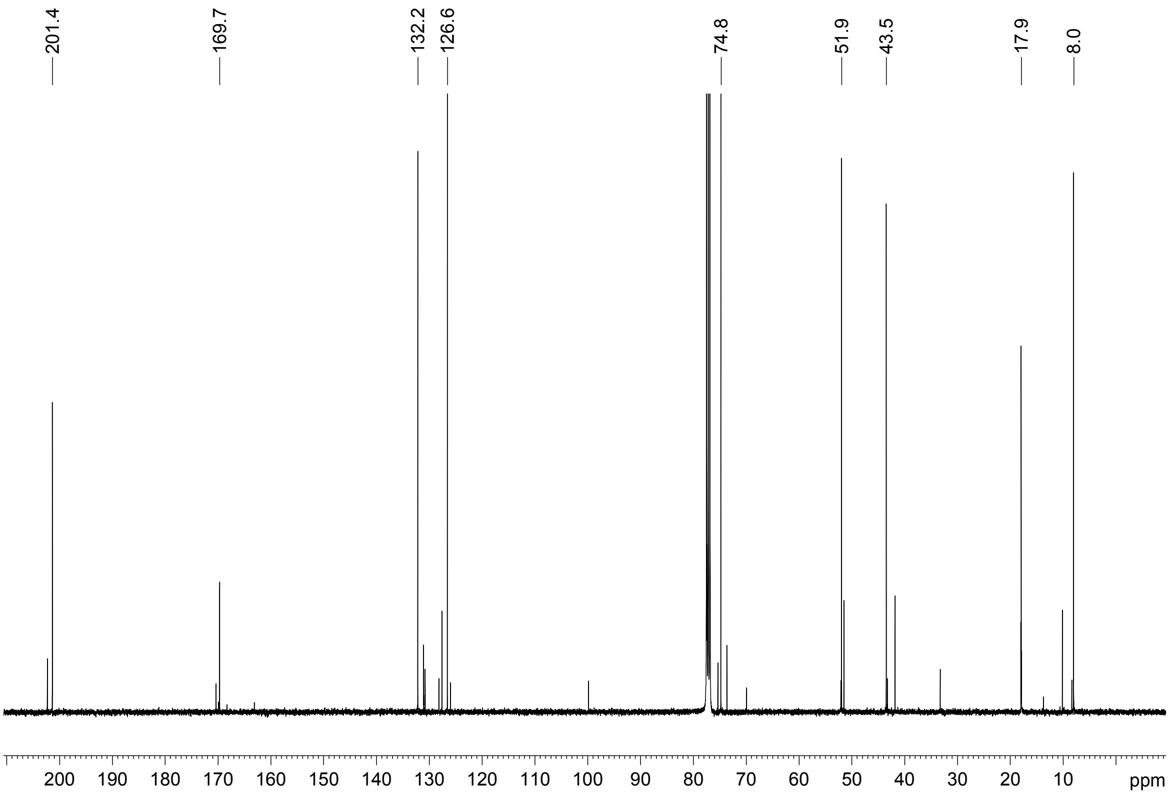

Figure S2. ^{13}C-NMR spectrum of compound ***rac*-14d**. The experiment was conducted at 100 MHz in $CDCl_3$.

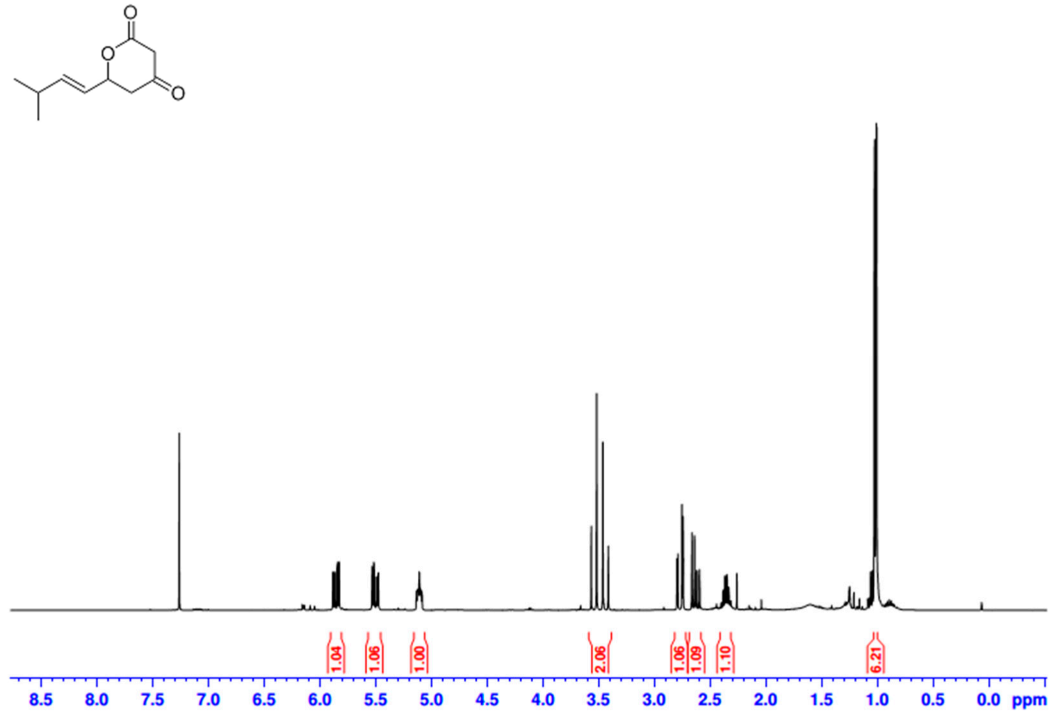

Figure S3. ^{1}H-NMR spectrum of compound ***rac*-14b**. The experiment was conducted at 400 MHz in $CDCl_3$.

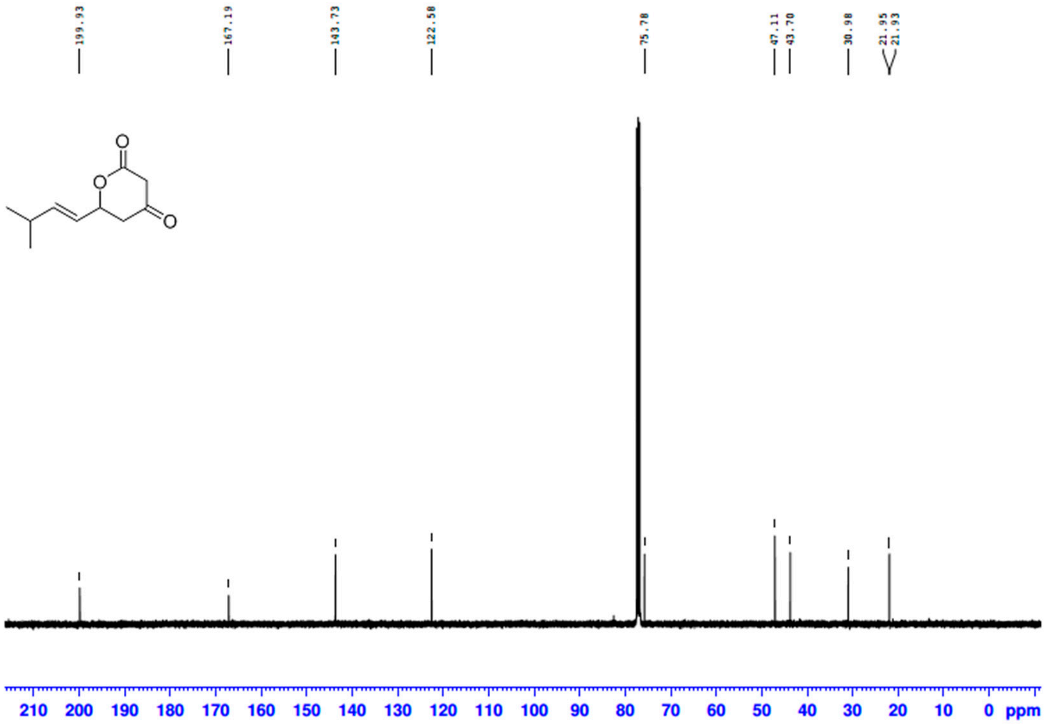

Figure S4. ^{13}C-NMR spectrum of compound ***rac*-14b**. The experiment was conducted at 100 MHz in $CDCl_3$.

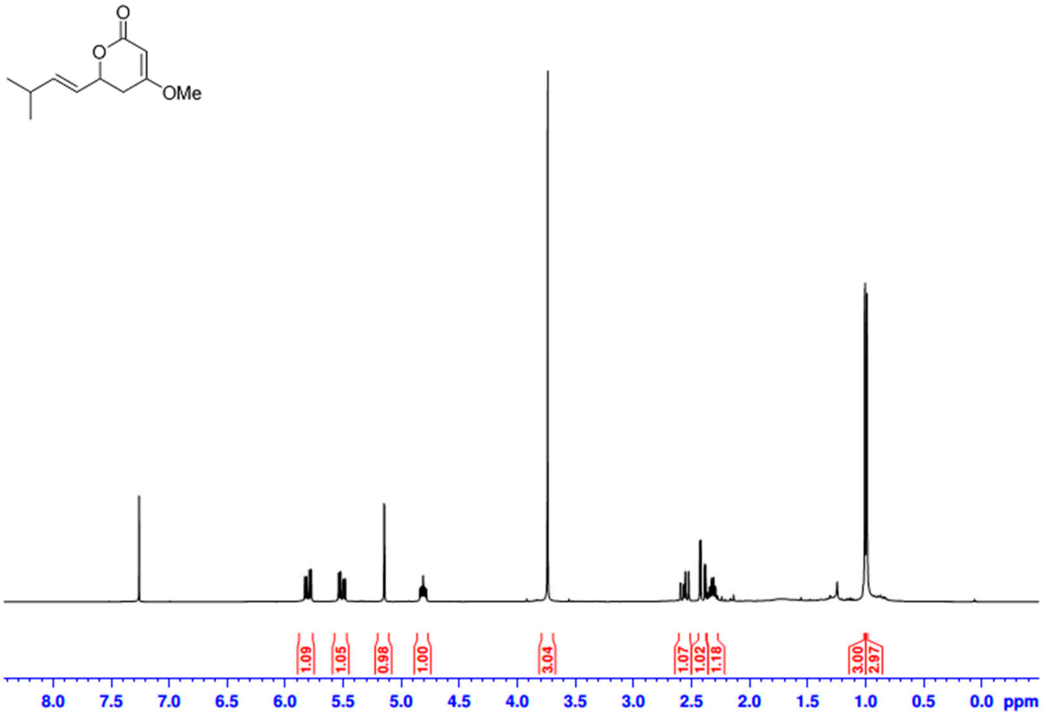

Figure S5. ^{1}H-NMR spectrum of compound ***rac*-15b**. The experiment was conducted at 400 MHz in $CDCl_3$.

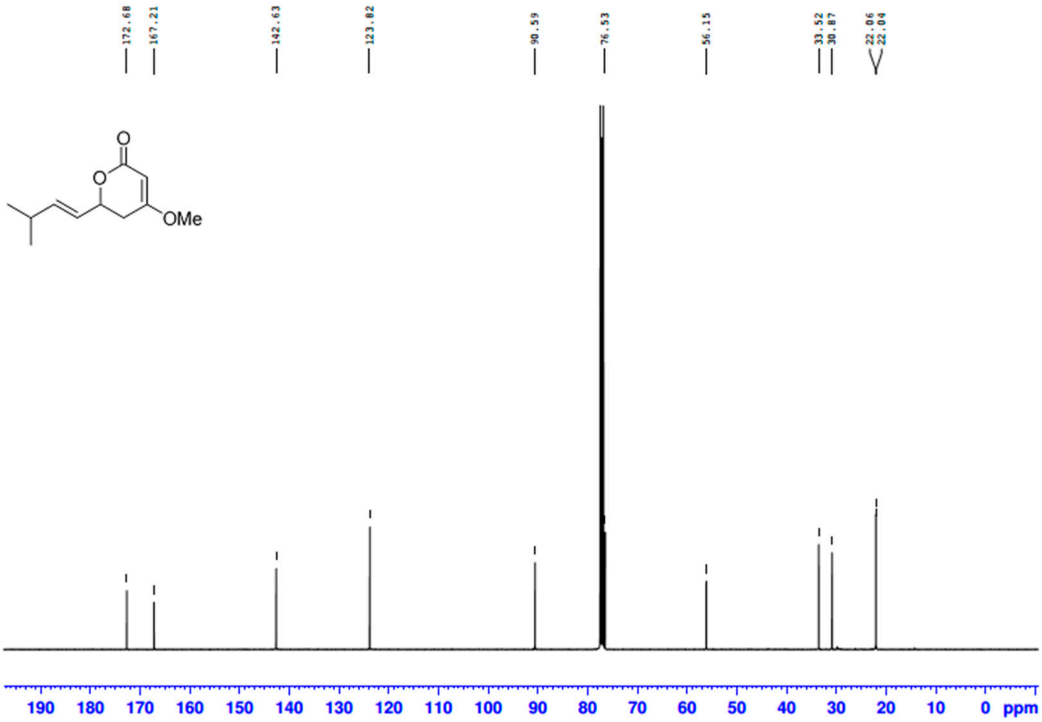

Figure S6. ^{13}C-NMR spectrum of compound ***rac*-15b**. The experiment was conducted at 100 MHz in $CDCl_3$.

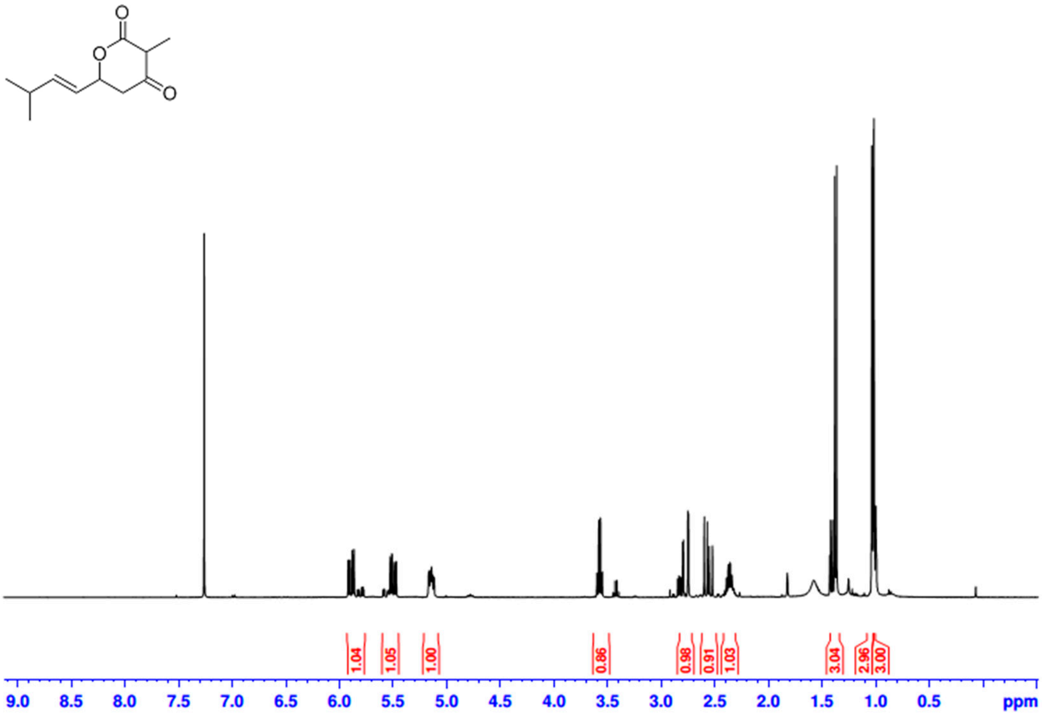

Figure S7. ^{1}H-NMR spectrum of compound ***rac*-14e**. The experiment was conducted at 400 MHz in $CDCl_3$.

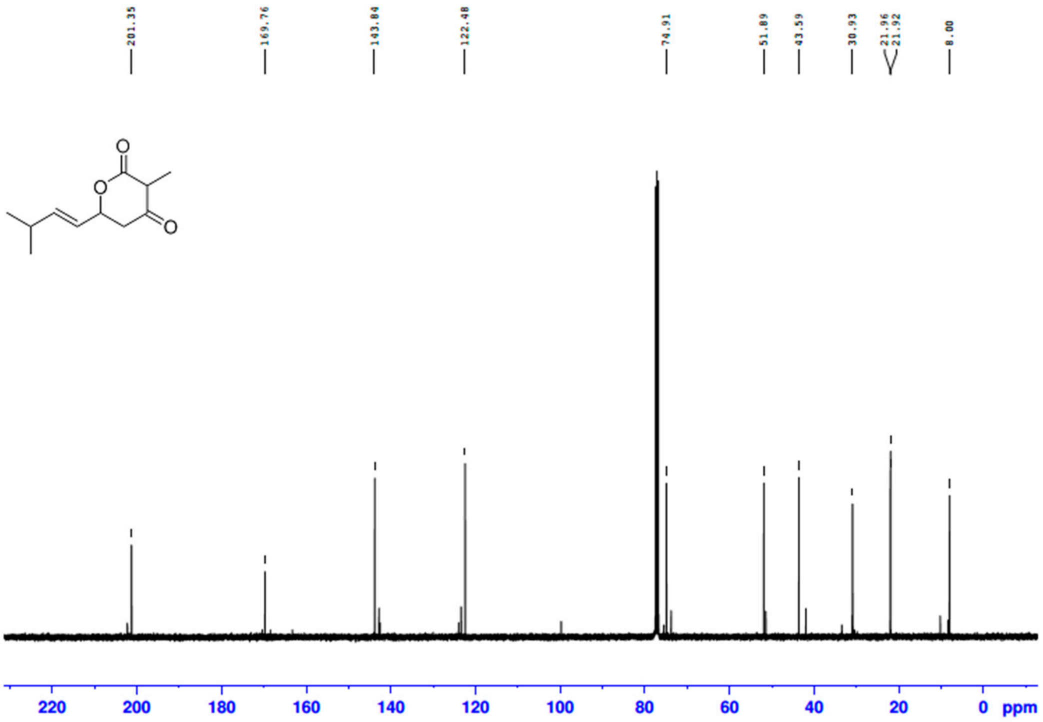

Figure S8. ^{13}C-NMR spectrum of compound ***rac*-14e**. The experiment was conducted at 100 MHz in $CDCl_3$.

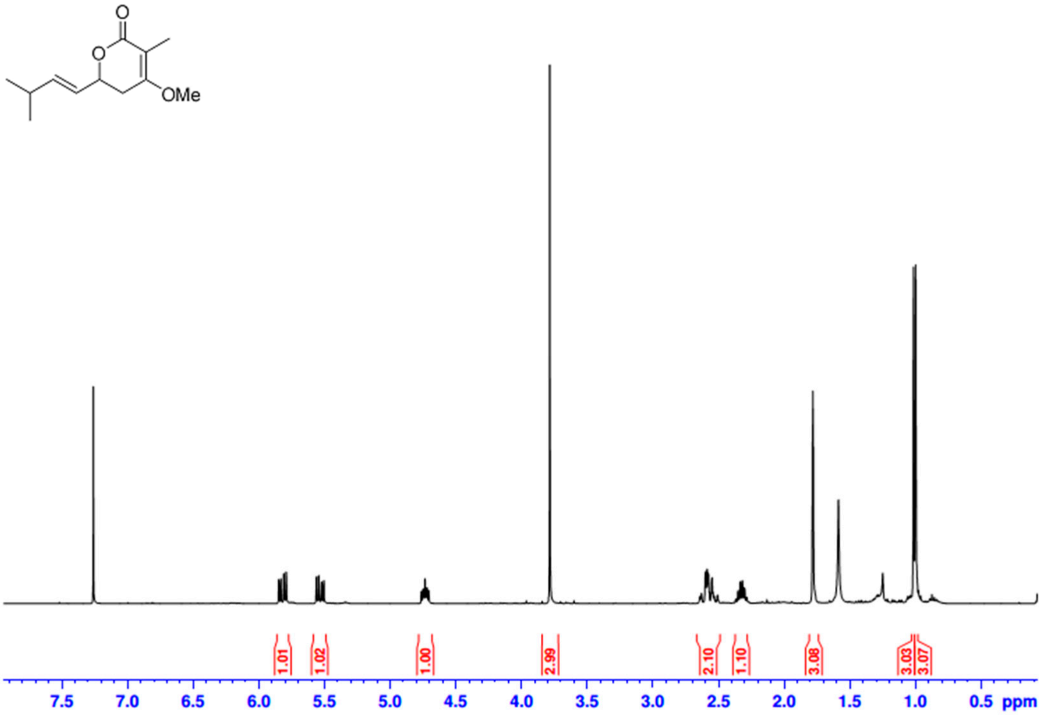

Figure S9. ^{1}H-NMR spectrum of compound ***rac*-15e**. The experiment was conducted at 400 MHz in $CDCl_3$.

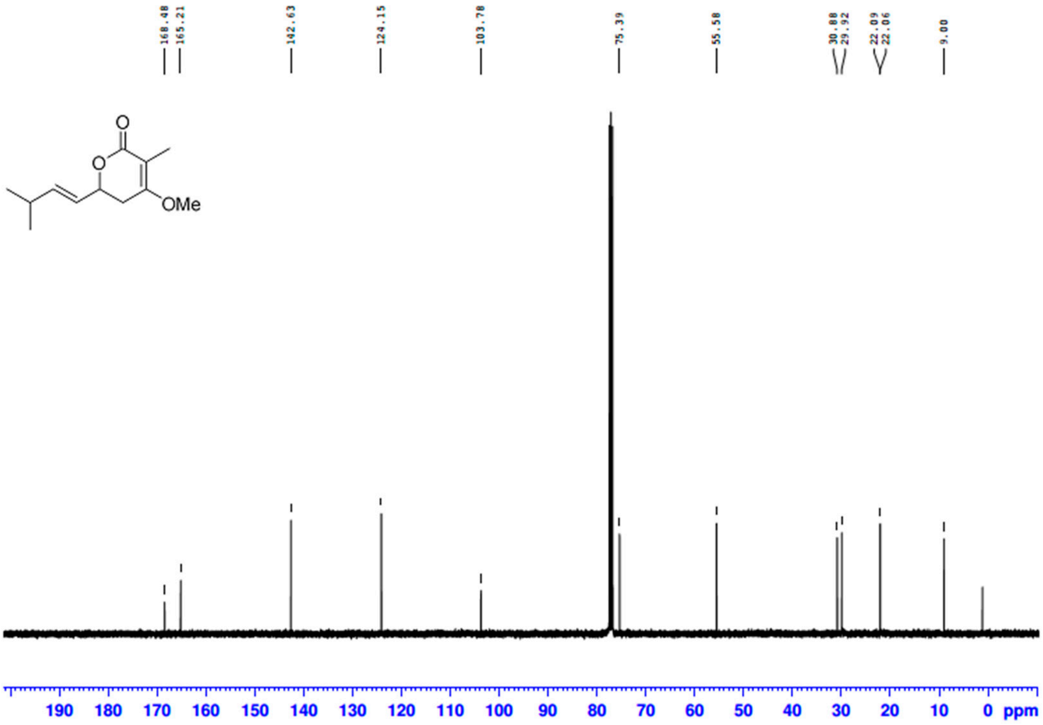

Figure S10. ^{13}C-NMR spectrum of compound ***rac*-15e**. The experiment was conducted at 100 MHz in $CDCl_3$.

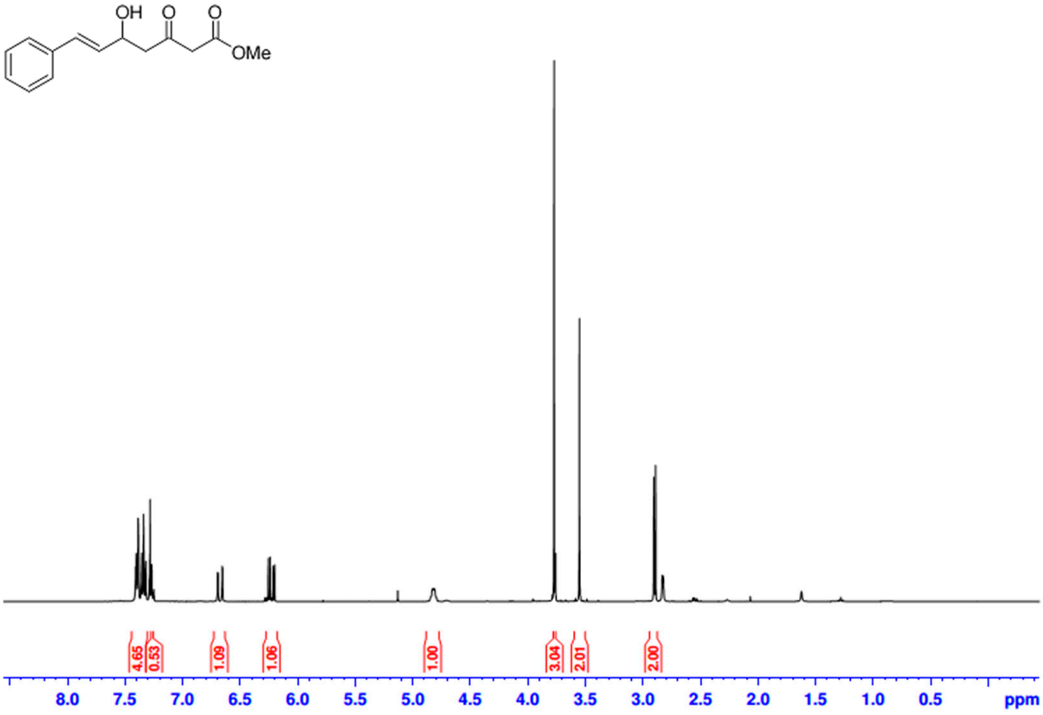

Figure S11. ^{1}H-NMR spectrum of compound ***rac*-13c**. The experiment was conducted at 400 MHz in $CDCl_3$.

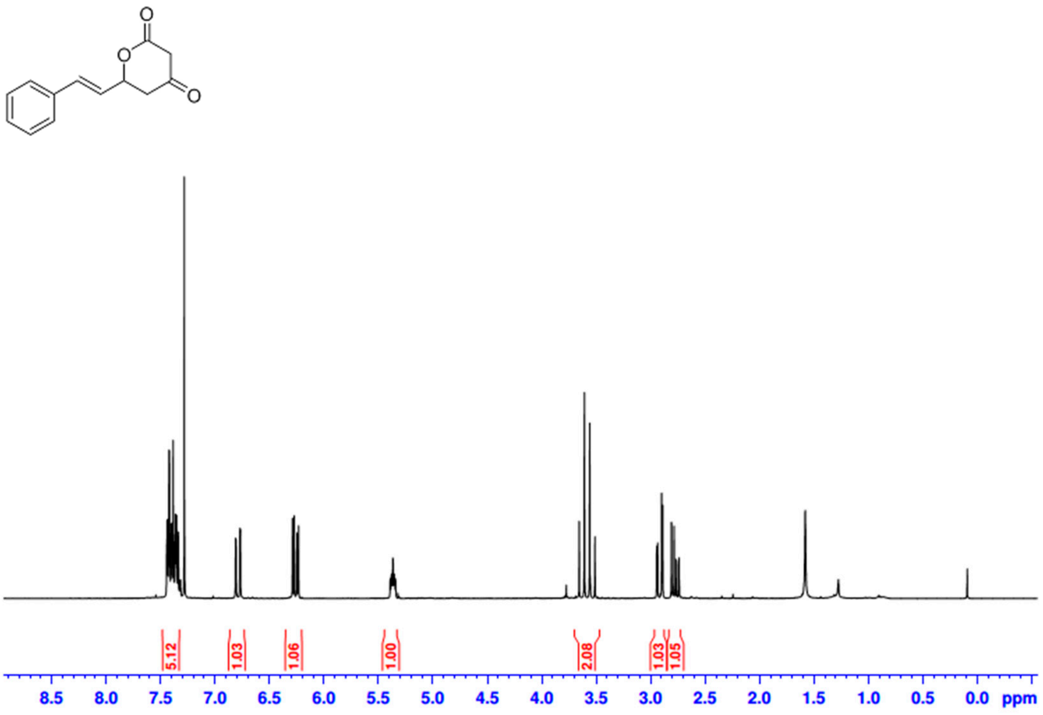

Figure S12. ^{1}H-NMR spectrum of compound ***rac*-14c**. The experiment was conducted at 400 MHz in $CDCl_3$.

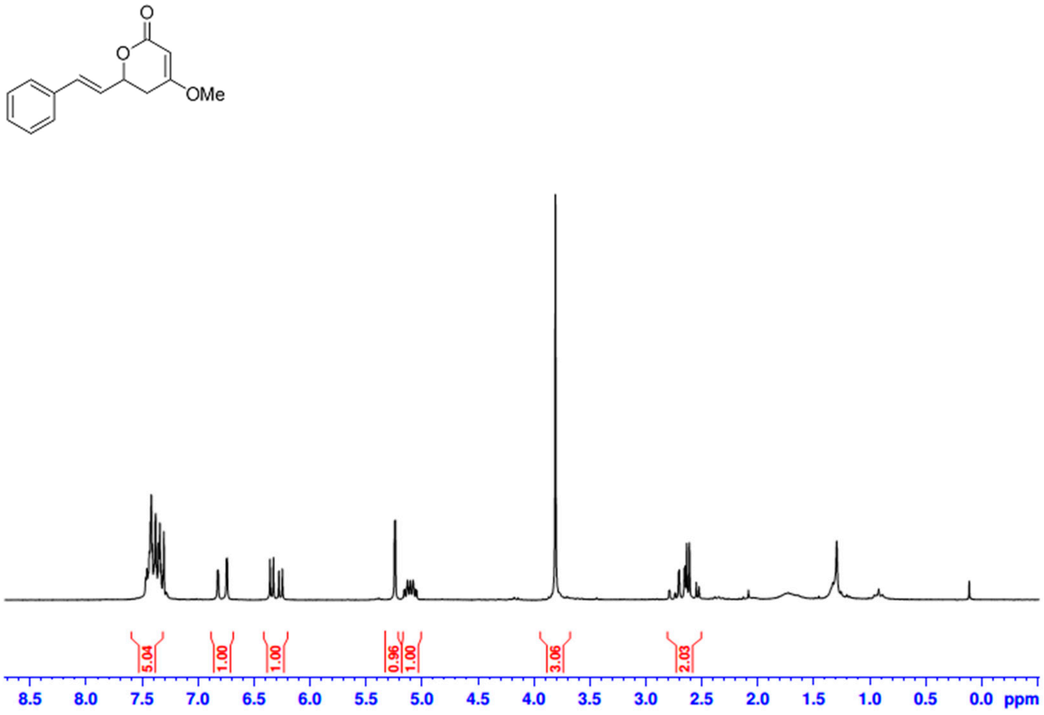

Figure S13. ^{1}H-NMR spectrum of compound ***rac*-15c**. The experiment was conducted at 400 MHz in $CDCl_3$.

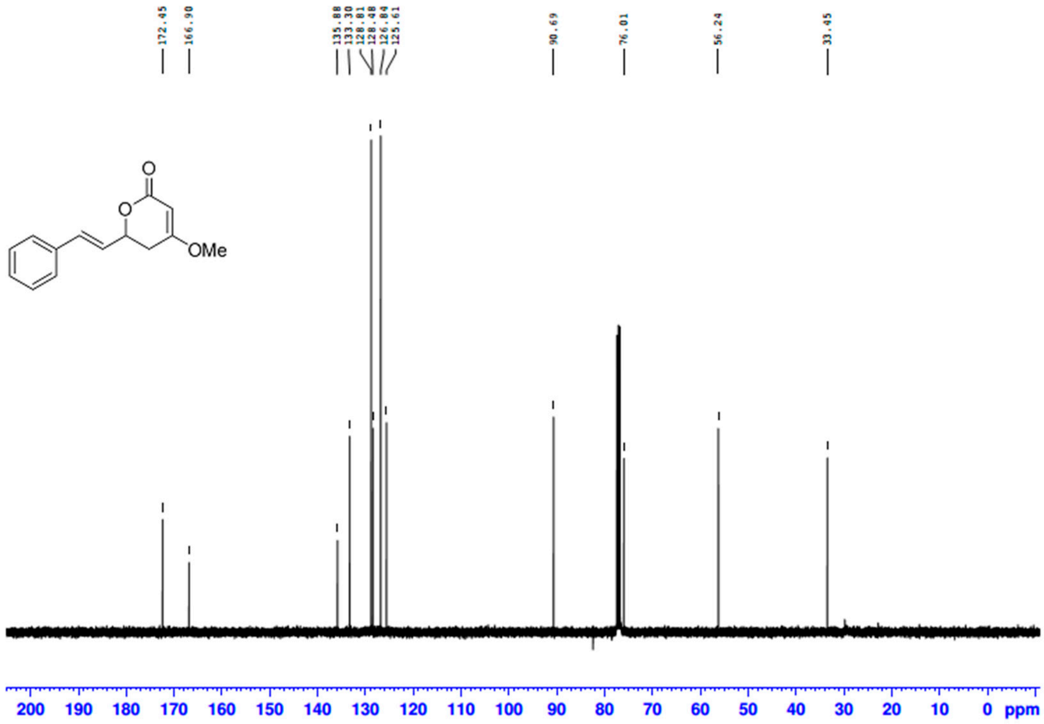

Figure S14. ^{13}C-NMR spectrum of compound ***rac*-15c**. The experiment was conducted at 100 MHz in $CDCl_3$.

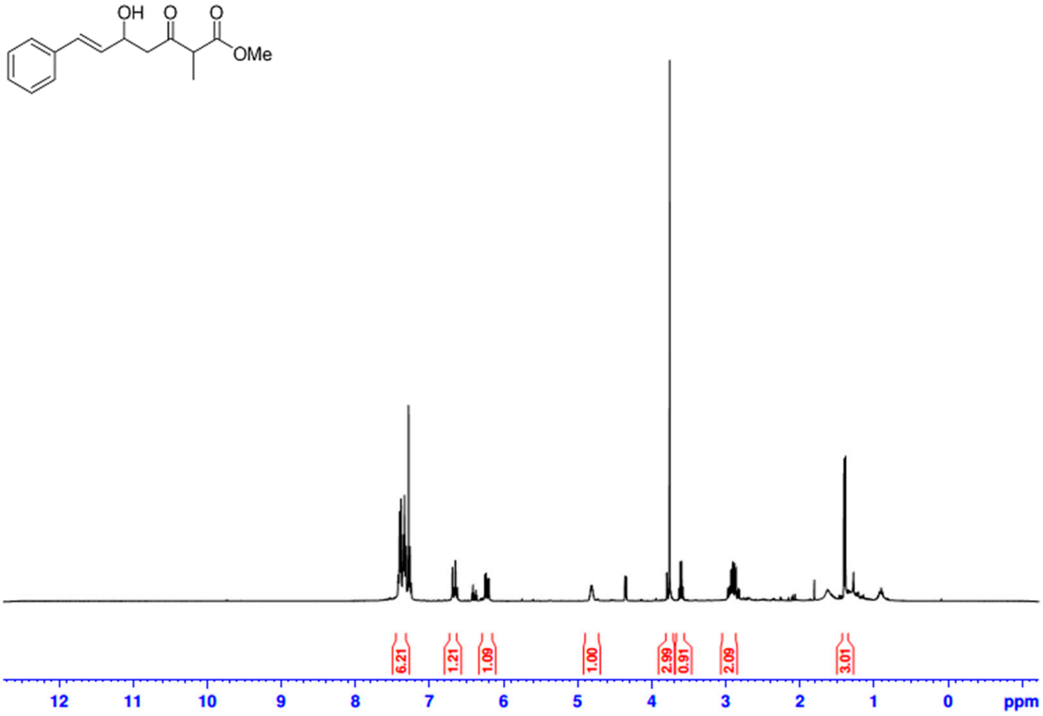

Figure S15. ^{1}H-NMR spectrum of compound ***rac*-13f**. The experiment was conducted at 400 MHz in $CDCl_3$.

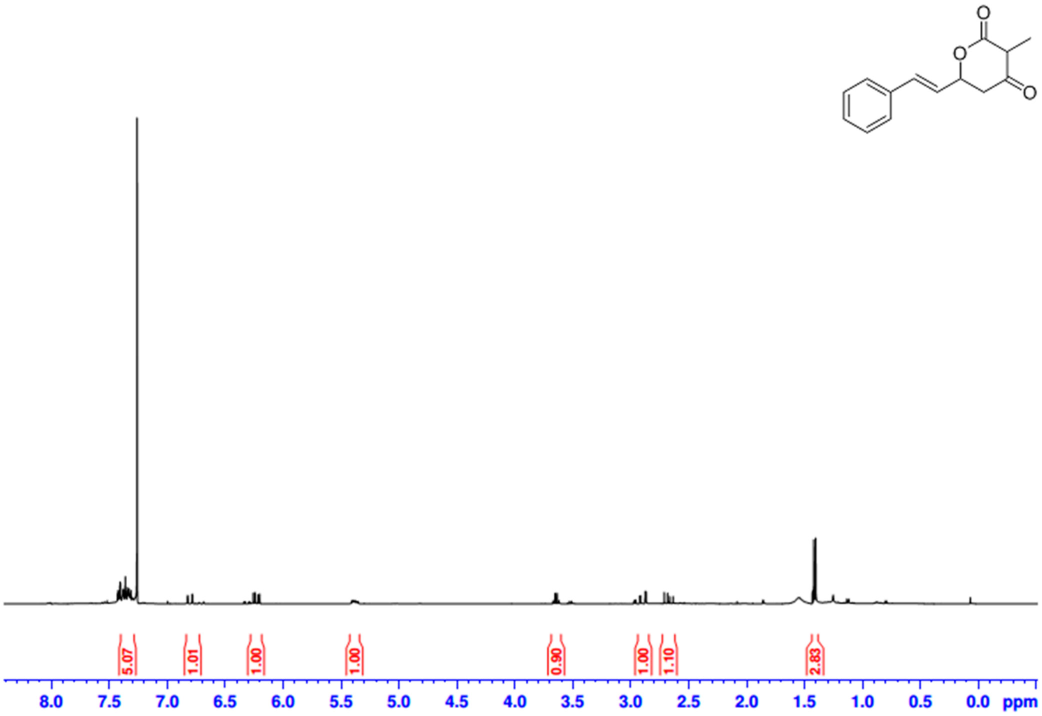

Figure S16. ^{1}H-NMR spectrum of compound ***rac*-14f**. The experiment was conducted at 400 MHz in $CDCl_3$.

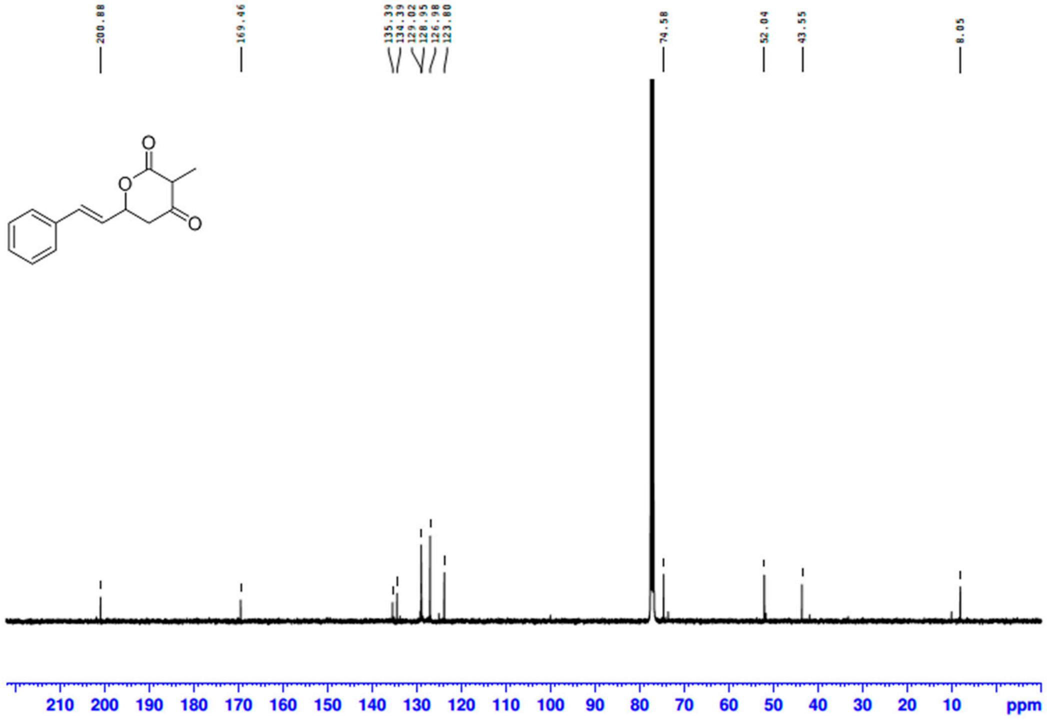

Figure S17. ^{13}C-NMR spectrum of compound ***rac*-14f**. The experiment was conducted at 100 MHz in $CDCl_3$.

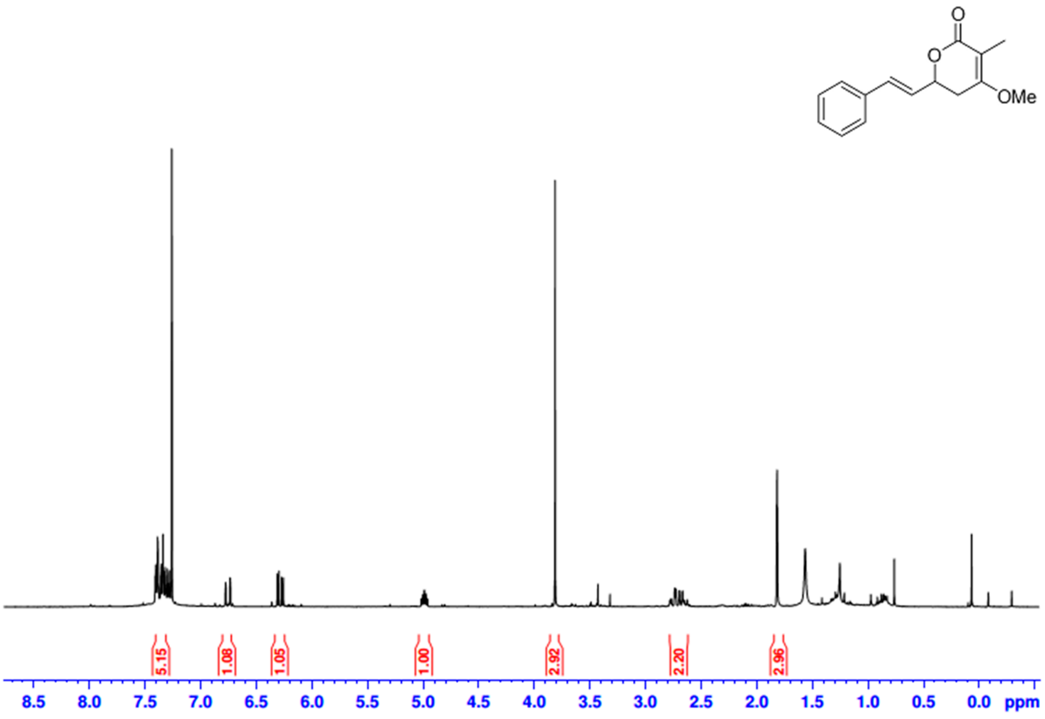

Figure S18. ^{1}H-NMR spectrum of compound ***rac*-15f**. The experiment was conducted at 400 MHz in $CDCl_3$.

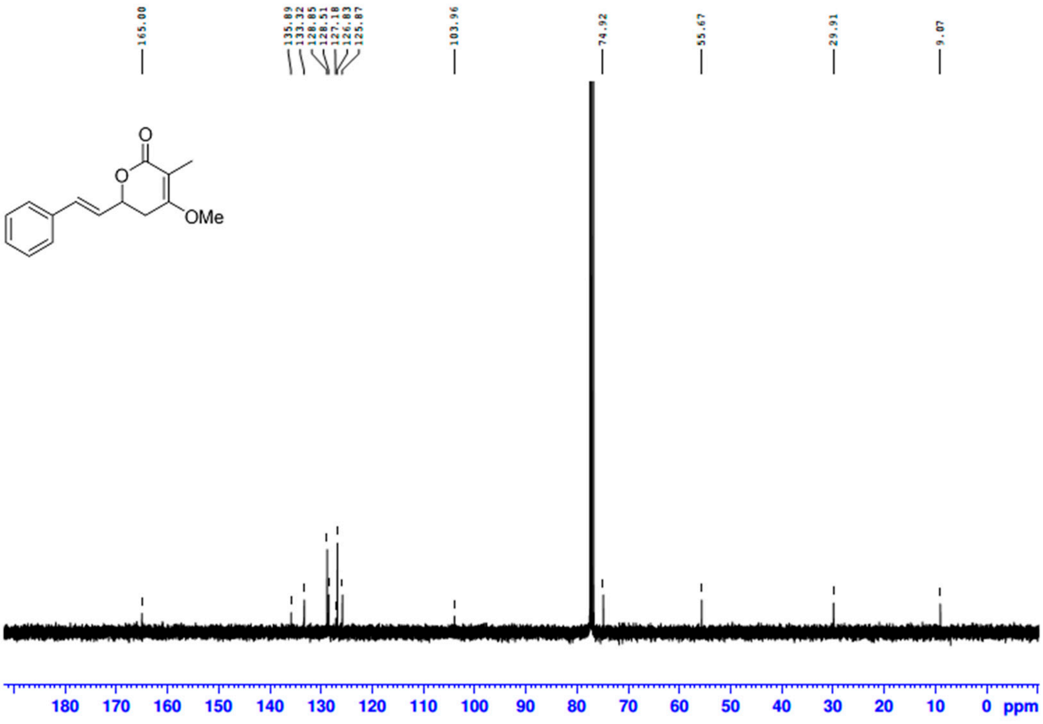

Figure S19. ^{13}C-NMR spectrum of compound ***rac*-15f**. The experiment was conducted at 100 MHz in $CDCl_3$.

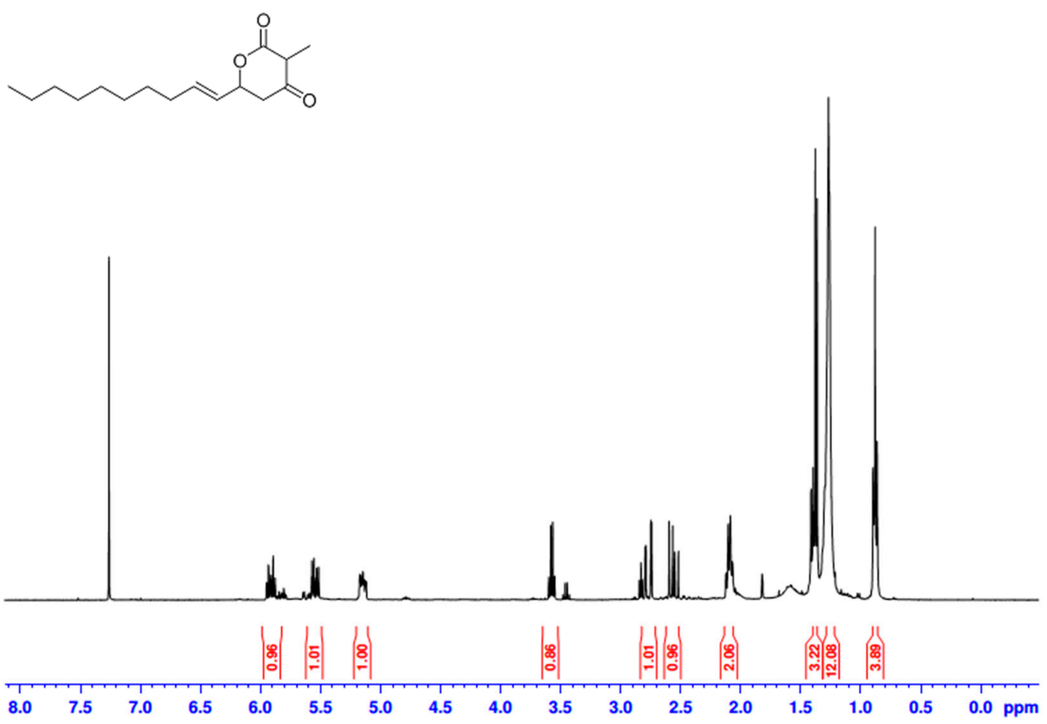

Figure S20. ^{1}H-NMR spectrum of compound ***rac*-14g**. The experiment was conducted at 400 MHz in $CDCl_3$.

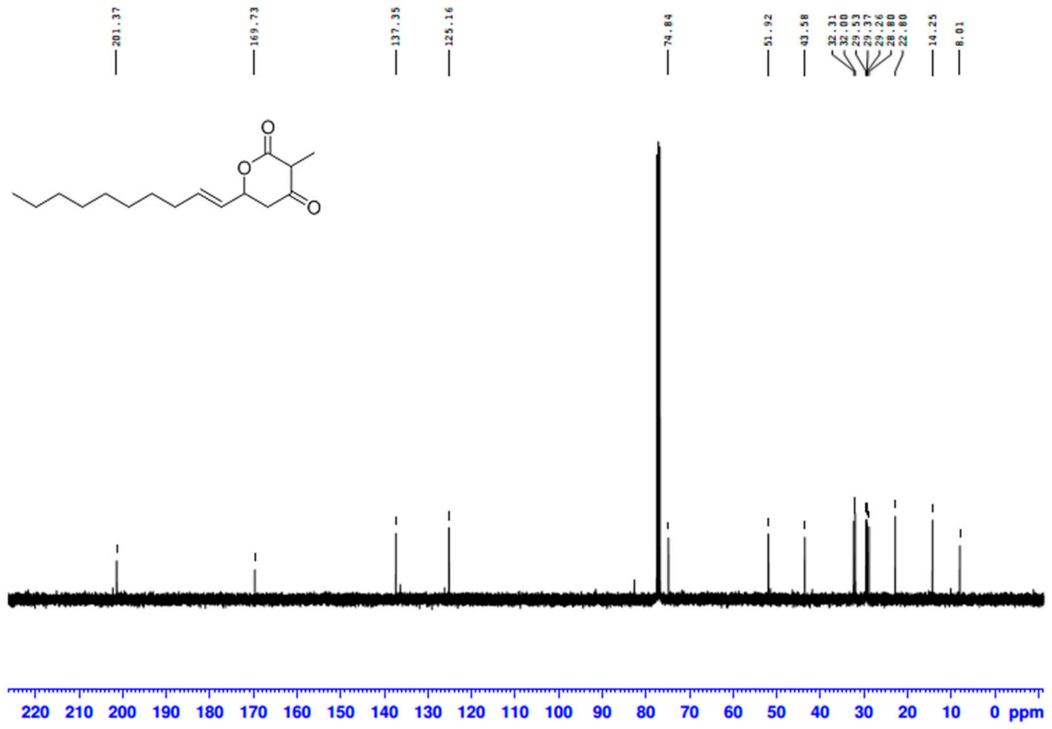

Figure S21. ^{13}C-NMR spectrum of compound ***rac*-14g**. The experiment was conducted at 100 MHz in $CDCl_3$.

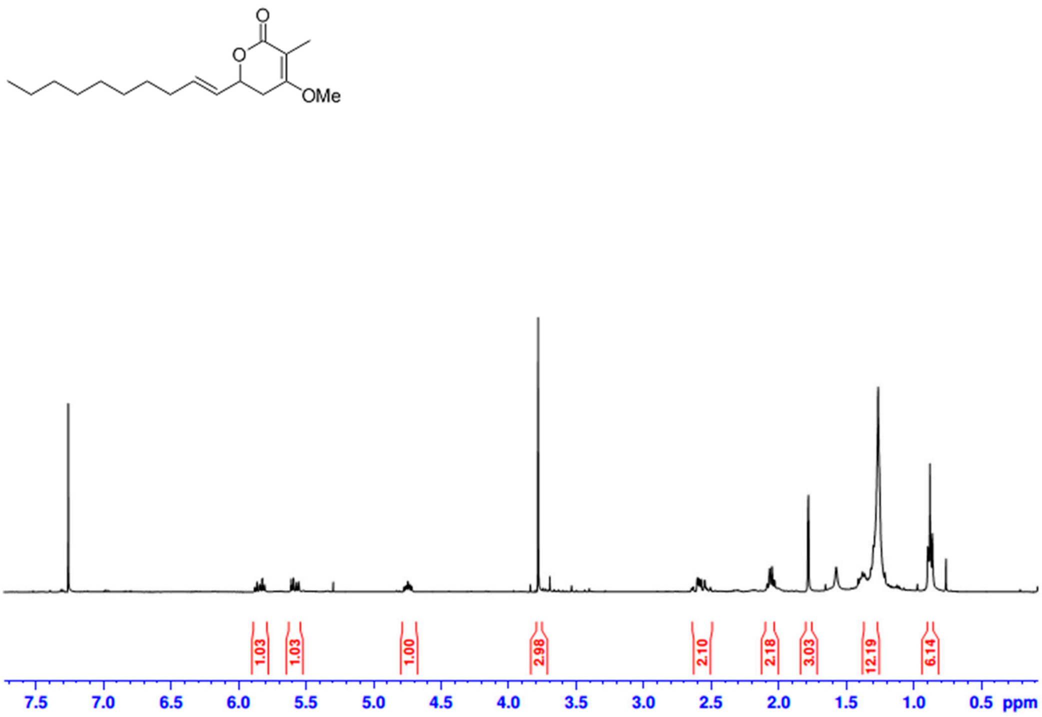

Figure S22. ^{1}H-NMR spectrum of compound ***rac*-15g**. The experiment was conducted at 400 MHz in $CDCl_3$.

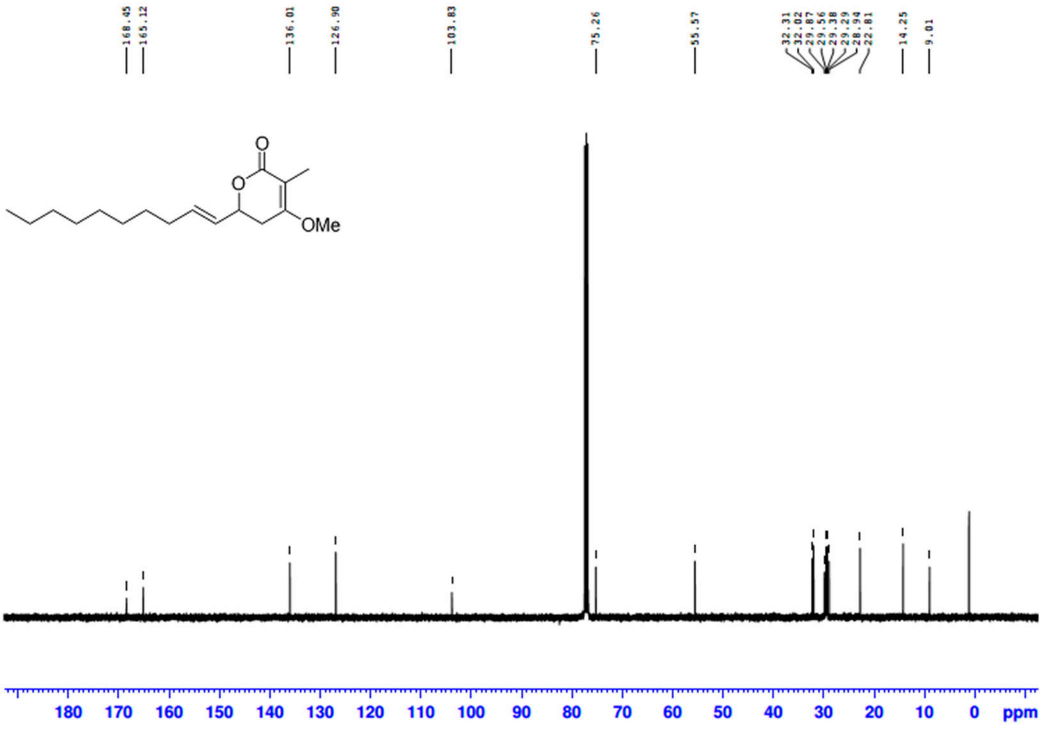

Figure S23. ^{13}C-NMR spectrum of compound ***rac*-15g**. The experiment was conducted at 100 MHz in $CDCl_3$.

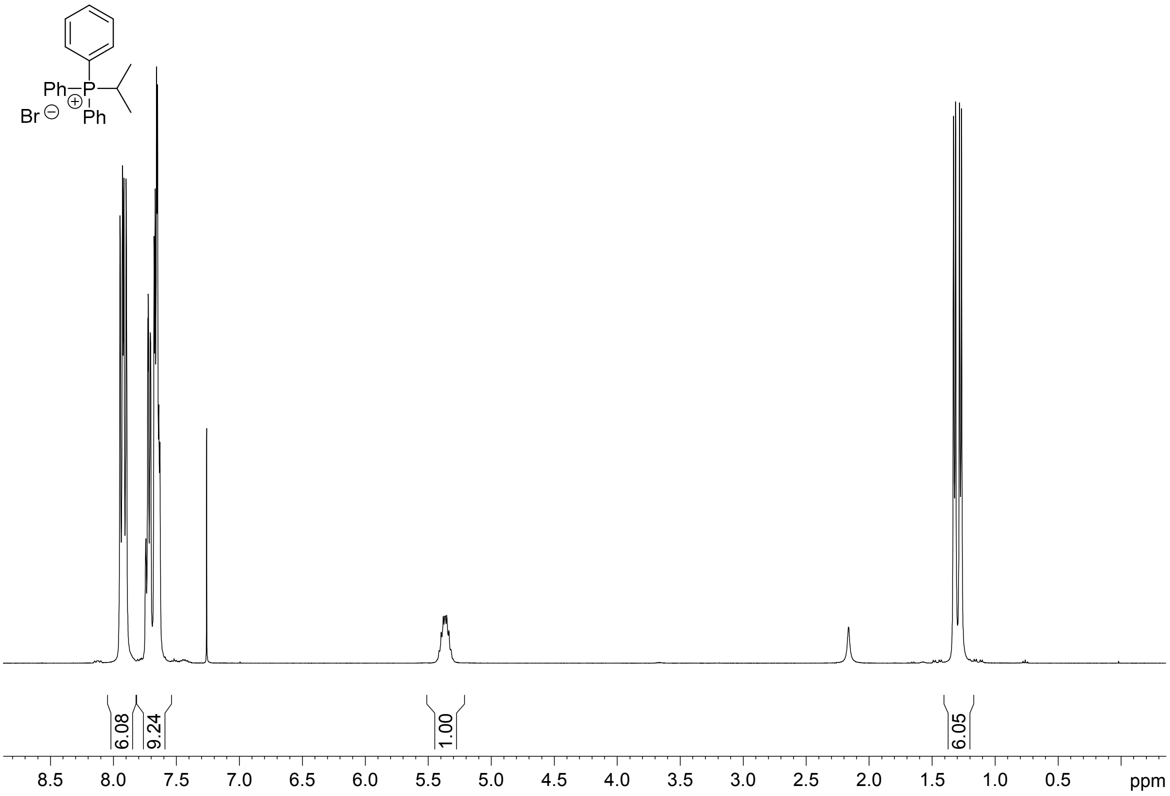

Figure S24. ^{1}H-NMR spectrum of compound isopropyltriphenylphosphonium bromide. The experiment was conducted at 400 MHz in $CDCl_3$.

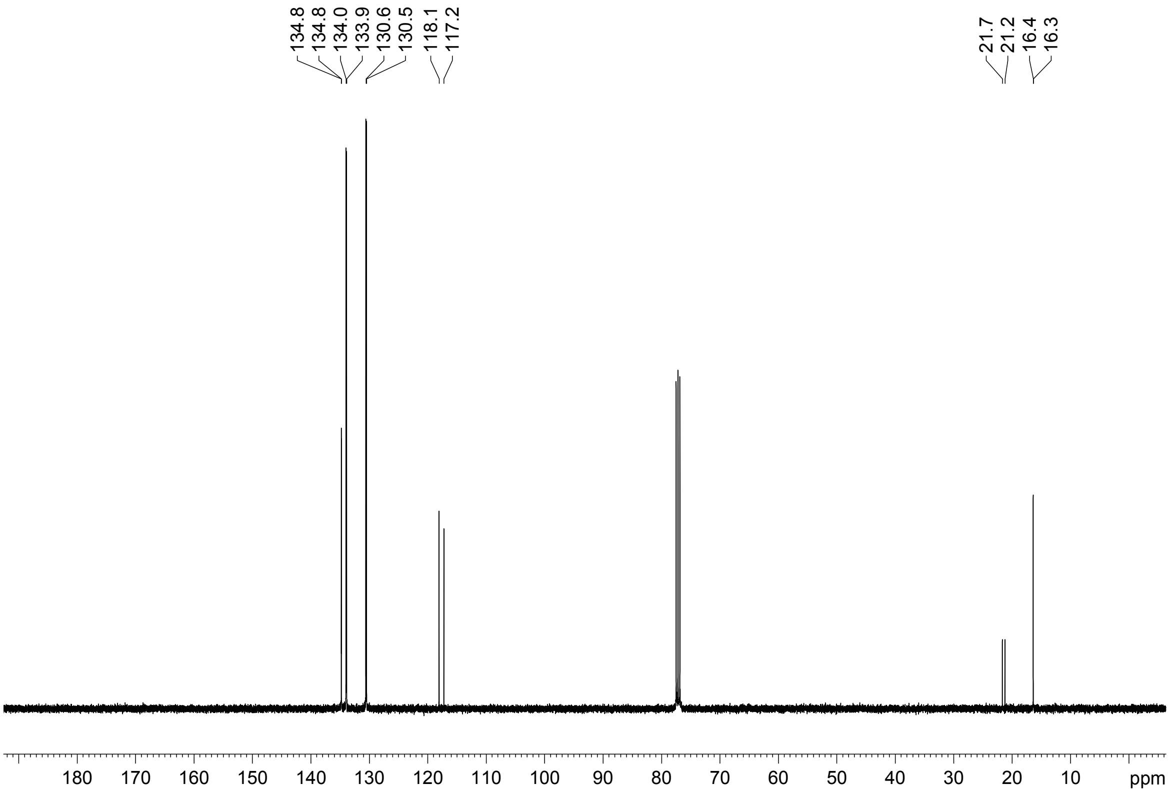

Figure S25. ^{13}C-NMR spectrum of compound isopropyltriphenylphosphonium bromide. The experiment was conducted at 100 MHz in $CDCl_3$.

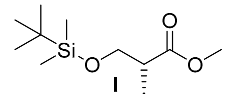

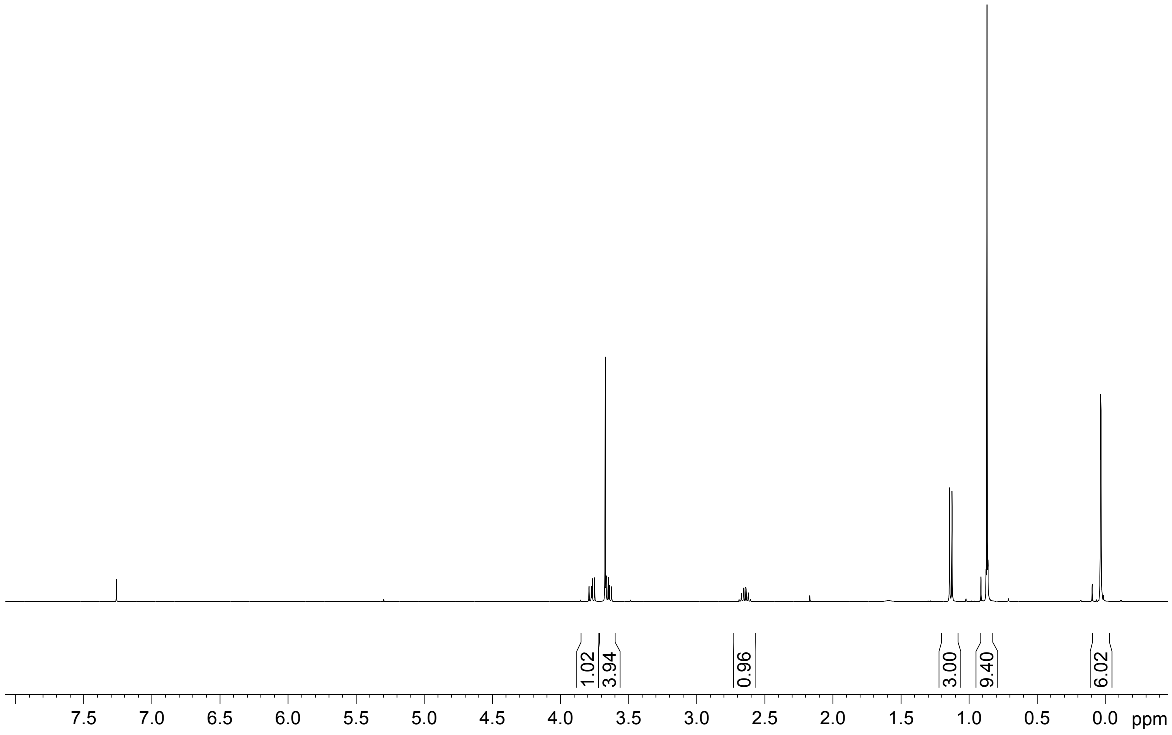

Figure S26. ^{1}H-NMR spectrum of compound **I**. The experiment was conducted at 400 MHz in $CDCl_3$.

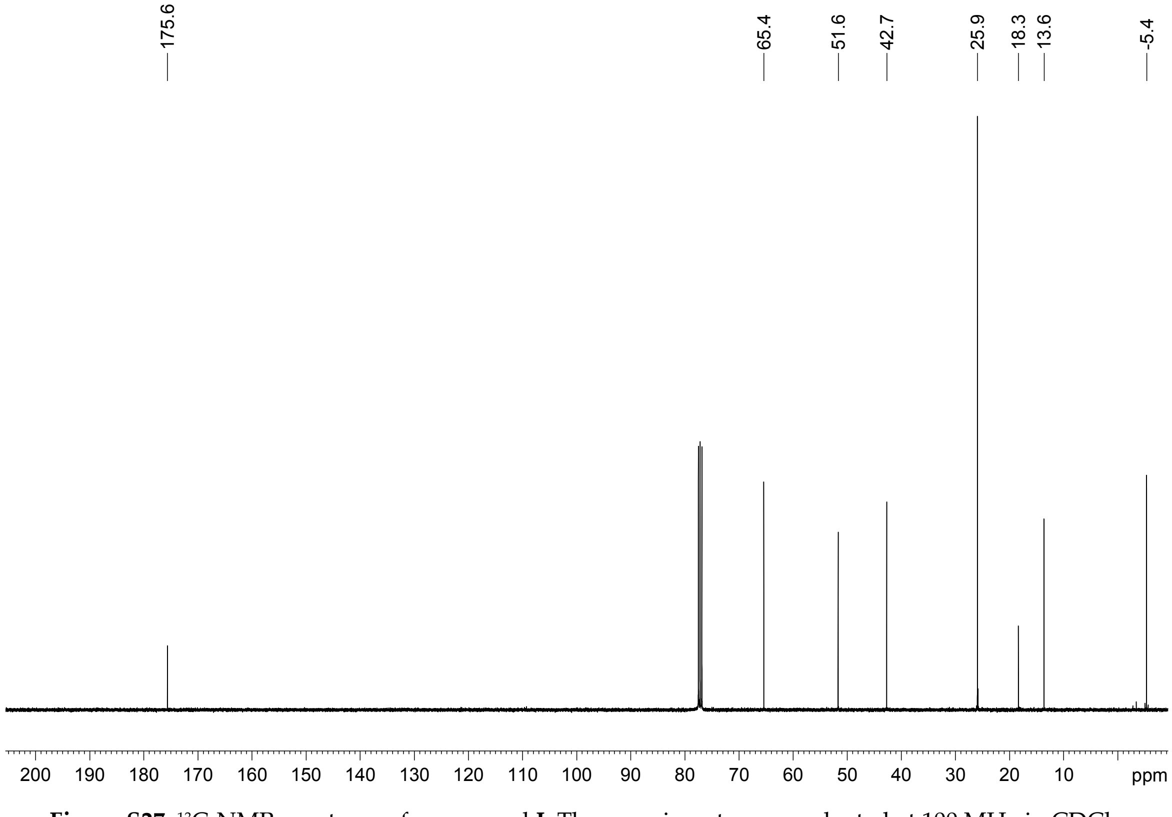

Figure S27. ^{13}C-NMR spectrum of compound **I**. The experiment was conducted at 100 MHz in $CDCl_3$.

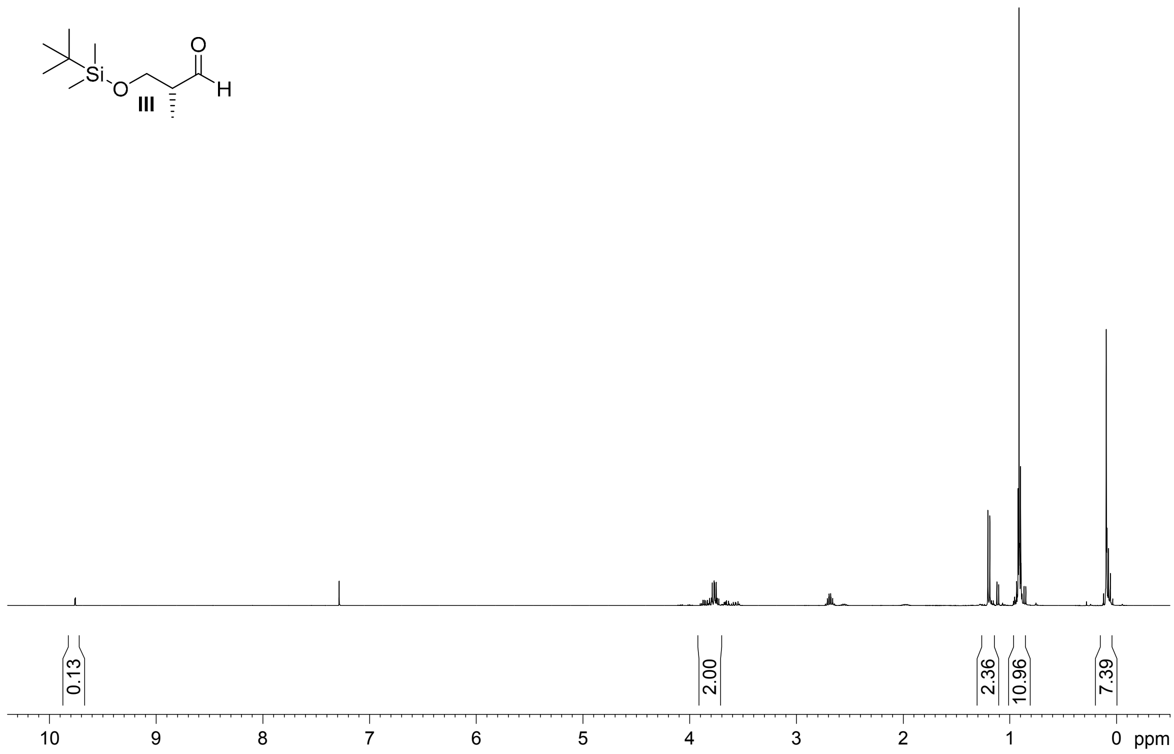

Figure S28. ^{1}H-NMR spectrum of compound **III**. The experiment was conducted at 400 MHz in $CDCl_3$.

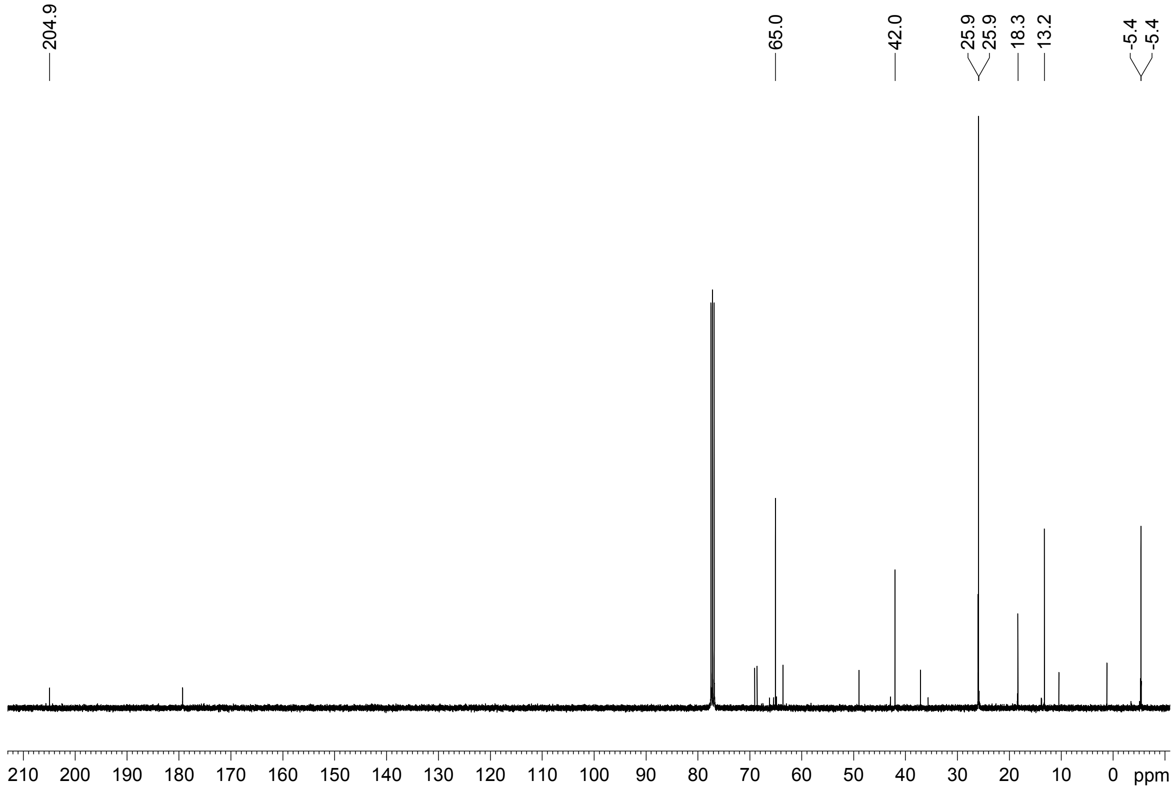

Figure S29. ^{13}C-NMR spectrum of compound **III**. The experiment was conducted at 100 MHz in $CDCl_3$.

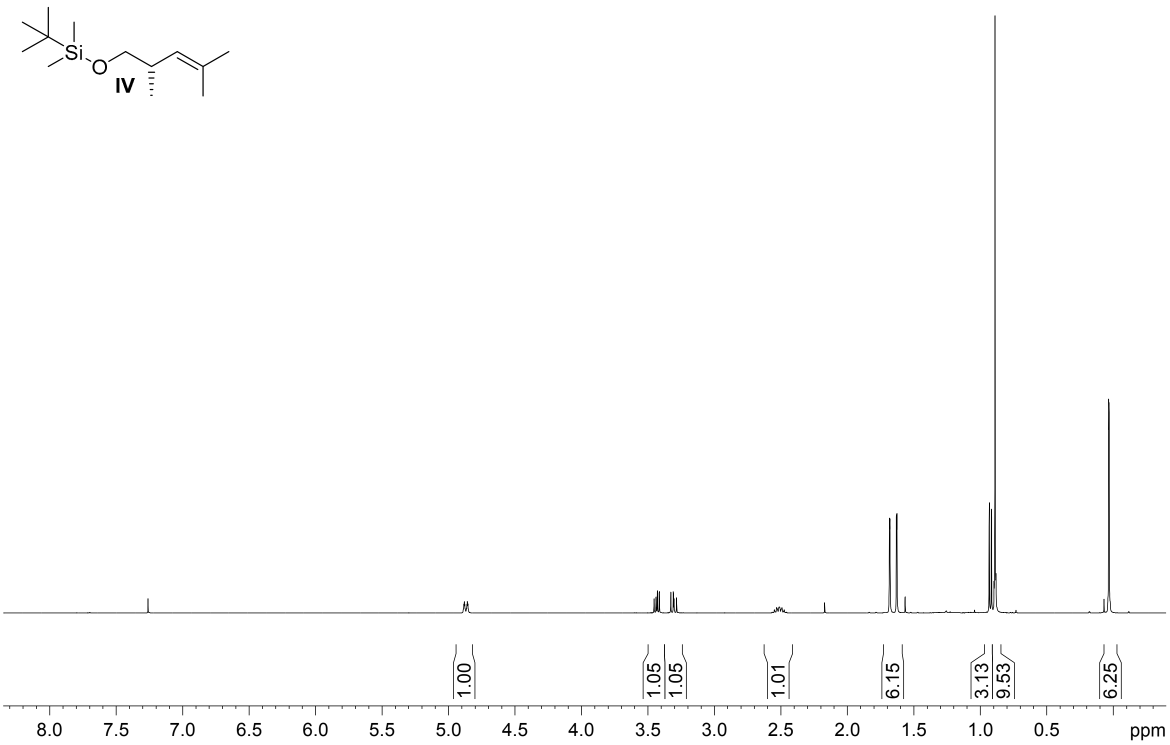

Figure S30. ^{1}H-NMR spectrum of compound **IV**. The experiment was conducted at 400 MHz in $CDCl_3$.

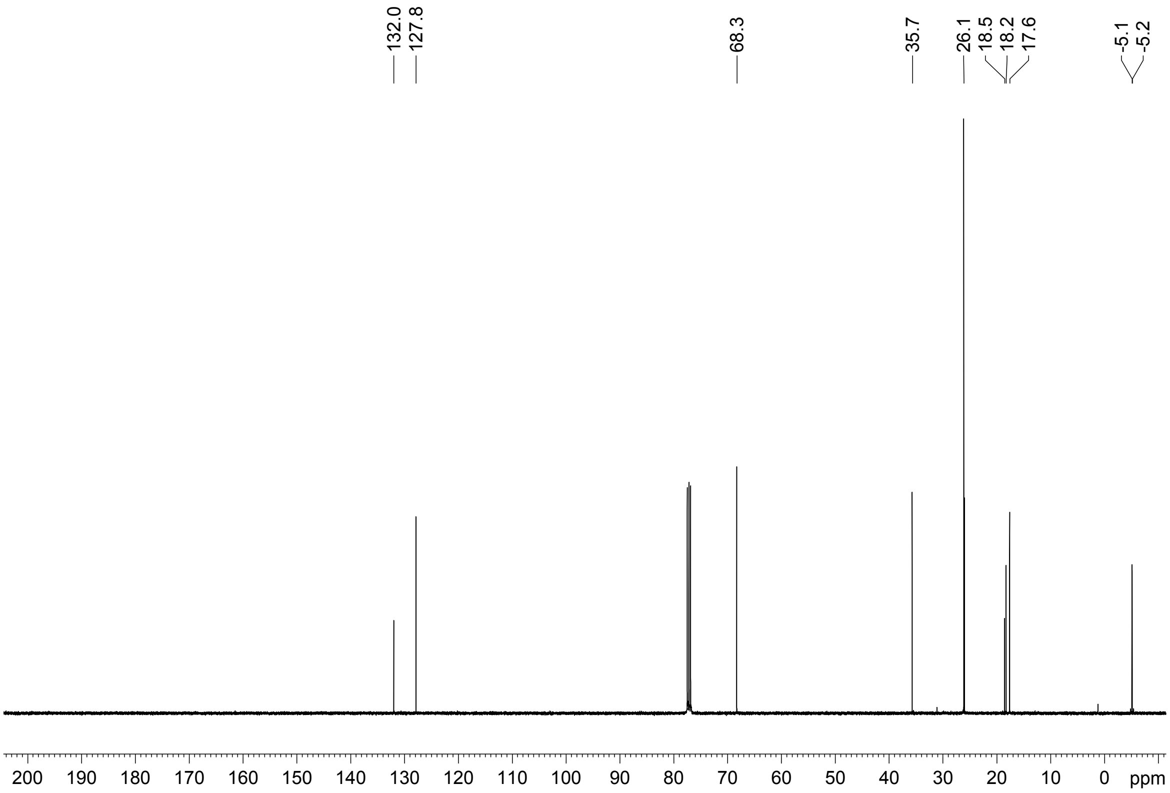

Figure S31. ^{13}C-NMR spectrum of compound **IV**. The experiment was conducted at 100 MHz in $CDCl_3$.

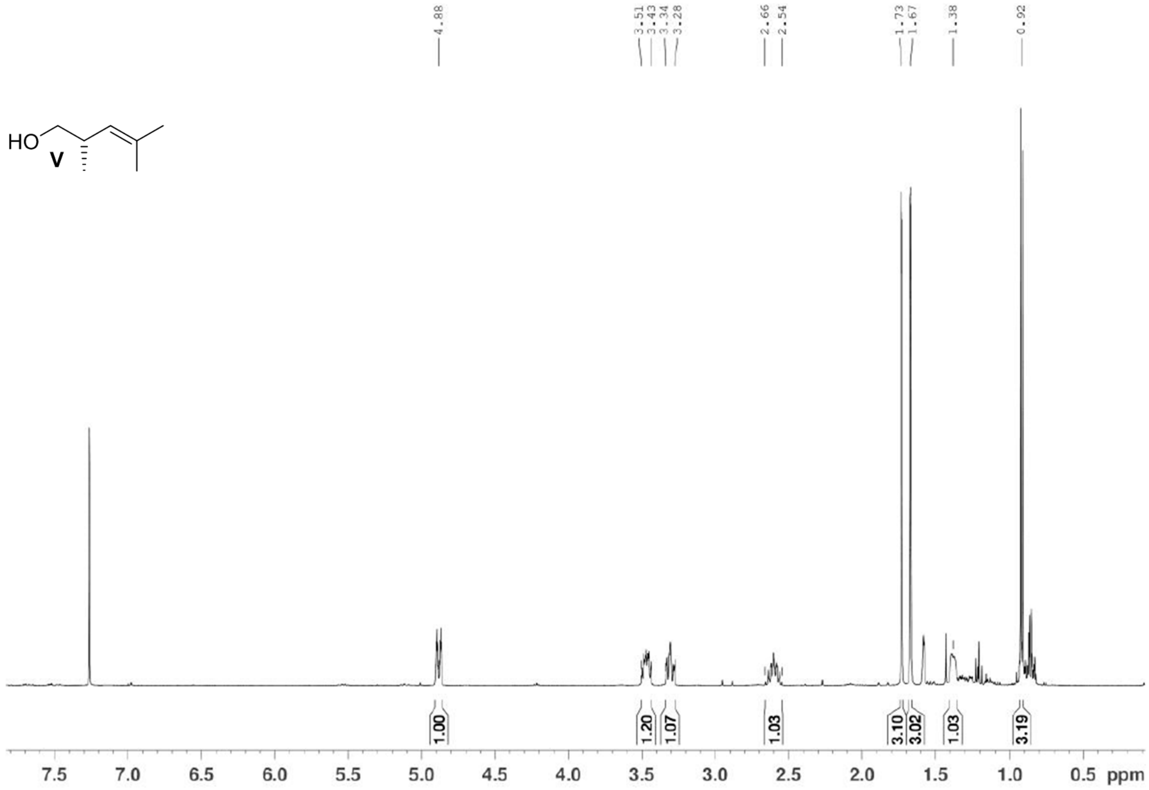

Figure S32. ^{1}H-NMR spectrum of compound **V**. The experiment was conducted at 400 MHz in $CDCl_3$.

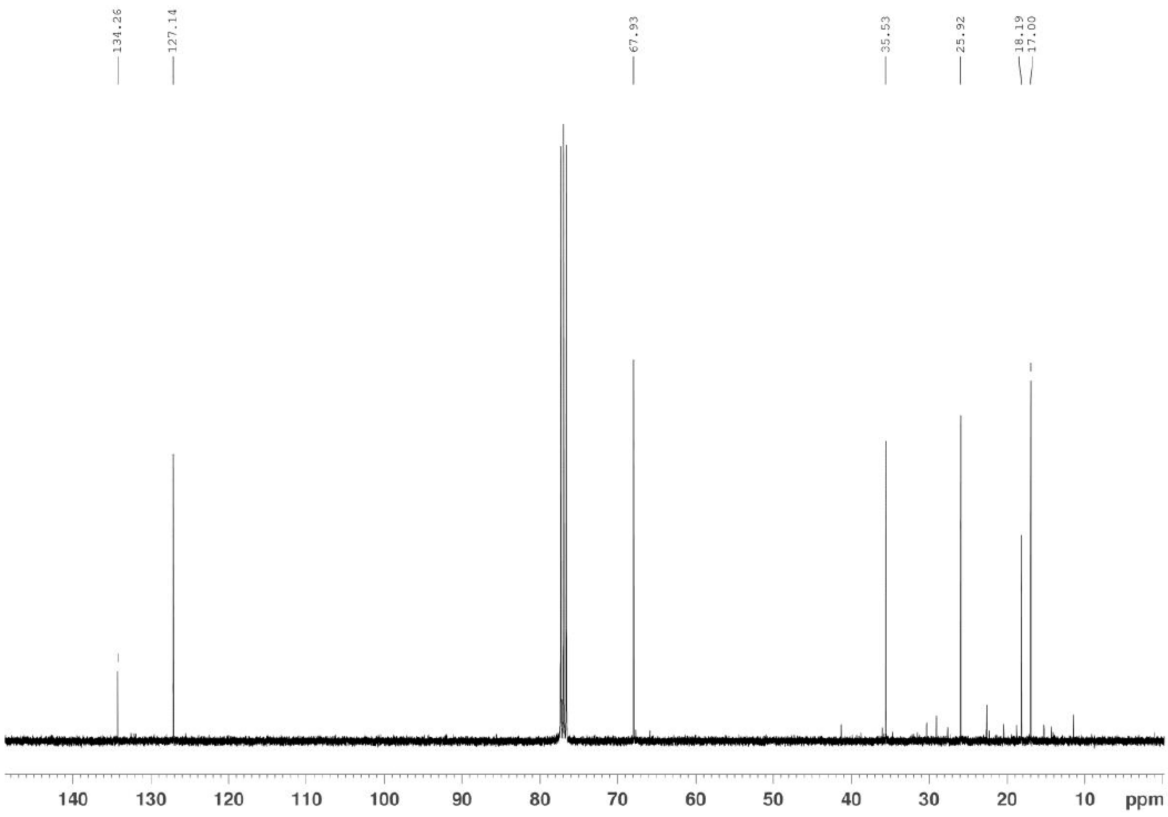

Figure S33. ^{13}C-NMR spectrum of compound **V**. The experiment was conducted at 100 MHz in $CDCl_3$.

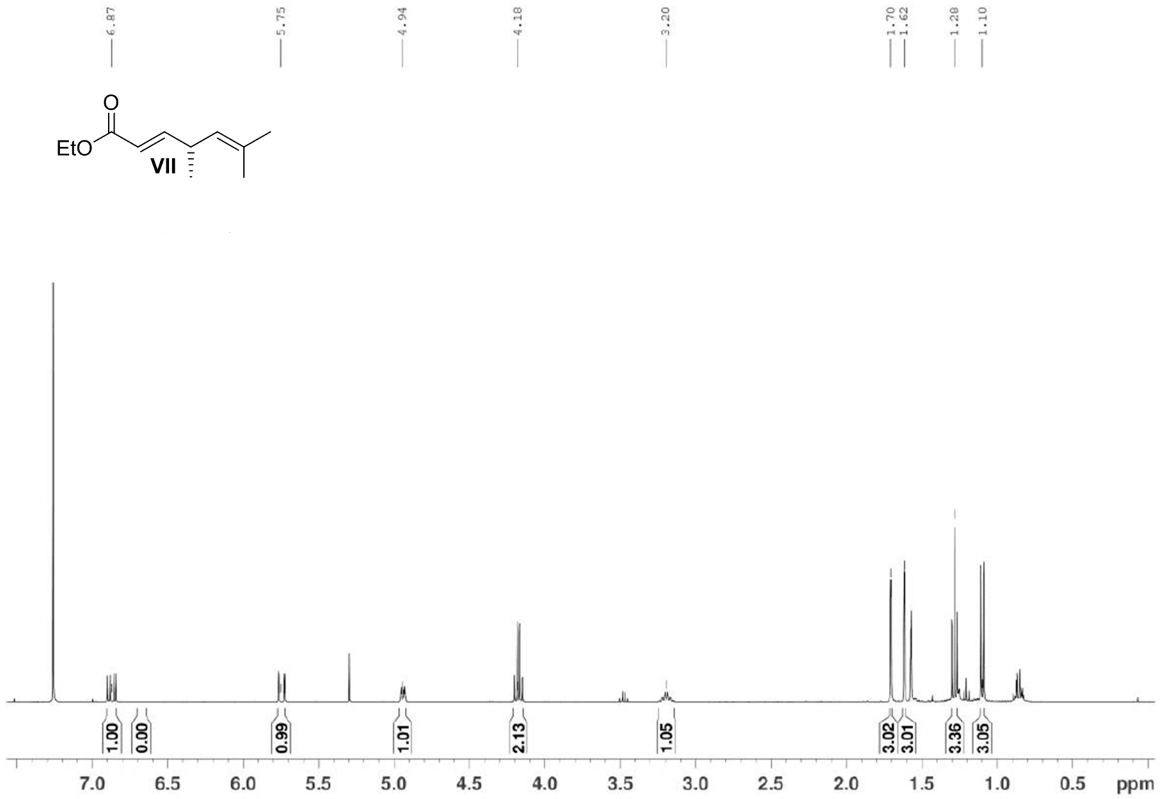

Figure S34. ^{1}H-NMR spectrum of compound **VII**. The experiment was conducted at 400 MHz in $CDCl_3$.

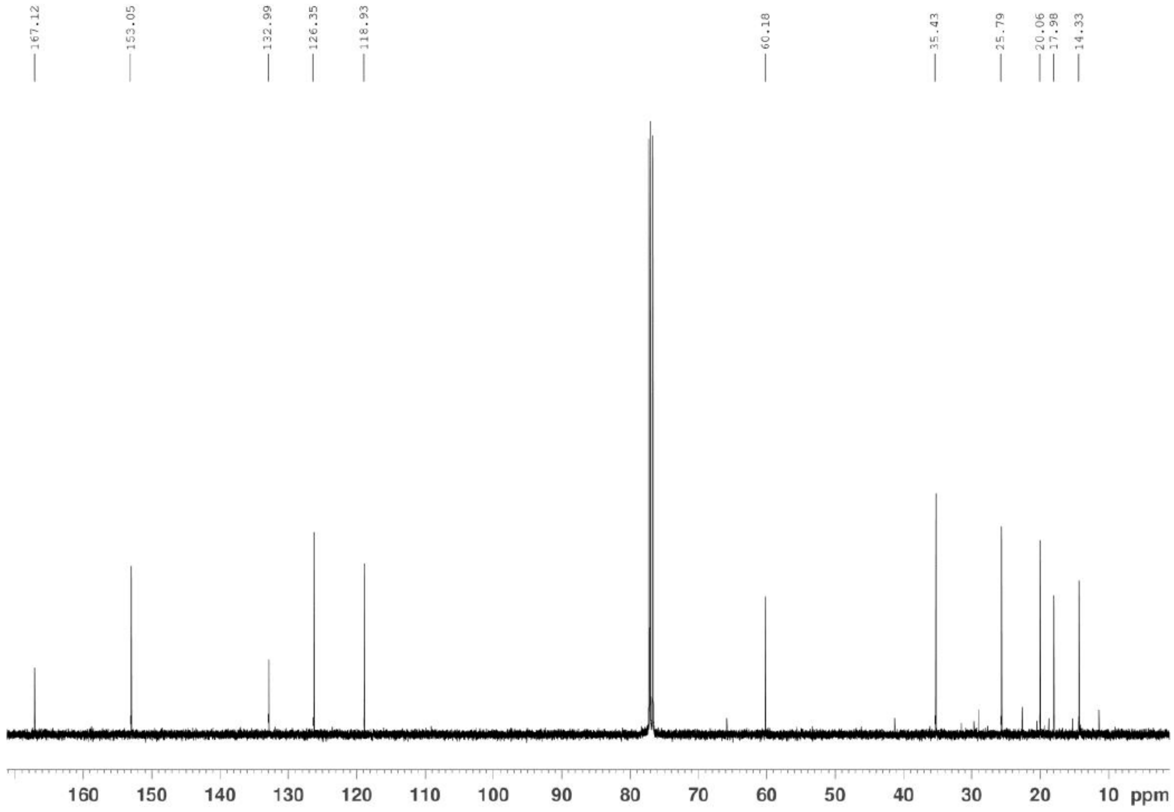

Figure S35. ^{13}C-NMR spectrum of compound **VII**. The experiment was conducted at 100 MHz in $CDCl_3$.

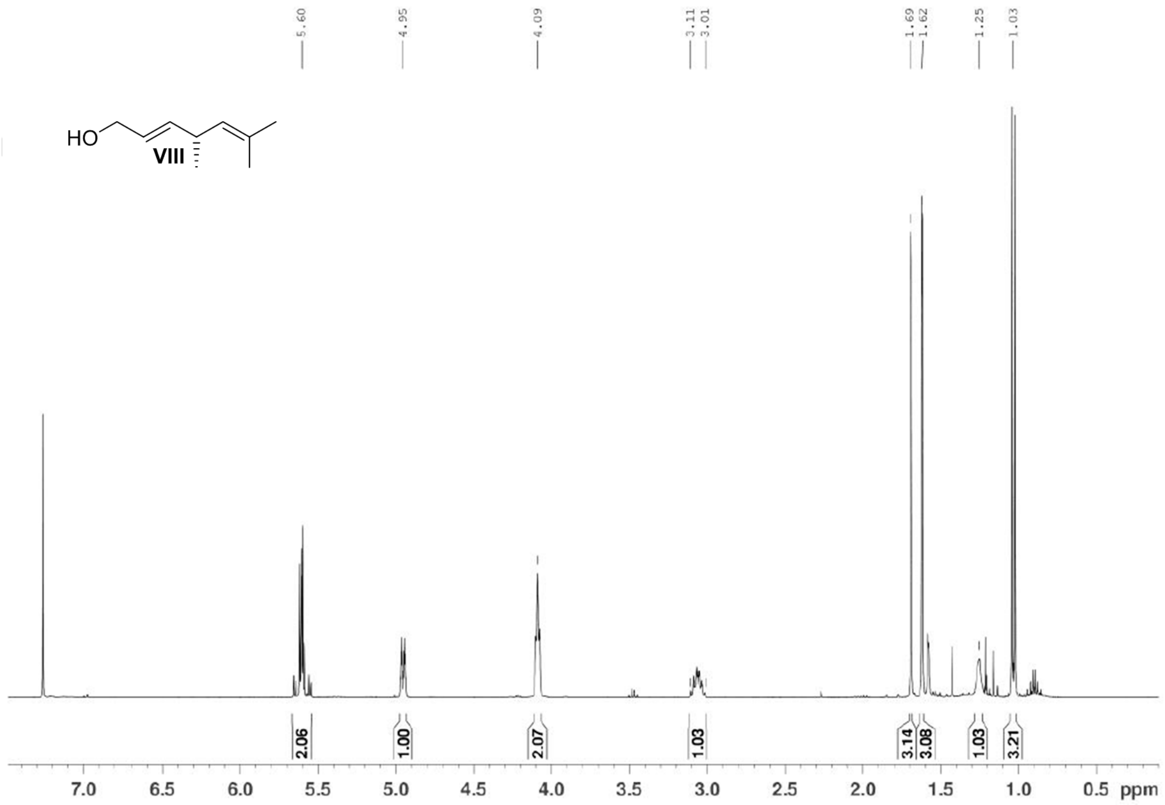

Figure S36. ^{1}H-NMR spectrum of compound **VIII**. The experiment was conducted at 400 MHz in $CDCl_3$.

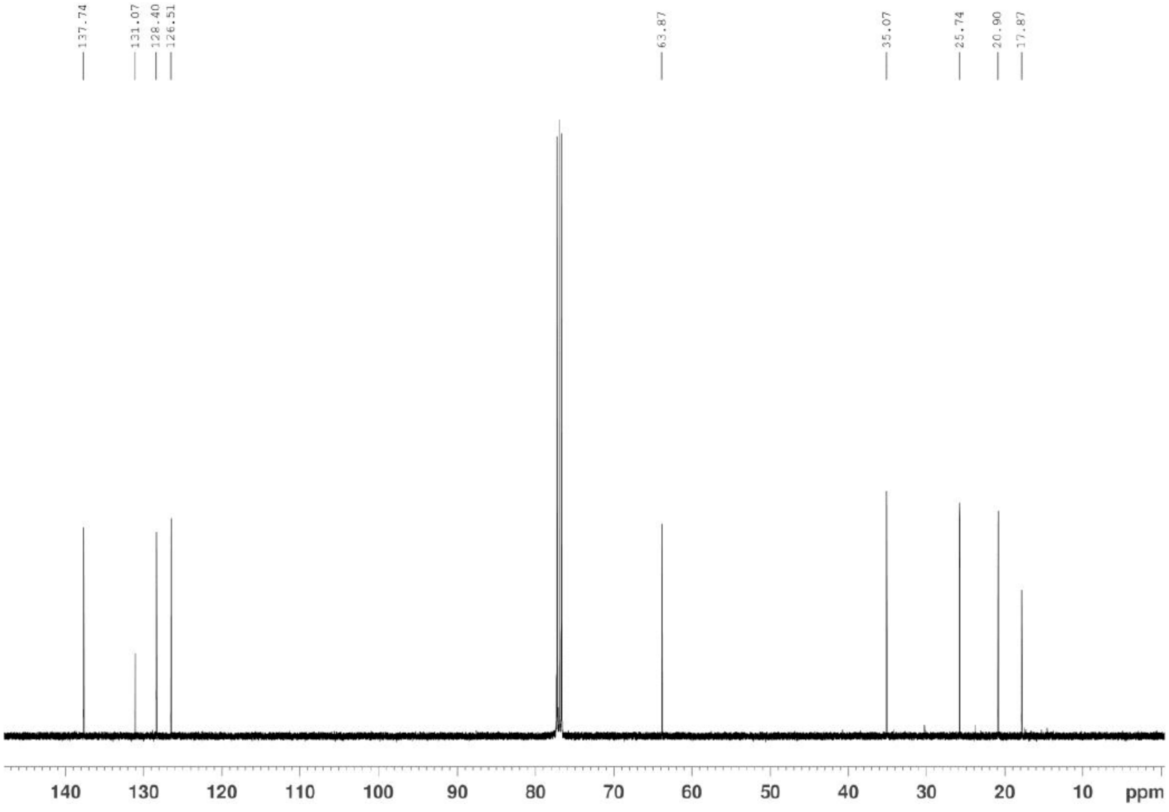

Figure S37. ^{13}C-NMR spectrum of compound **VIII**. The experiment was conducted at 100 MHz in $CDCl_3$.

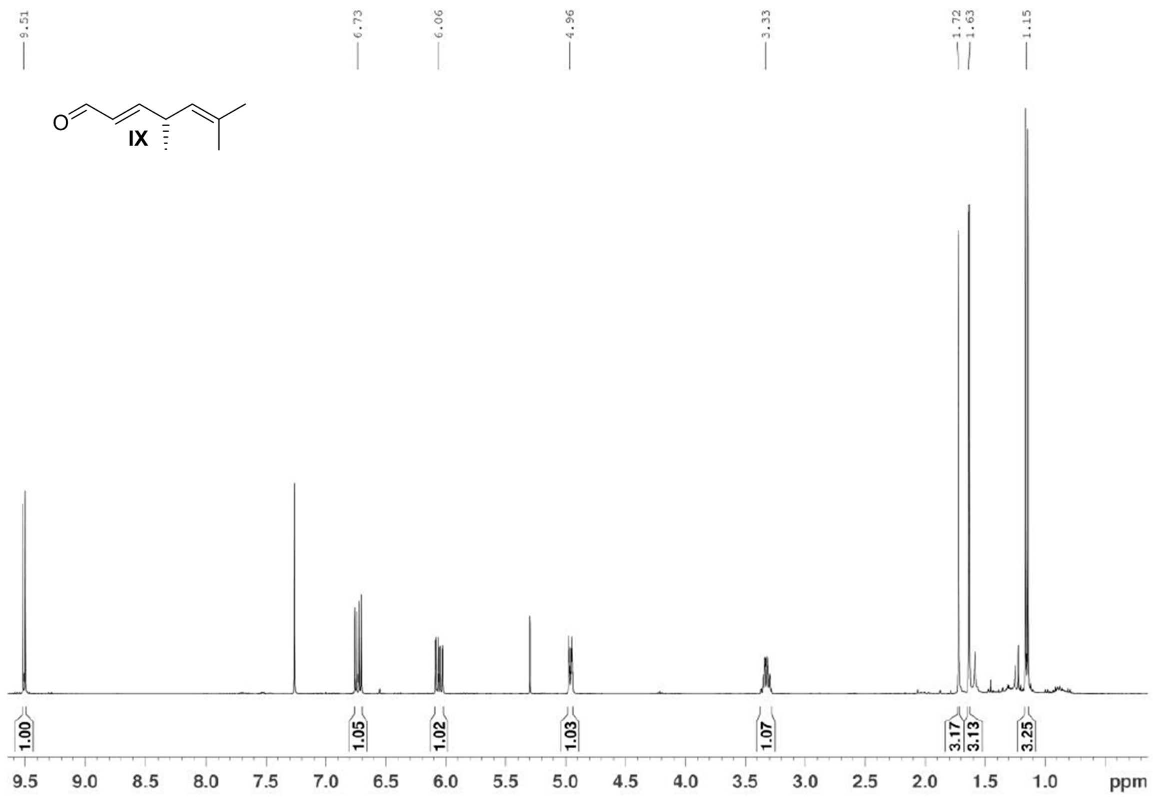

Figure S38. ^{1}H-NMR spectrum of compound **IX**. The experiment was conducted at 400 MHz in $CDCl_3$.

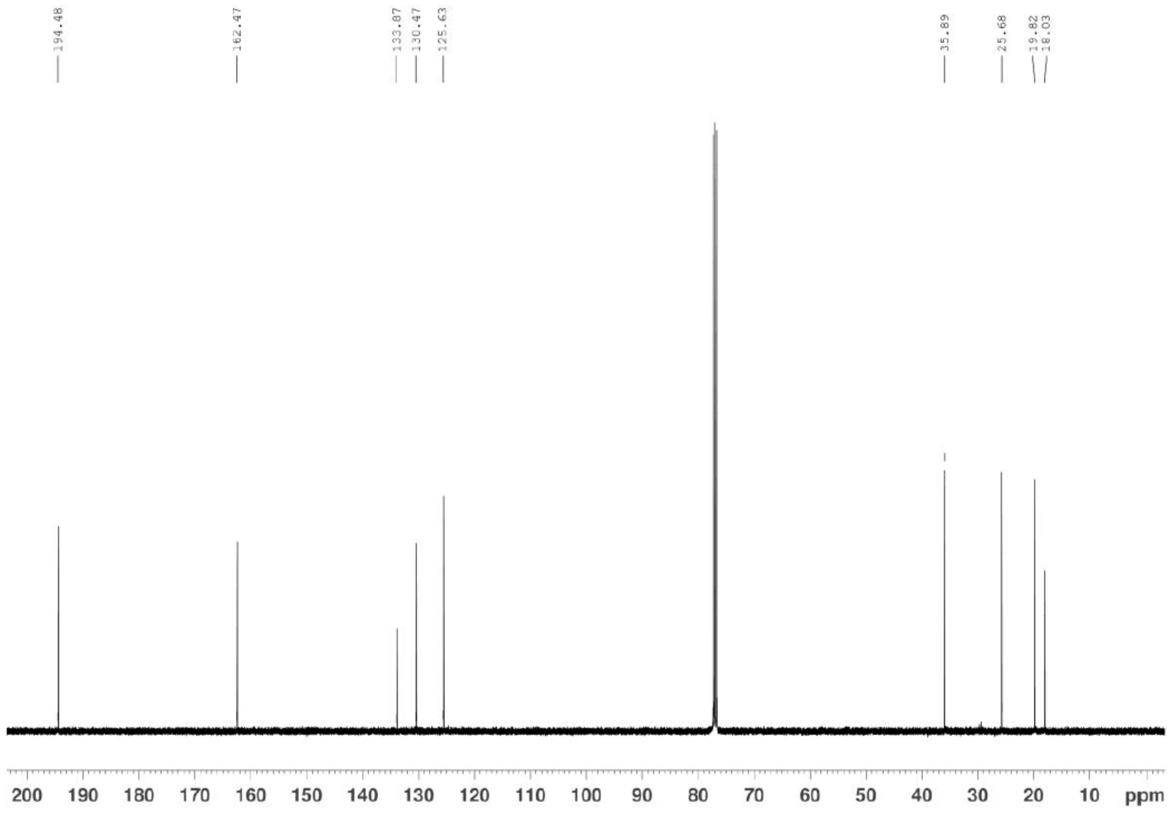

Figure S39. ^{13}C-NMR spectrum of compound **IX**. The experiment was conducted at 100 MHz in $CDCl_3$.

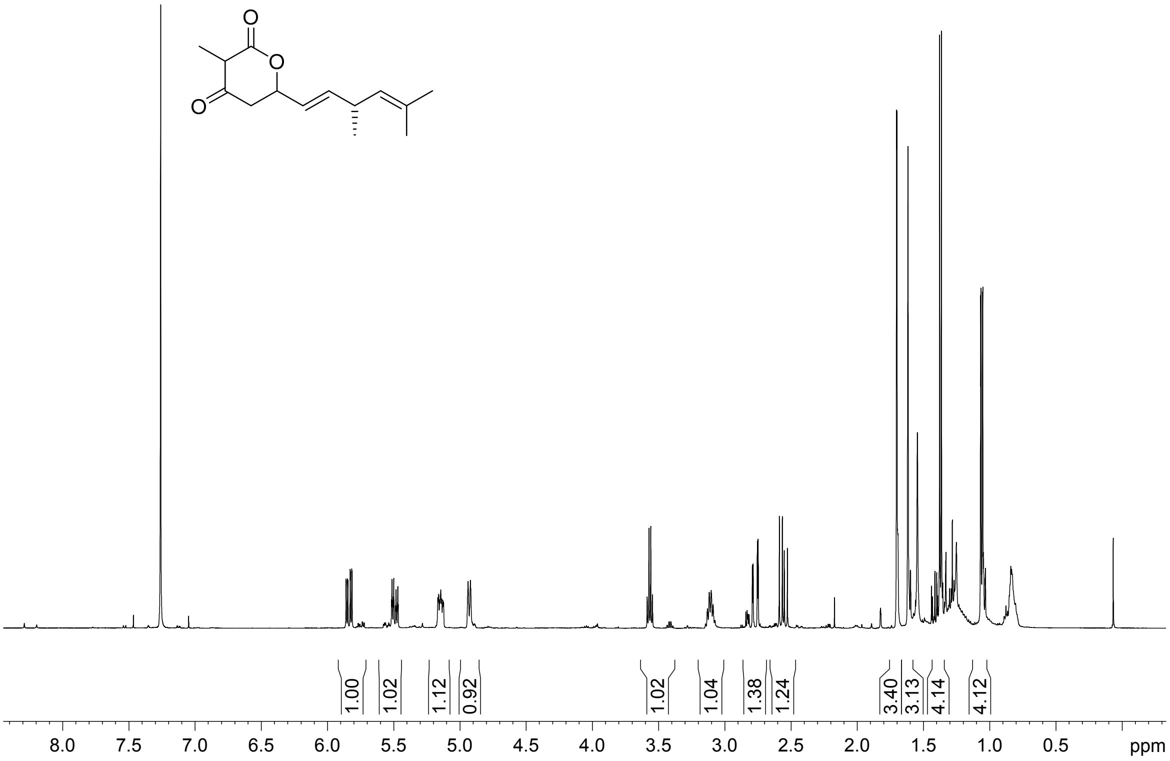

Figure S40. ^{1}H-NMR spectrum of compound ***rac*-14h**. The experiment was conducted at 400 MHz in $CDCl_3$.

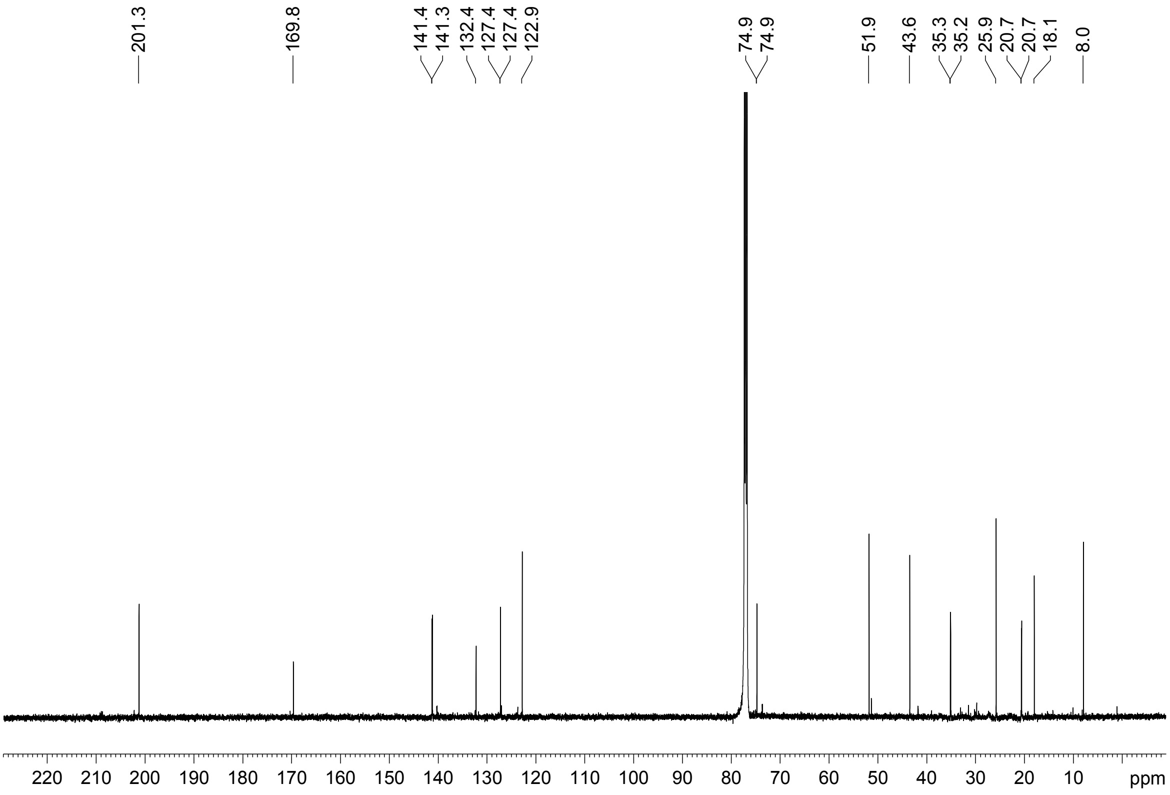

Figure S41. ^{13}C-NMR spectrum of compound ***rac*-14h**. The experiment was conducted at 100 MHz in $CDCl_3$.

2. Determination of the Preferred Configuration of 3-Methyl-6-vinyldihydro-2*H*-pyran-2,4(3*H*)-diones by NOE Correlation Spectroscopy

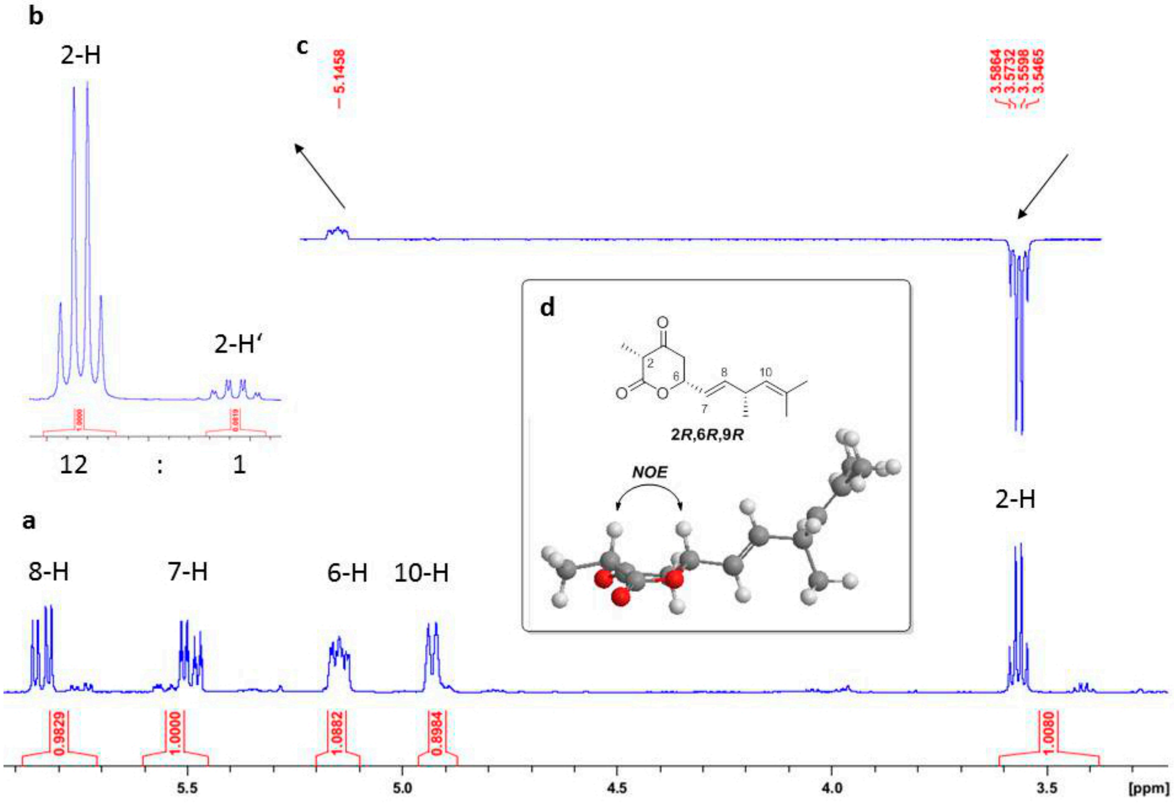

Figure S42. (**a**) ^{1}H-NMR spectrum of synthetic ***rac*-14h**; (**b**) The ratio of the relative arrangement of substituents on ring positions 3 and 6 was 12:1 (*syn:anti*); (**c**) NOE correlation spectrum of synthetic ***rac*-14h** ($CDCl_3$, 500 MHz). Irradiation on 6-H led to a correlation to 3-H; (**d**) structure of ***rac*-14h** in the preferred *syn* form. All spectra were recorded at 400 MHz in $CDCl_3$.

A sample of ***rac*-14h** was analysed by ^{1}H-NMR spectroscopy and NOE correlation spectroscopy in order to confirm the preferred relative configuration of ***rac*-14h** in solution. A clear preference for the *syn* arrangement of the substituents on the 3- and the 6-position of the ring is observed (ratio 12:1). The accumulation of the *syn* diastereomers could be explained by epimerisation occurring at C-3 under the conditions of the lactonisation.

3. NMR Analysis of the Semipreparative Scale Conversions

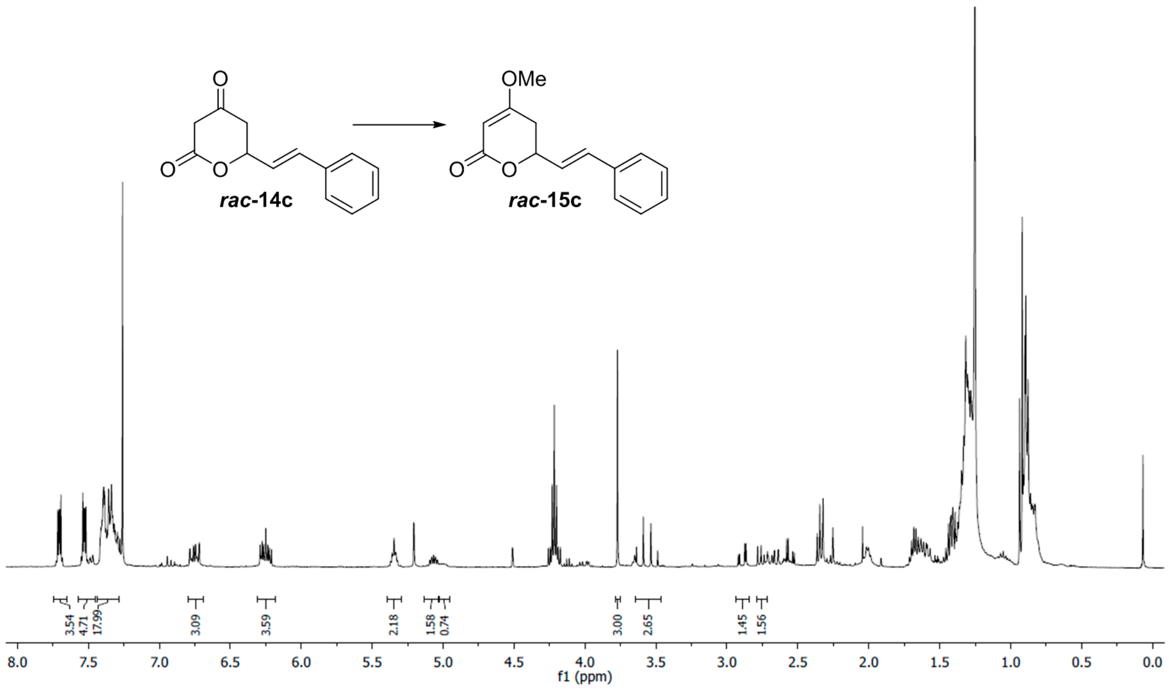

Figure S43. ^{1}H-NMR spectrum of the JerF conversion assay with compound ***rac*-14c** ($CDCl_3$, 400 MHz).

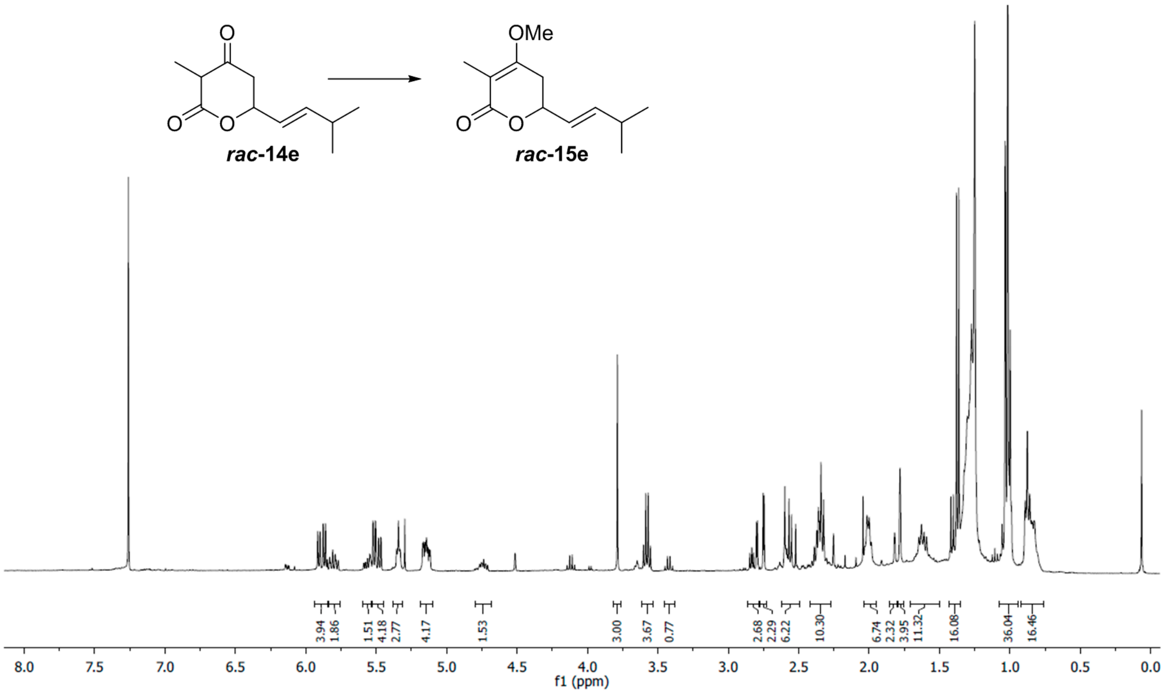

Figure S44. ^{1}H-NMR spectrum of the JerF conversion assay with compound ***rac*-14e** ($CDCl_3$, 400 MHz).

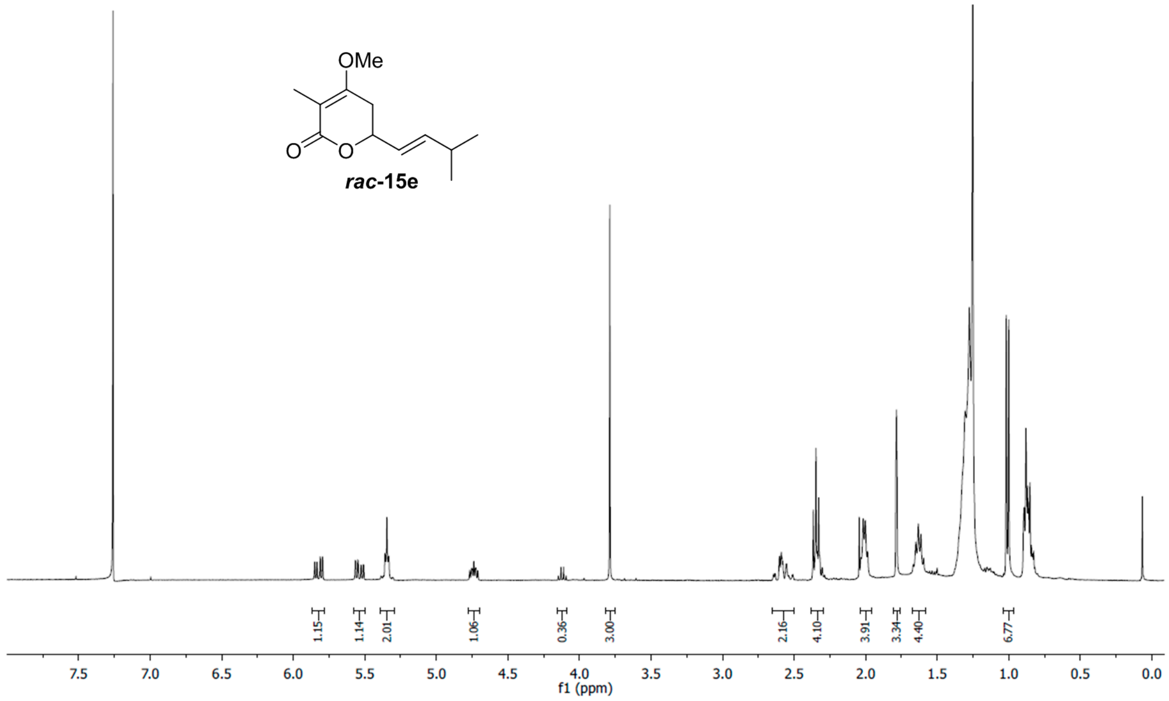

Figure S45. ^{1}H-NMR spectrum of the product from the JerF conversion assay with compound ***rac*-14e** ($CDCl_3$, 400 MHz). Purification was conducted by column chromatography on silica gel with PE:EtOAc mixtures as eluant.

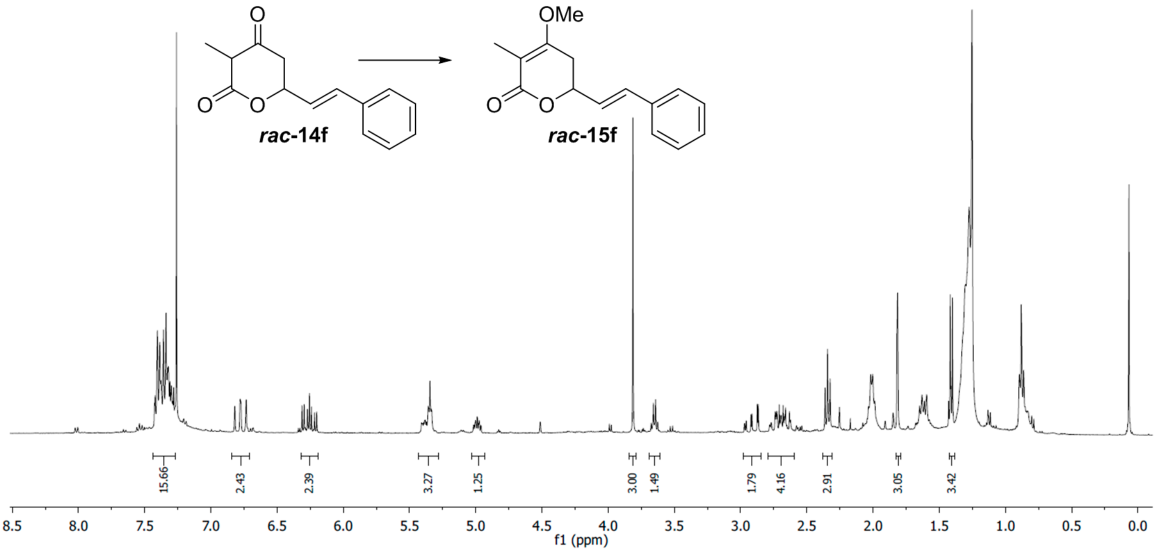

Figure S46. ^{1}H-NMR spectrum of the JerF conversion assay with compound ***rac*-14f** ($CDCl_3$, 400 MHz).

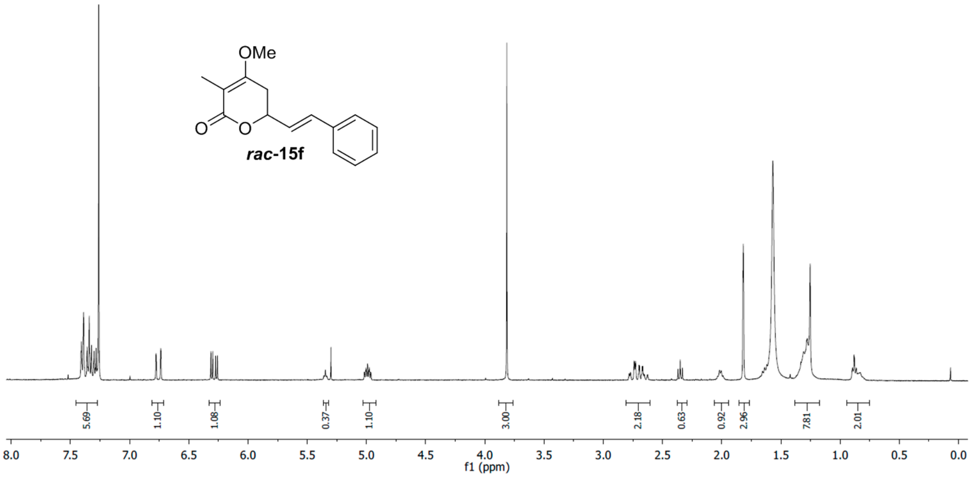

Figure S47. ^{1}H-NMR spectrum of the product from the JerF conversion assay with compound ***rac*-14f** ($CDCl_3$, 400 MHz). Purification was conducted by column chromatography on silica gel with PE:EtOAc mixtures as eluant.

4. HPLC-MS Analysis of the Enzymatic Assays with JerF

4.1. Establishment of assay conditions an reference experiments

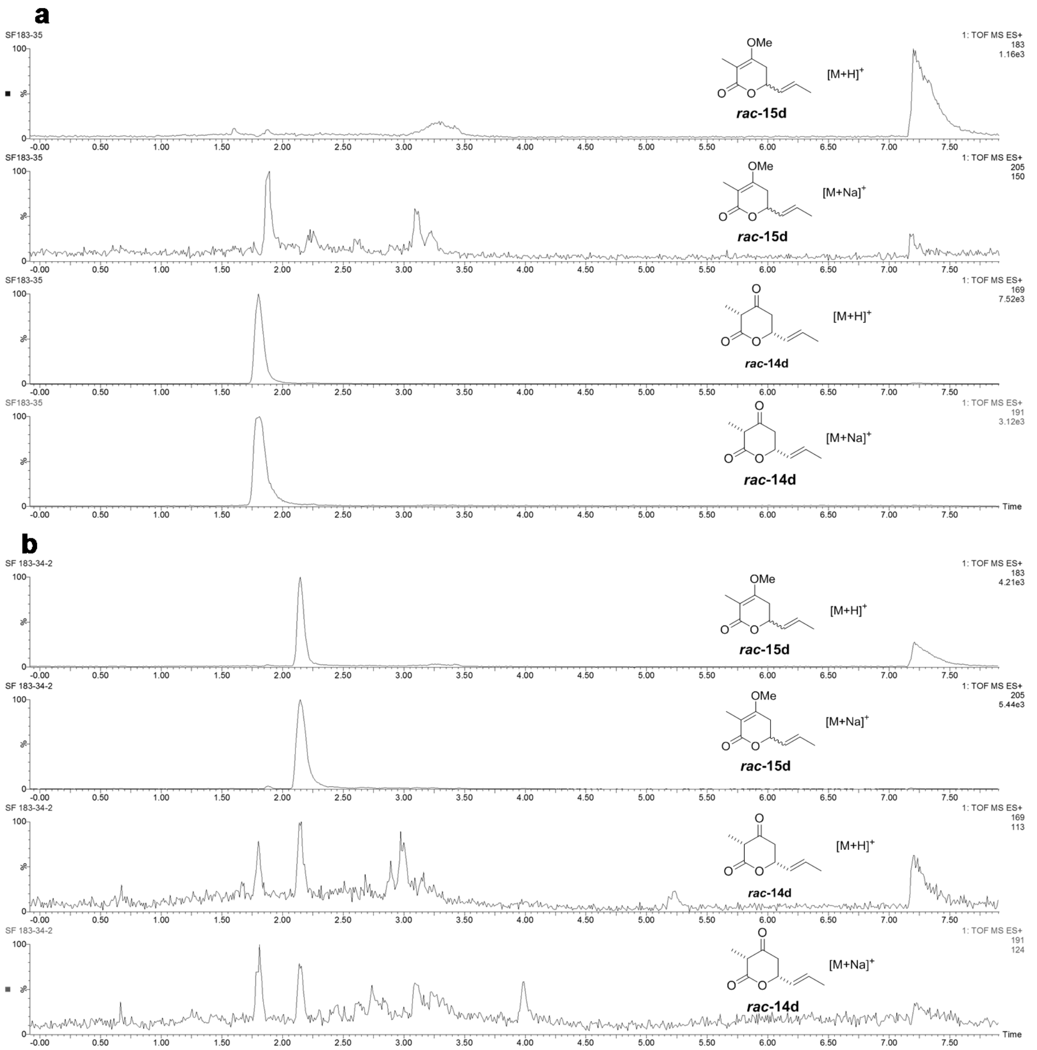

Figure S48. Unprocessed HPLC-MS chromatograms (ES^{+}) of Figure 1a (**a**) synthetic ***rac*-14d**; (**b**) synthetic ***rac*-15d**.

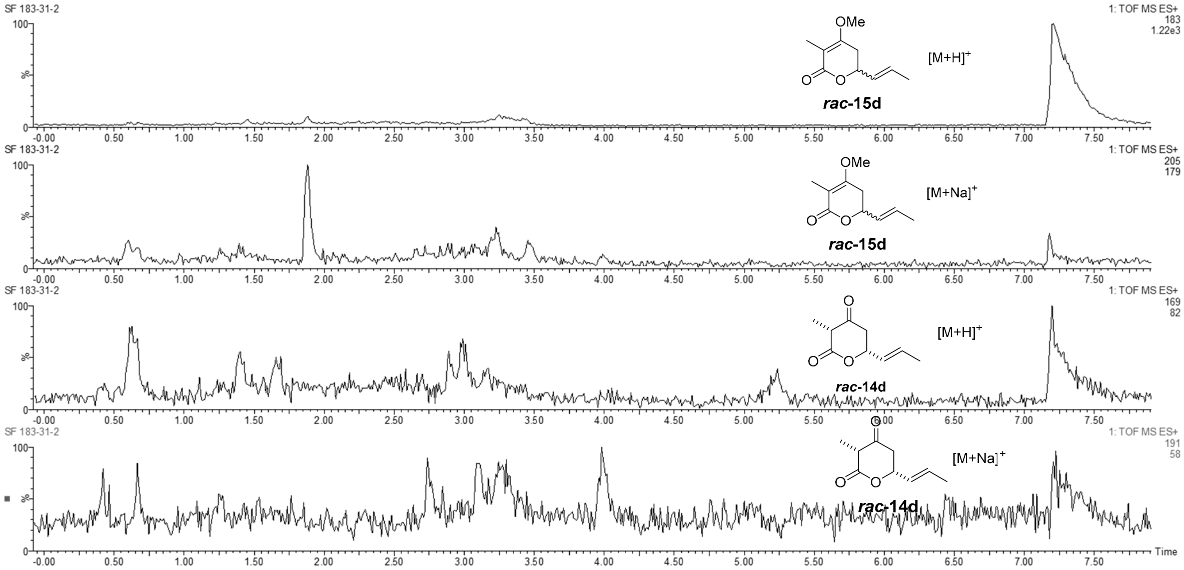

Figure S49. Unprocessed HPLC-MS chromatograms (ES+) of Figure 1b: incubation of JerF and SAM-tosylate.

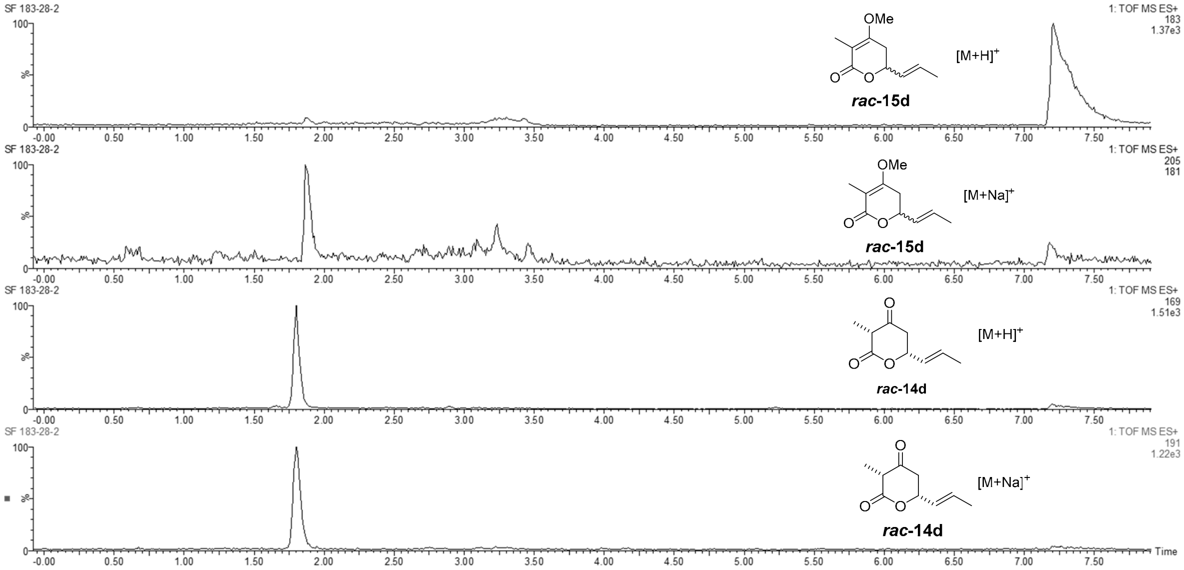

Figure S50. Unprocessed HPLC-MS chromatograms (ES+) of Figure 1b: incubation of ***rac*-14d** and SAM-tosylate.

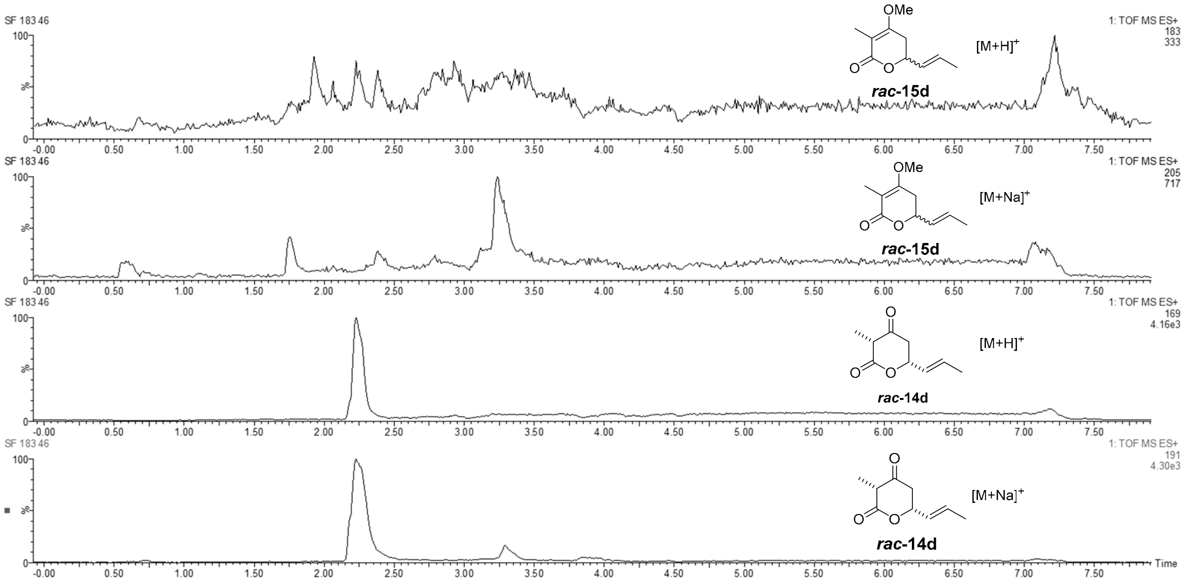

Figure S51. Unprocessed HPLC-MS chromatograms (ES+) of Figure 1b: incubation of denaturated JerF, ***rac*-14d** and SAM-tosylate.

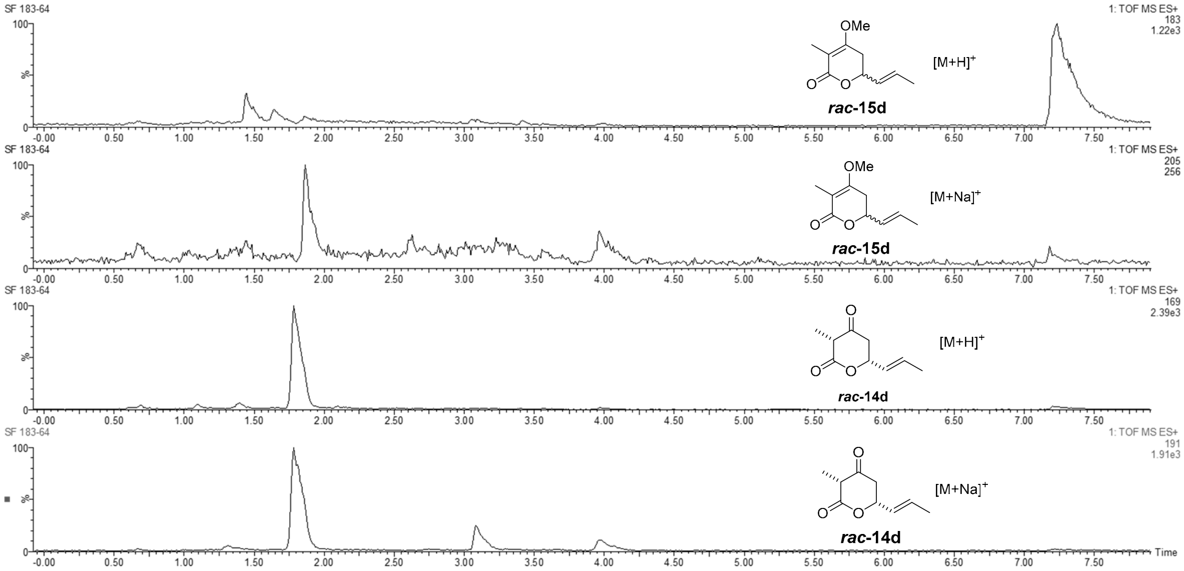

Figure S52. Unprocessed HPLC-MS chromatograms (ES+) of Figure 1b: incubation of *pCold* expression, ***rac*-14d** and SAM-tosylate.

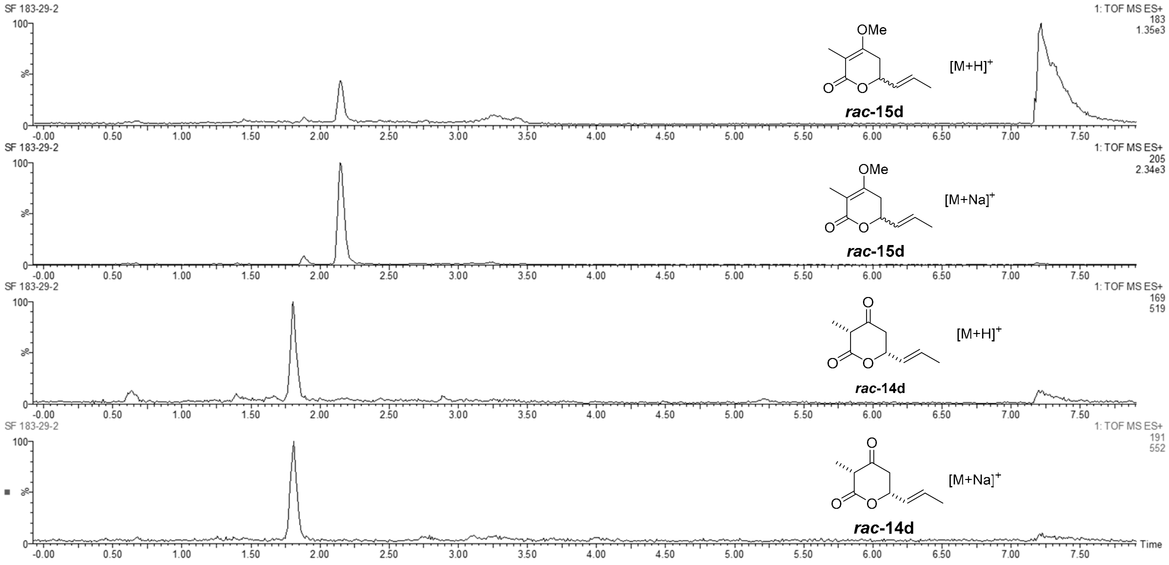

Figure S53. Unprocessed HPLC-MS chromatograms (ES+) of Figure 1b: incubation of JerF, ***rac*-14d** and SAM-tosylate.

4.2. Experiments at pH 8.8

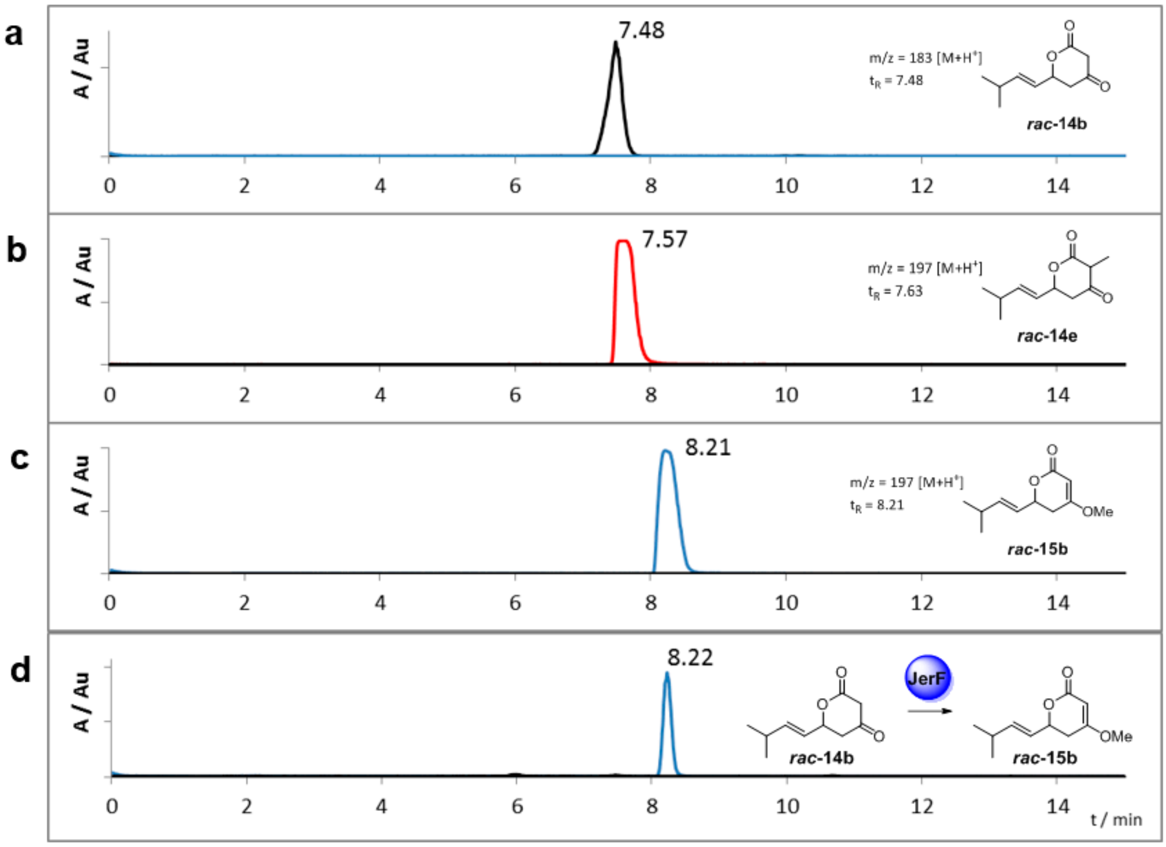

Figure S54. HPLC-MS analysis of the conversion assay of JerF with substrate ***rac*-14b** at pH 8.8. (**a**) synthetic ***rac*-14b**, mass traces for M = 183 (black) and M = 197 (blue); (**b**) synthetic ***rac*-14e**, mass traces for M = 183 (black) and M = 197 (red); (**c**) synthetic ***rac*-15b**, mass traces for M = 183 (black) and M = 197 (blue); (**d**) conversion experiment of JerF with ***rac*-14b**, mass traces for M = 183 (black) and M = 197 (blue); x-axis: retention time, y-axis: relative intensity.

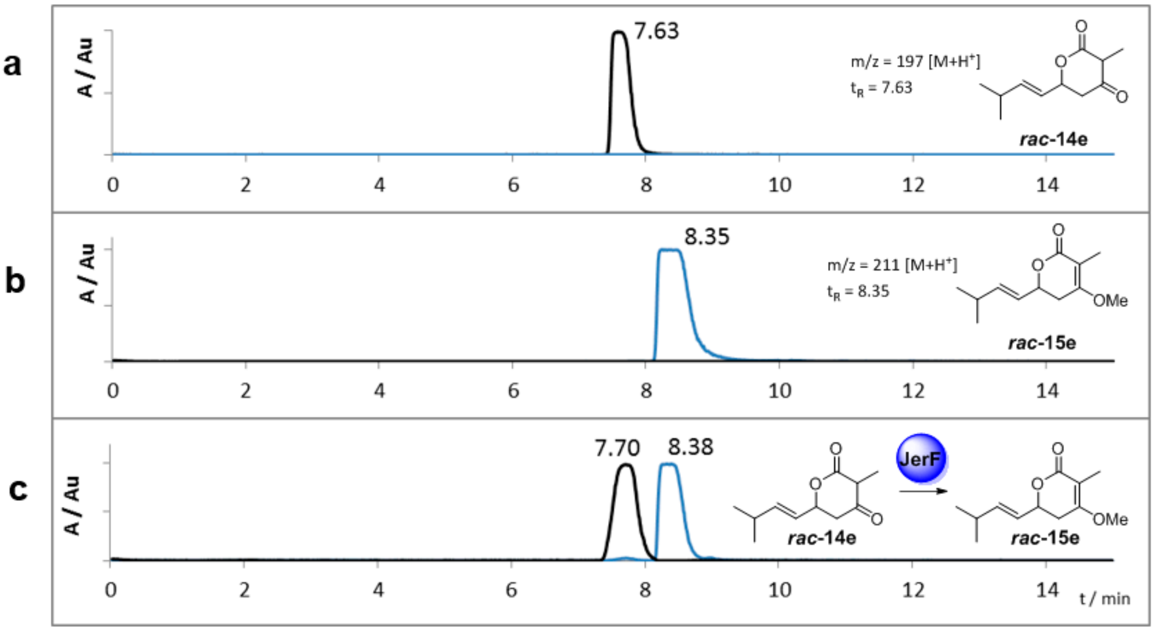

Figure S55. HPLC-MS analysis of the conversion assay of JerF with substrate ***rac*-14e** at pH 8.8. (**a**) synthetic ***rac*-14e**, mass traces for M = 197 (black) and M = 211 (blue); (**b**) synthetic ***rac*-15e**, mass traces for M = 197 (black) and M = 211 (blue); (**c**) conversion experiment of JerF with ***rac*-14e**, mass traces for M = 197 (black) and M = 211 (blue); x-axis: retention time, y-axis: relative intensity.

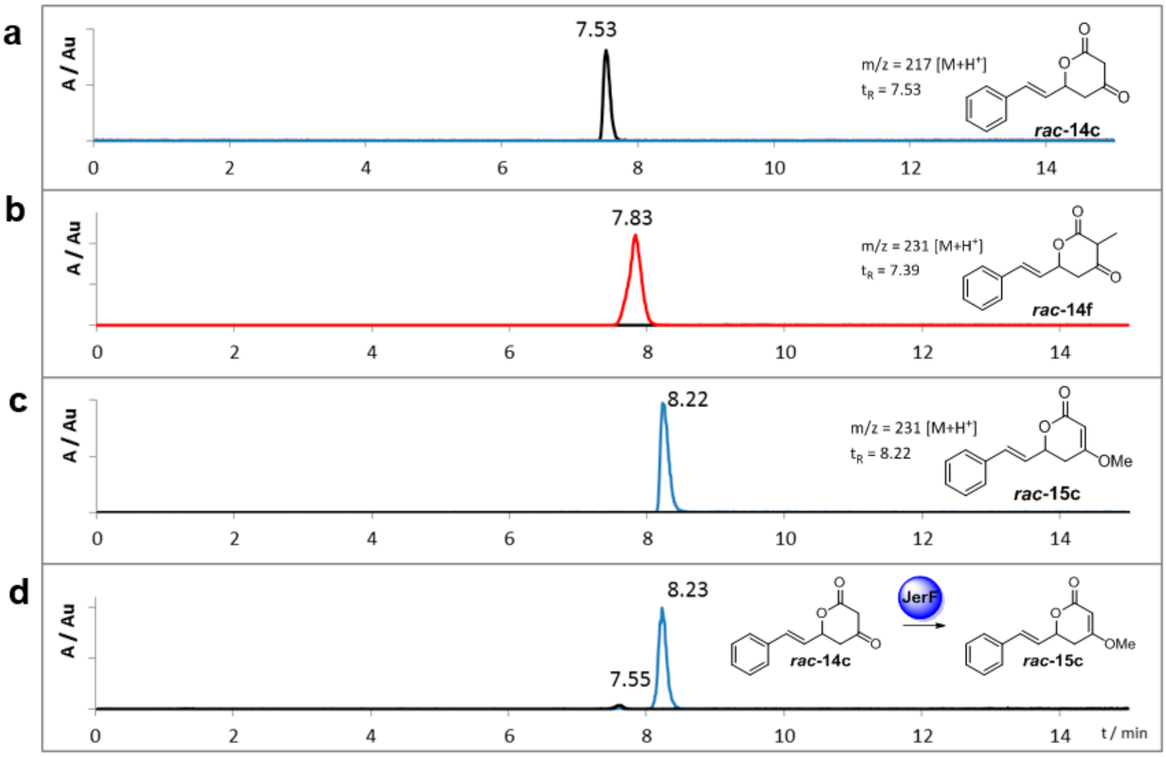

Figure S56. HPLC-MS analysis of the conversion assay of JerF with substrate ***rac*-14c** at pH 8.8. (**a**) synthetic ***rac*-14c**, mass traces for M = 217 (black) and M = 231 (blue); (**b**) synthetic ***rac*-14f**, mass traces for M = 217 (black) and M = 231 (red); (**c**) synthetic ***rac*-15c**, mass traces for M = 217 (black) and M = 231 (blue); (**d**) conversion experiment of JerF with ***rac*-14c**, mass traces for M = 217 (black) and M = 231 (blue); x-axis: retention time, y-axis: relative intensity.

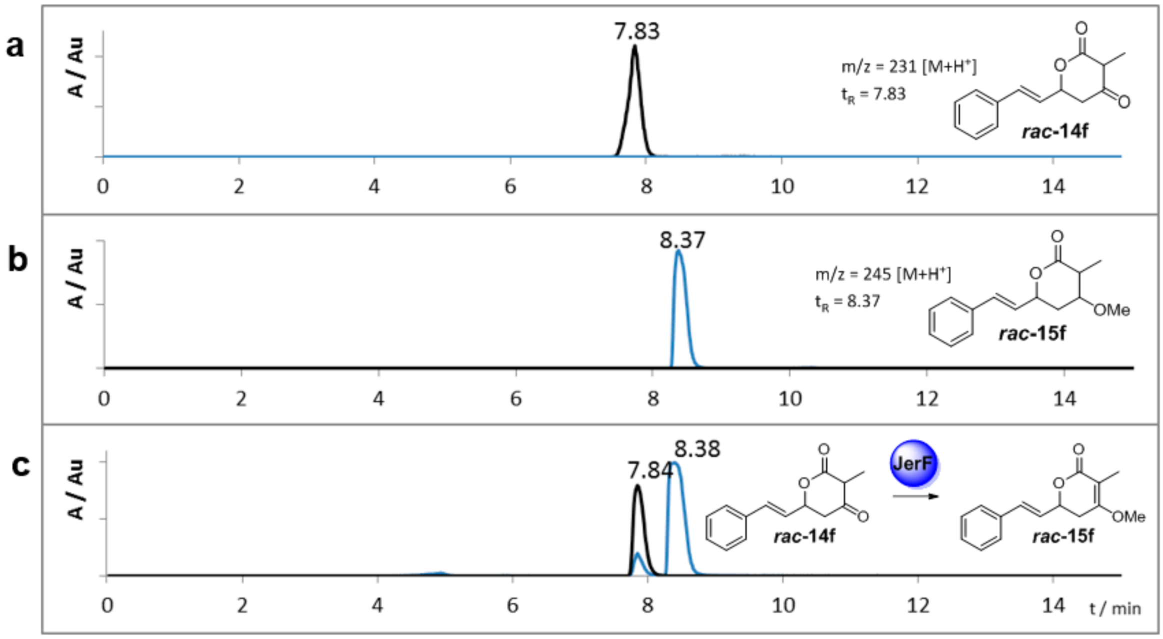

Figure S57. HPLC-MS analysis of the conversion assay of JerF with substrate ***rac*-14f** at pH 8.8. (**a**) synthetic ***rac*-14f**, mass traces for M = 231 (black) and M = 245 (blue); (**b**) synthetic ***rac*-15f**, mass traces for M = 231 (black) and M = 245 (blue); (**c**) conversion experiment of JerF with ***rac*-14f**, mass traces for M = 231 (black) and M = 245 (blue); x-axis: retention time, y-axis: relative intensity.

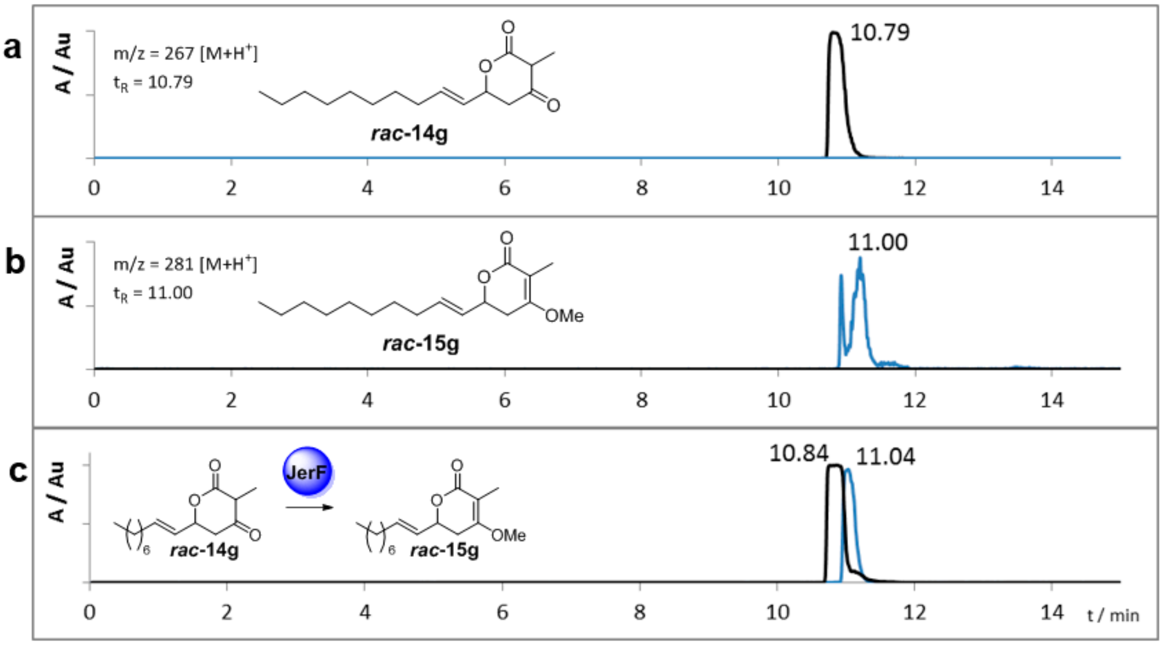

Figure S58. HPLC-MS analysis of the conversion assay of JerF with substrate ***rac*-14g** at pH 8.8. (**a**) synthetic ***rac*-14g**, mass traces for M = 267 (black) and M = 281 (blue); (**b**) synthetic ***rac*-15g**, mass traces for M = 267 (black) and M = 281 (blue); (**c**) conversion experiment of JerF with ***rac*-14g**, mass traces for M = 267 (black) and M = 281 (blue); x-axis: retention time, y-axis: relative intensity.

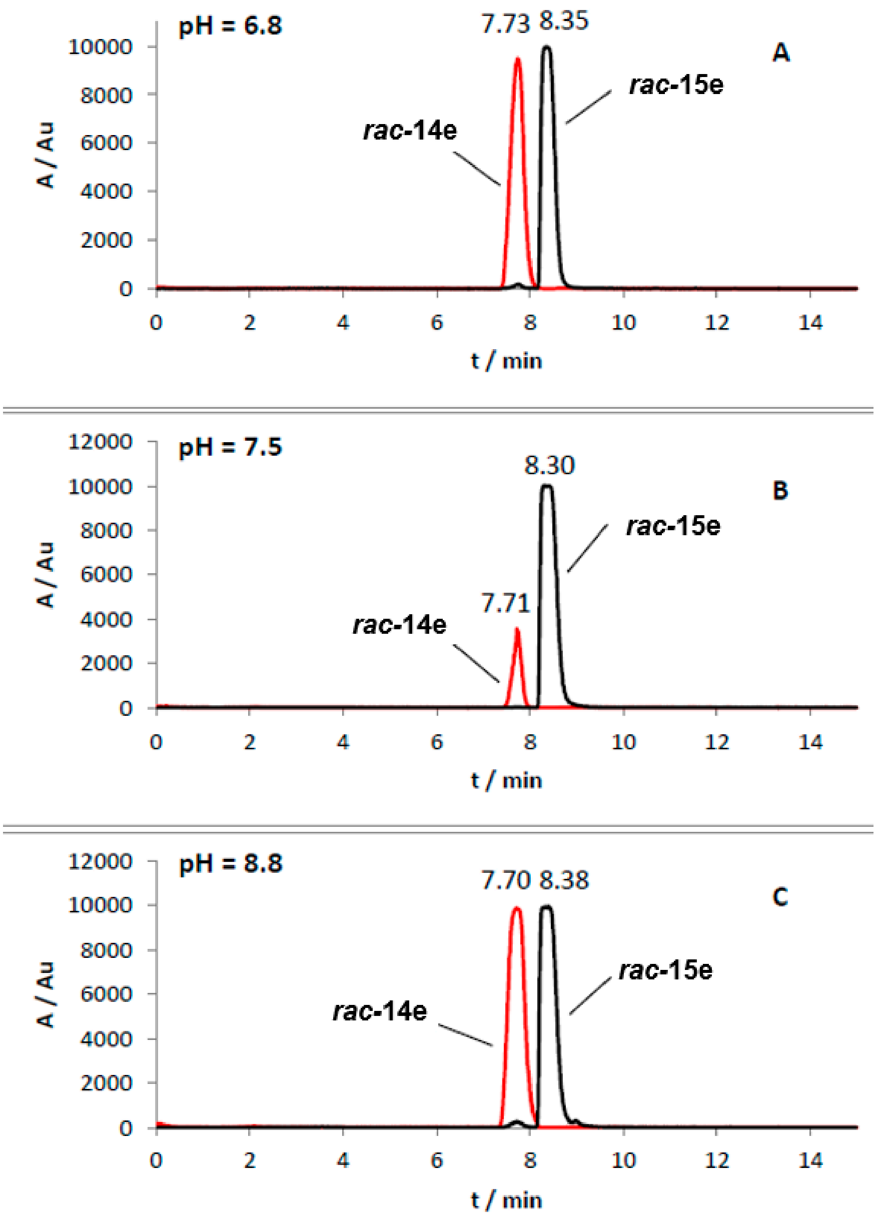

Figure S59. HPLC-MS analysis of the conversion assay of JerF with substrate ***rac*-14e** at various pH values. (**a**) conversion experiment at pH 6.8, mass traces for M = 197 (red) and M = 211 (black); (**b**) conversion experiment at pH 7.5, mass traces for M = 197 (red) and M = 211 (black); (**c**) conversion experiment at pH 8.8, mass traces for M = 197 (red) and M = 211 (black); x-axis: retention time, y-axis: relative intensity

4.3. Experiments at pH 7.5

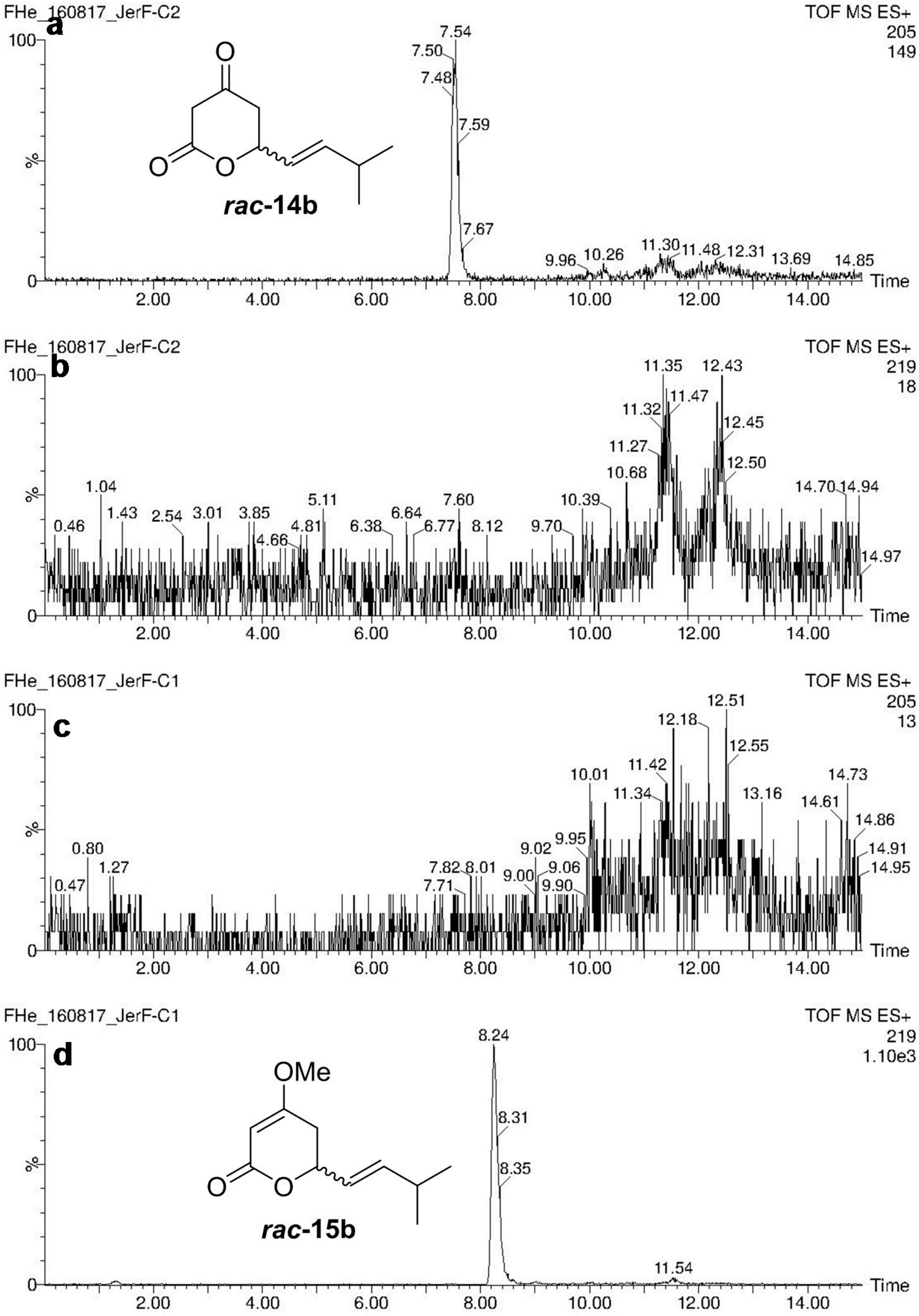

Figure S60. HPLC-MS analysis of the conversion assay of JerF with substrate ***rac*-14b** at pH 7.5. (**a**) synthetic ***rac*-14b**, mass trace for M = 205; (**b**) synthetic ***rac*-14b**, mass trace for M = 219; (**c**) conversion of JerF with ***rac*-14b**, mass trace for M = 205; (**d**) conversion of JerF with ***rac*-14b**, mass trace for M = 219; (***rac*-14b**) = 205 [M + Na]$^+$, (***rac*-15b**) = 219 [M + Na]$^+$, x-axis: retention time, y-axis: relative intensity.

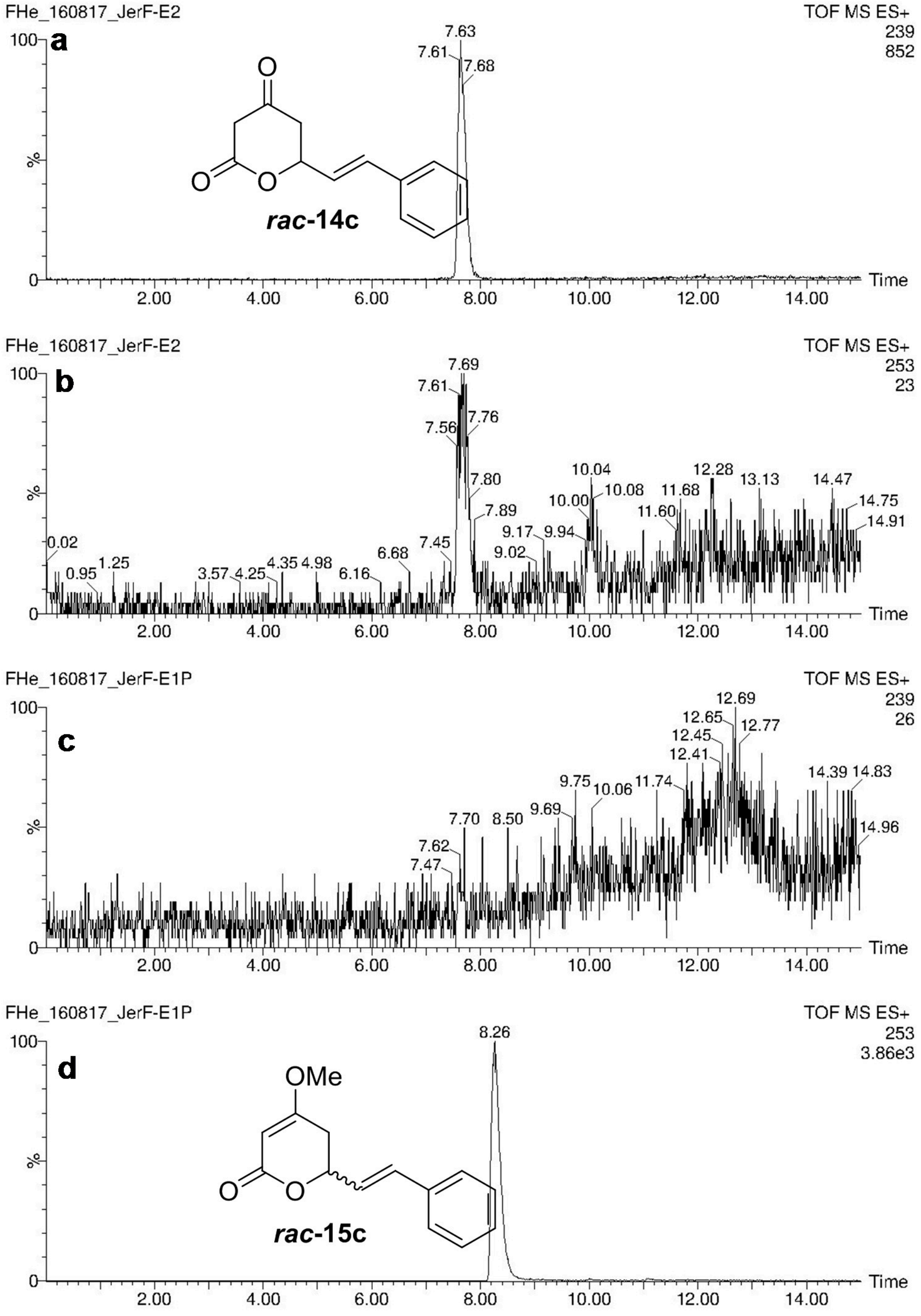

Figure S61. HPLC-MS analysis of the conversion assay of JerF with substrate ***rac*-14c** at pH 7.5. (**a**) synthetic ***rac*-14c**, mass trace for M = 239; (**b**) synthetic ***rac*-14c**, mass trace for M = 253; (**c**) conversion of JerF with ***rac*-14c**, mass trace for M = 239; (**d**) conversion of JerF with ***rac*-14c**, mass trace for M = 253; (***rac*-14c**) = 239 $[M + Na]^+$, (***rac*-15c**) = 253 $[M + Na]^+$, x-axis: retention time, y-axis: relative intensity.

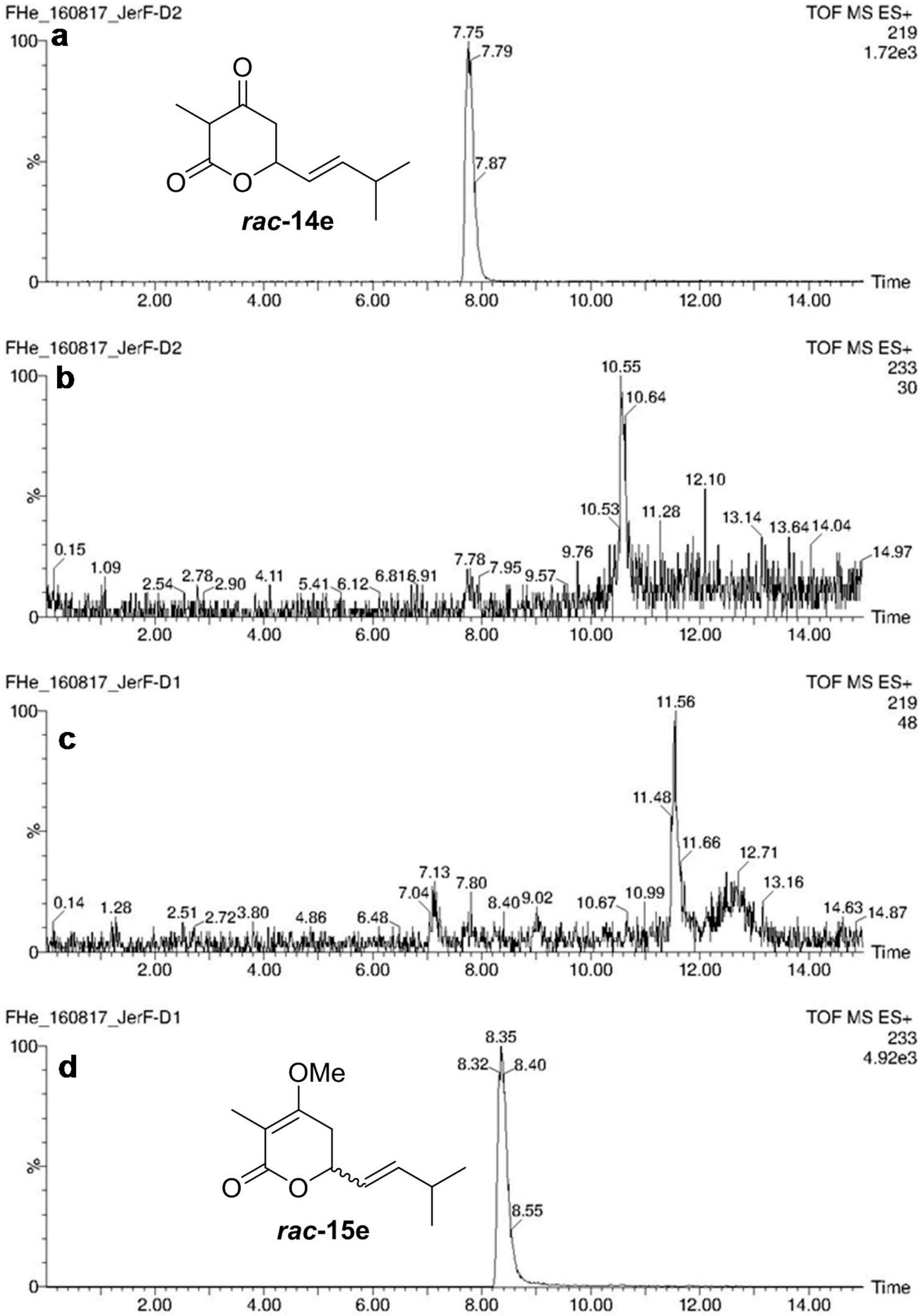

Figure S62. HPLC-MS analysis of the conversion assay of JerF with substrate ***rac*-14e** at pH 7.5. (**a**) synthetic ***rac*-14e**, mass trace for M = 219; (**b**) synthetic ***rac*-14e**, mass trace for M = 233; (**c**) conversion of JerF with ***rac*-14e**, mass trace for M = 219; (**d**) conversion of JerF with ***rac*-14e**, mass trace for M = 233; (***rac*-14e**) = 219 [M + Na]$^+$, (***rac*-15e**) = 233 [M + Na]$^+$, x-axis: retention time, y-axis: relative intensity.

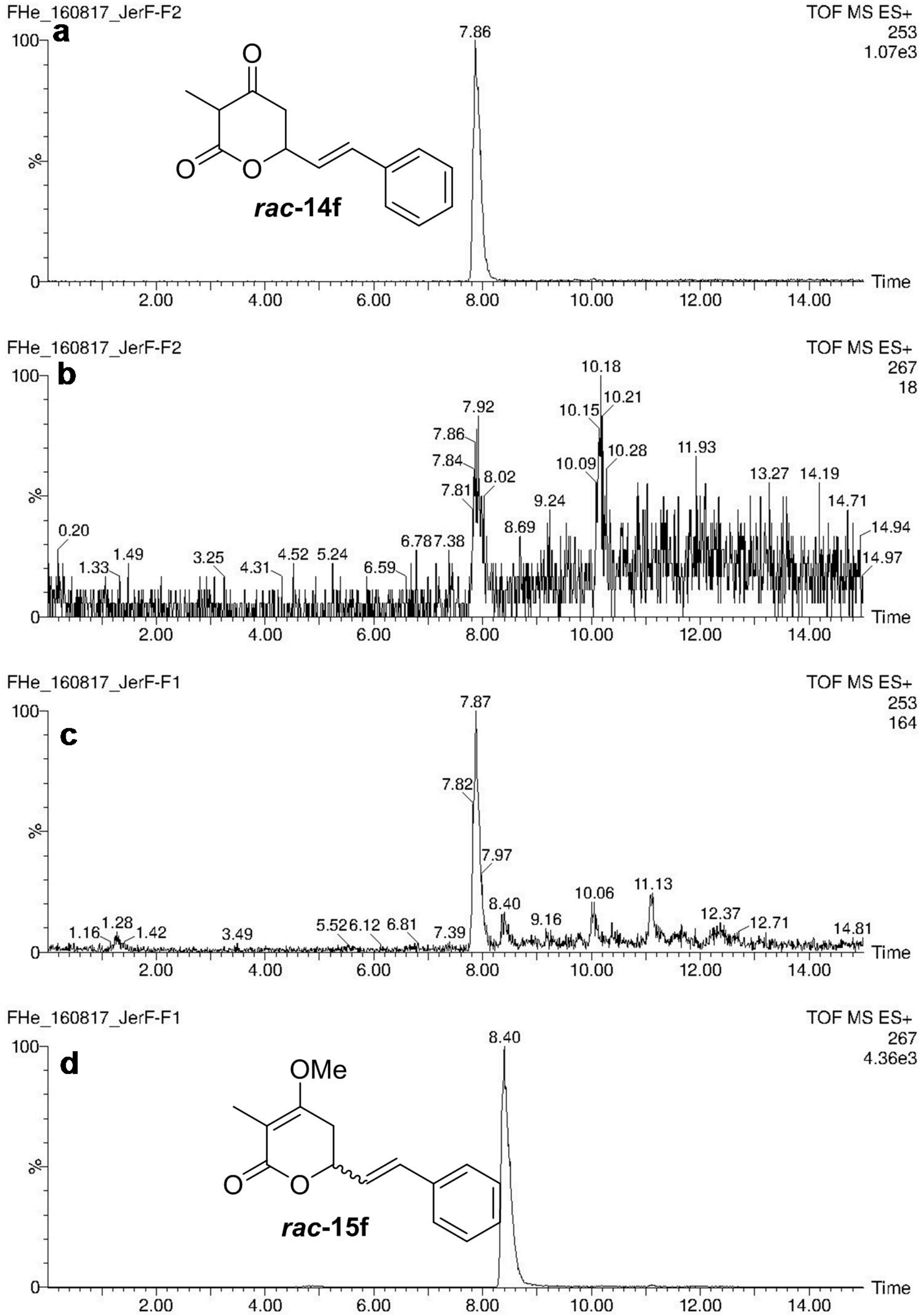

Figure S63. HPLC-MS analysis of the conversion assay of JerF with substrate ***rac*-14f** at pH 7.5. (**a**) synthetic ***rac*-14f**, mass trace for M = 253; (**b**) synthetic ***rac*-14f**, mass trace for M = 267; (**c**) conversion of JerF with ***rac*-14f**, mass trace for M = 253; (**d**) conversion of JerF with ***rac*-14f**, mass trace for M = 267; (***rac*-14f**) = 253 $[M + Na]^+$, (***rac*-15f**) = 267 $[M + Na]^+$, x-axis: retention time, y-axis: relative intensity.

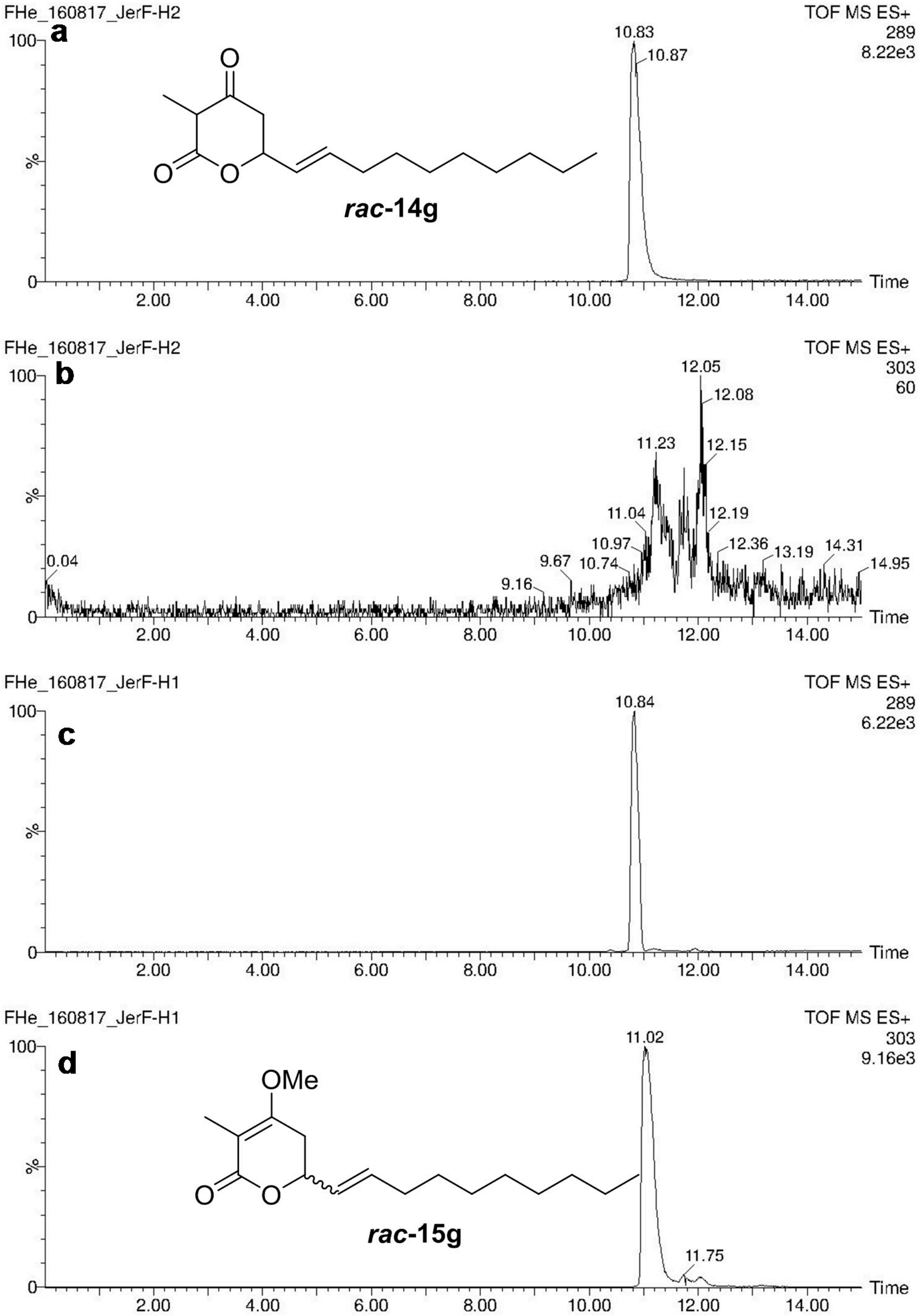

Figure S64. HPLC-MS analysis of the conversion assay of JerF with substrate ***rac*-14g** at pH 7.5. (**a**) synthetic ***rac*-14g**, mass trace for M = 289; (**b**) synthetic ***rac*-14g**, mass trace for M = 303; (**c**) conversion of JerF with ***rac*-14g**, mass trace for M = 289; (**d**) conversion of JerF with ***rac*-14g**, mass trace for M = 303; (***rac*-14g**) = 289 $[M + Na]^+$, (***rac*-15g**) = 303 $[M + Na]^+$, x-axis: retention time, y-axis: relative intensity.

5. Chiral HPLC Analysis

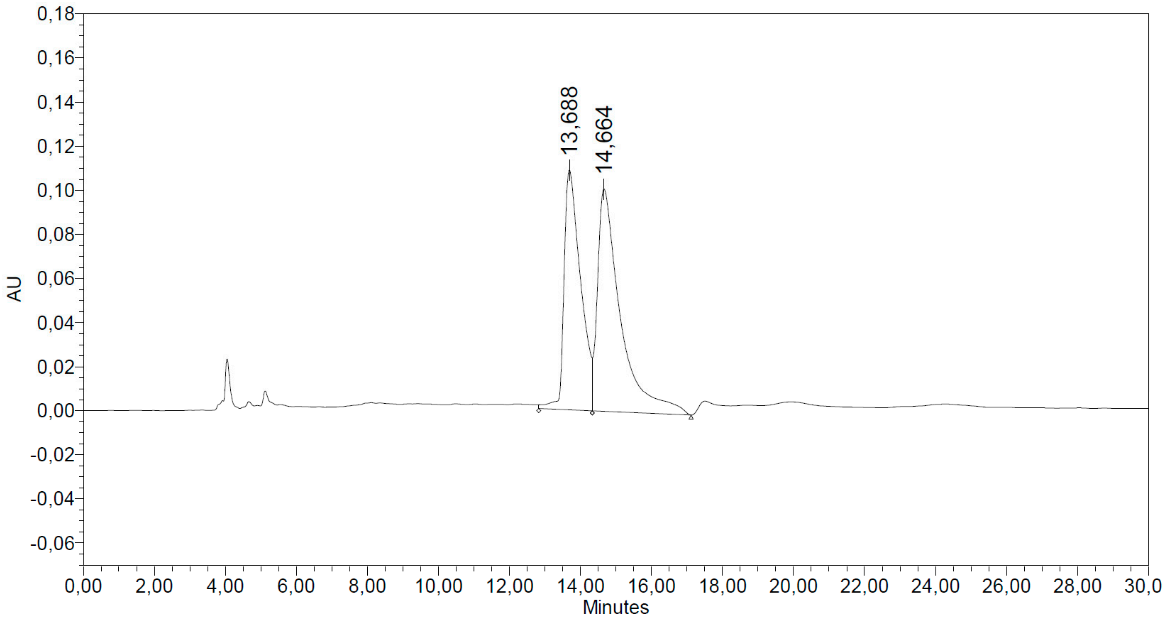

	Ret. Time	Start (min)	End (min)	Height	% Height	Area	% Area
1	13,69	10,29	17,12	108871	51,88	3546240	43,75
2	14,66	10,29	17,12	100999	48,12	4558553	56,25

(**a**)

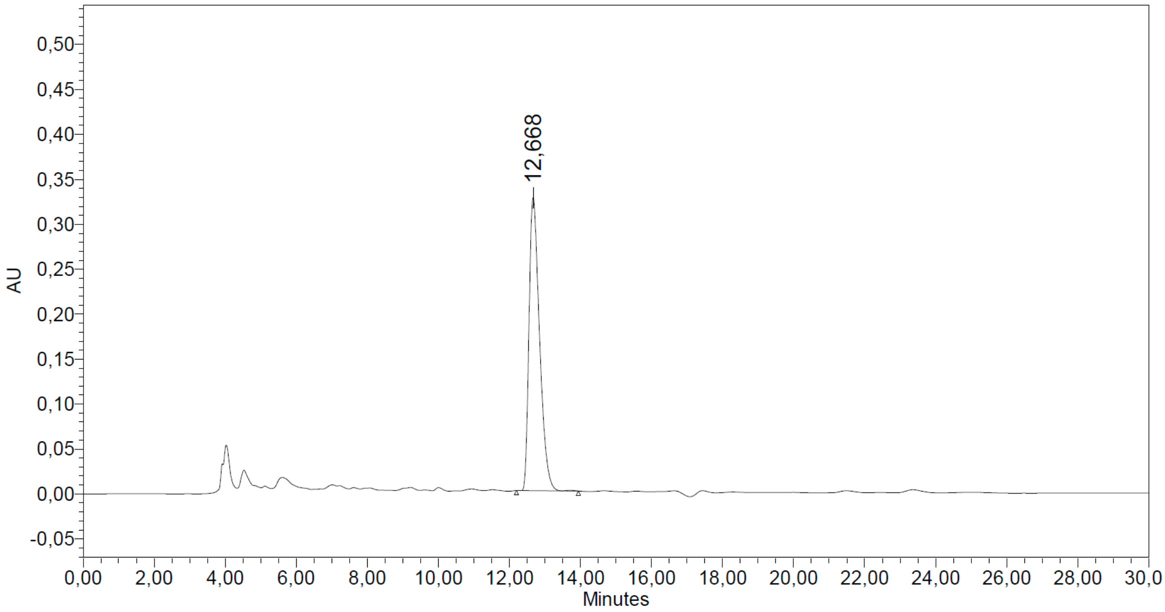

	Ret. Time	Start (min)	End (min)	Height	% Height	Area	% Area
1	12,67	12,20	13,94	326049	100,00	6721569	100,00

(**b**)

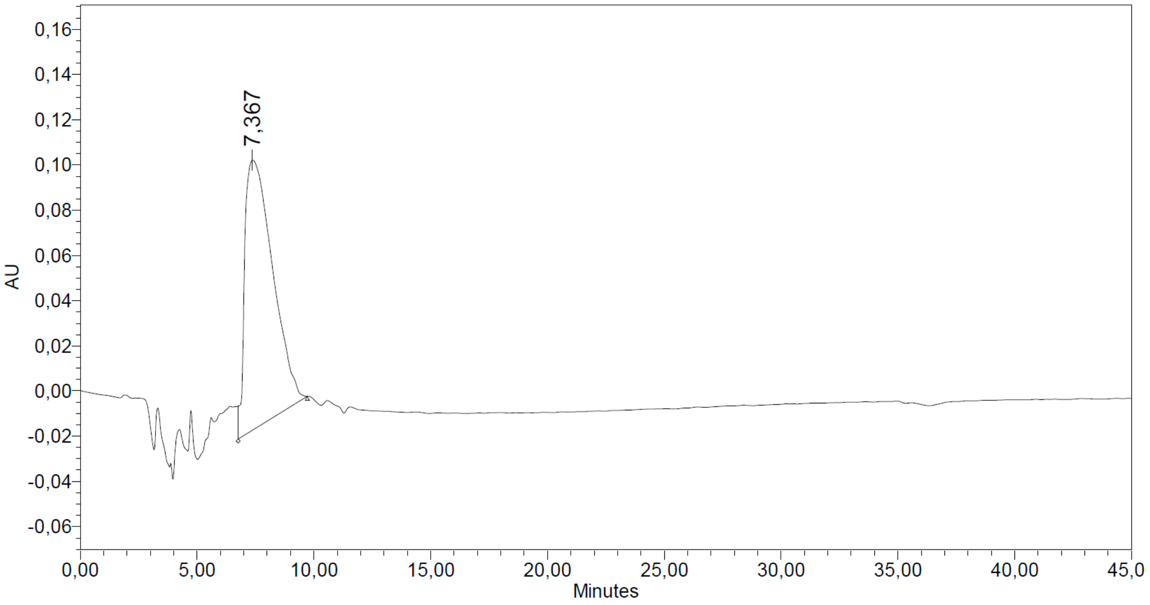

	Ret. Time	Start (min)	End (min)	Height	% Height	Area	% Area
1	7,37	3,97	9,72	119561	100,00	9879692	100,00

(**c**)

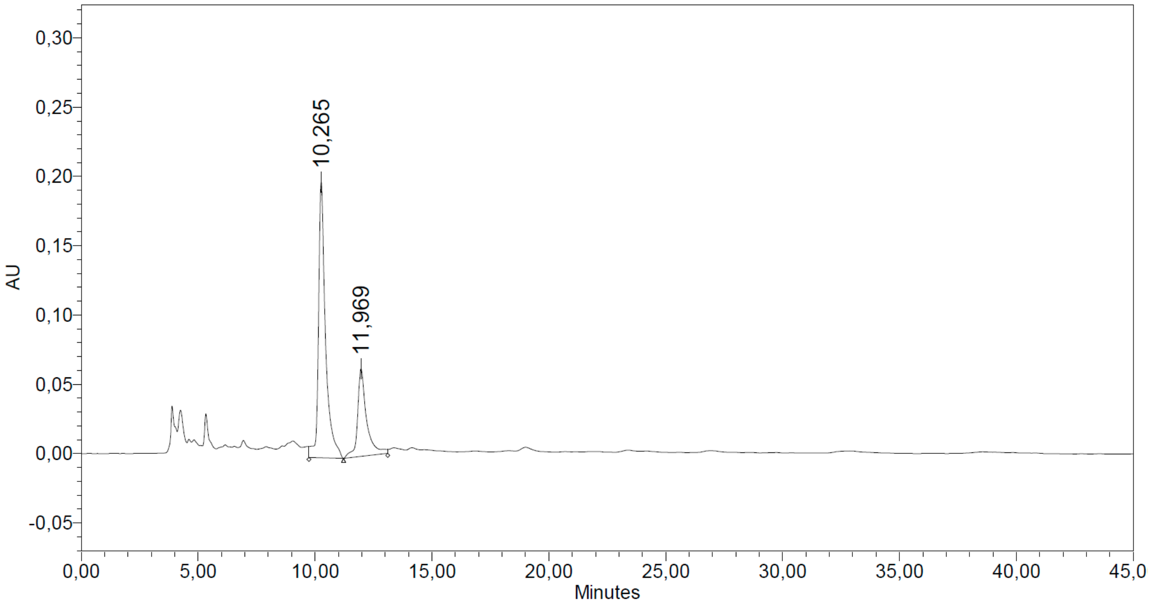

	Ret. Time	Start (min)	End (min)	Height	% Height	Area	% Area
1	10,26	3,53	11,22	199037	75,89	4168798	70,92
2	11,97	11,23	14,48	63249	24,11	1709424	29,08

(**d**)

Figure S65. Analysis of synthetic ***rac*-14e** and the product of the reaction between JerF and ***rac*-14e** on the analytical scale. As the stereoisomers present in ***rac*-14e** and the assay product could not be separated under identical conditions, those were individually adjusted (**a**/**b** and **c**/**d**). (**a**) Analysis of synthetic ***rac*-14e** by conditions 1. Only two peaks are visible as the *syn*-diastereomers strongly dominate over the *anti*-diastereomers (see above); (**b**) Analysis of the assay product by conditions 1 after column chromatography; (**c**) Analysis of synthetic ***rac*-14e** by conditions 2; (**d**) Analysis of the assay product by conditions 2 after column chromatography.

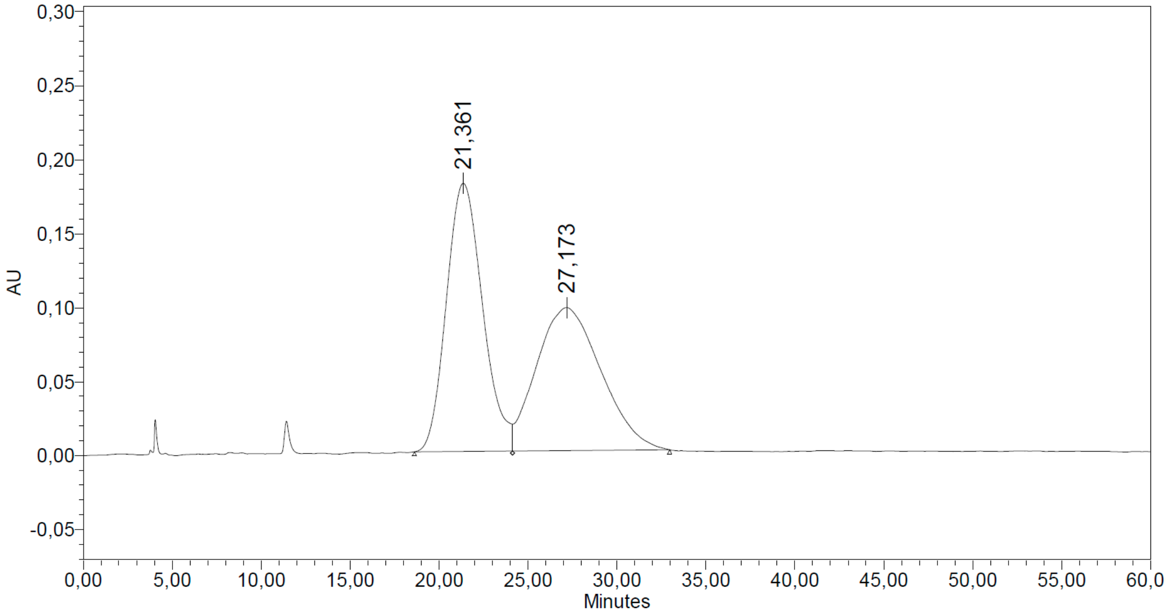

	Ret. Time	Start (min)	End (min)	Height	% Height	Area	% Area
1	21,36	18,62	32,97	181381	65,17	25807228	51,32
2	27,17	18,62	32,97	96942	34,83	24481611	48,68

(**a**)

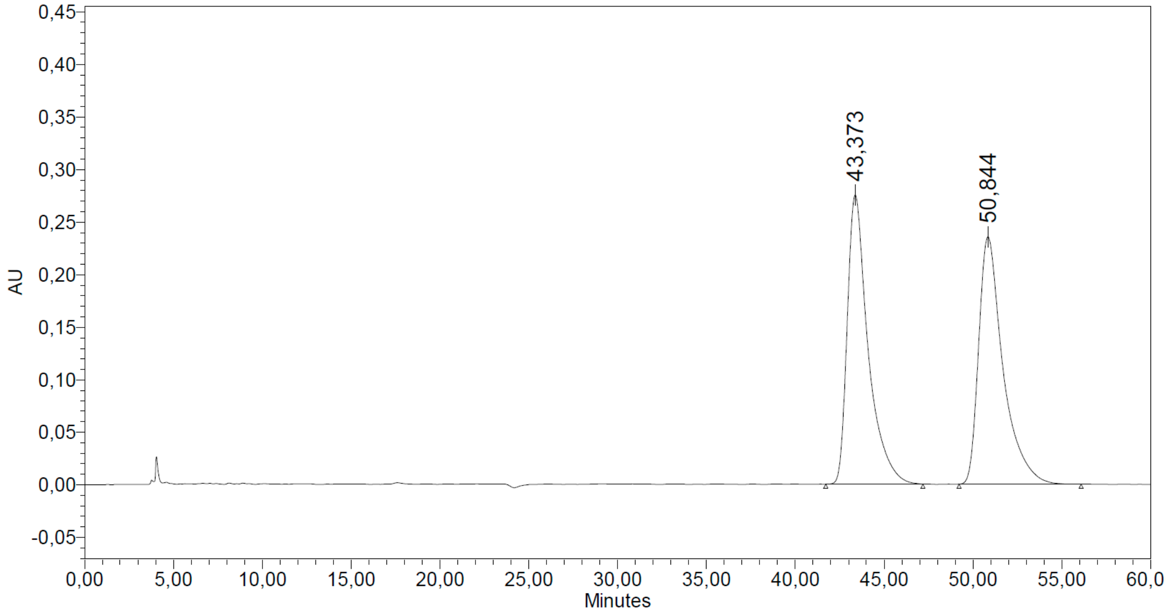

	Ret. Time	Start (min)	End (min)	Height	% Height	Area	% Area
1	43,37	41,72	47,18	275369	53,90	22437637	50,05
2	50,84	49,22	56,08	235480	46,10	22391423	49,95

(**b**)

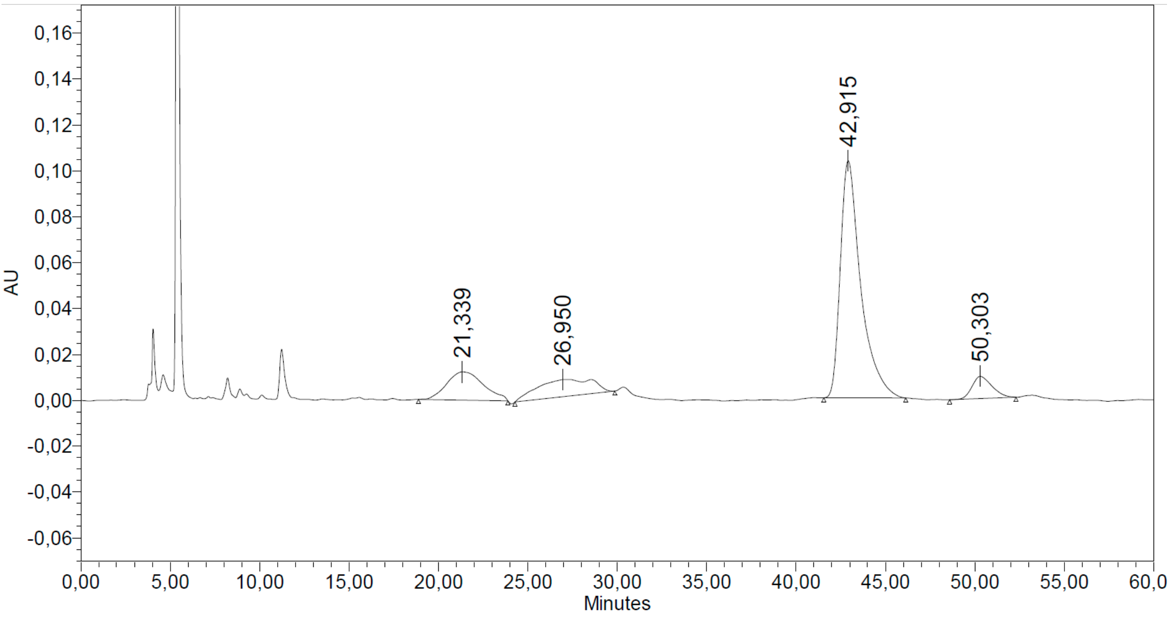

	Ret. Time	Start (min)	End (min)	Height	% Height	Area	% Area
1	21,34	18,88	23,88	12262	9,24	1760322	14,29
2	26,95	24,28	29,87	7460	5,62	1595685	12,96
3	42,92	41,55	46,13	103238	77,83	8207553	66,65
4	50,30	48,58	52,30	9686	7,30	751279	6,10

(**c**)

Figure S66. Analysis of synthetic ***rac*-14c** and the product of the reaction between JerF and ***rac*-14c** on the analytical scale. (**a**) Analysis of synthetic ***rac*-14c** by conditions 3; (**b**) Analysis of synthetic ***rac*-15c** by conditions 3; (**c**) Analysis of the crude assay product by conditions 3.

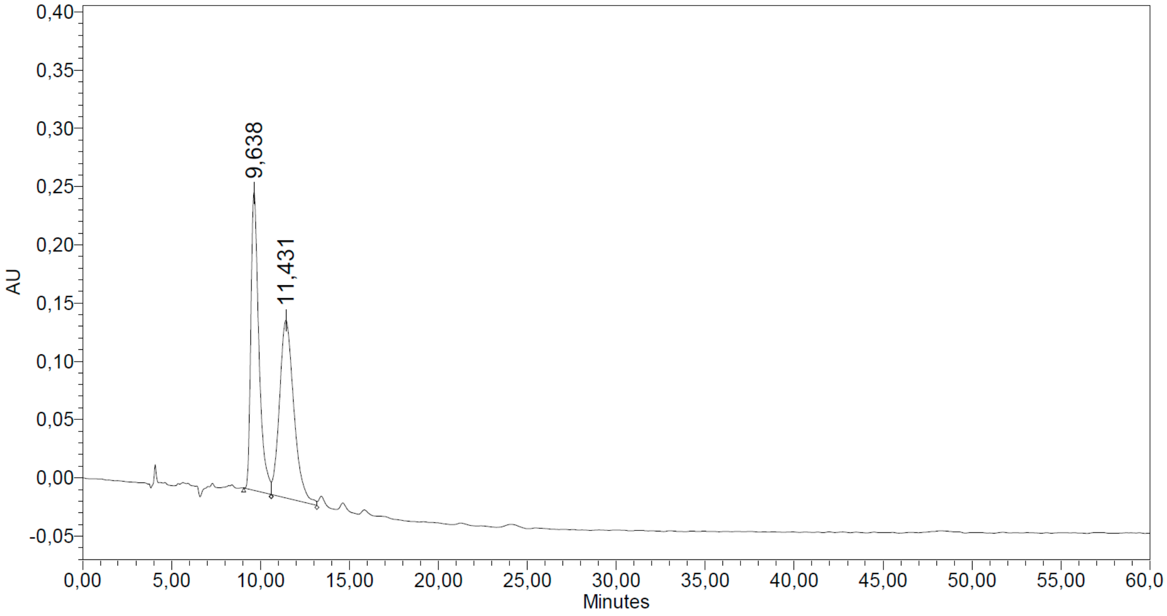

	Ret. Time	Start (min)	End (min)	Height	% Height	Area	% Area
1	9,64	9,06	14,02	255767	62,64	8042470	48,43
2	11,43	9,06	14,02	152523	37,36	8564463	51,57

(**a**)

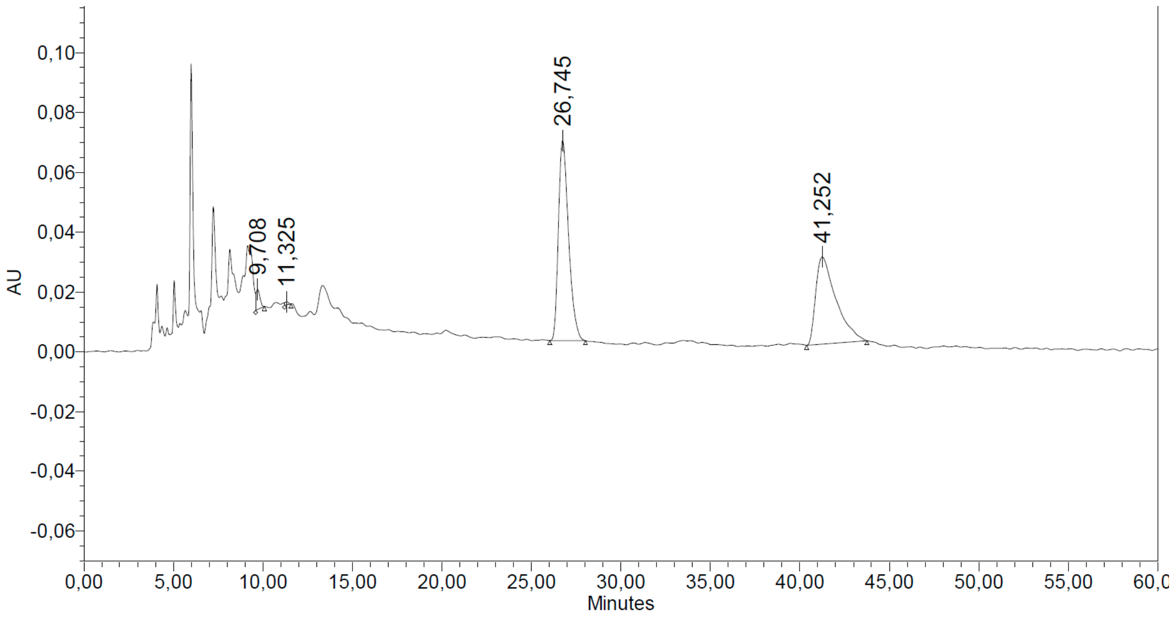

	Ret. Time	Start (min)	End (min)	Height	% Height	Area	% Area
1	9,71	6,73	10,08	6722	6,48	102771	2,03
2	11,33	10,41	11,57	996	0,96	13984	0,28
3	26,75	26,03	28,02	66890	64,46	2638492	52,16
4	41,25	40,38	43,75	29156	28,10	2302973	45,53

(**b**)

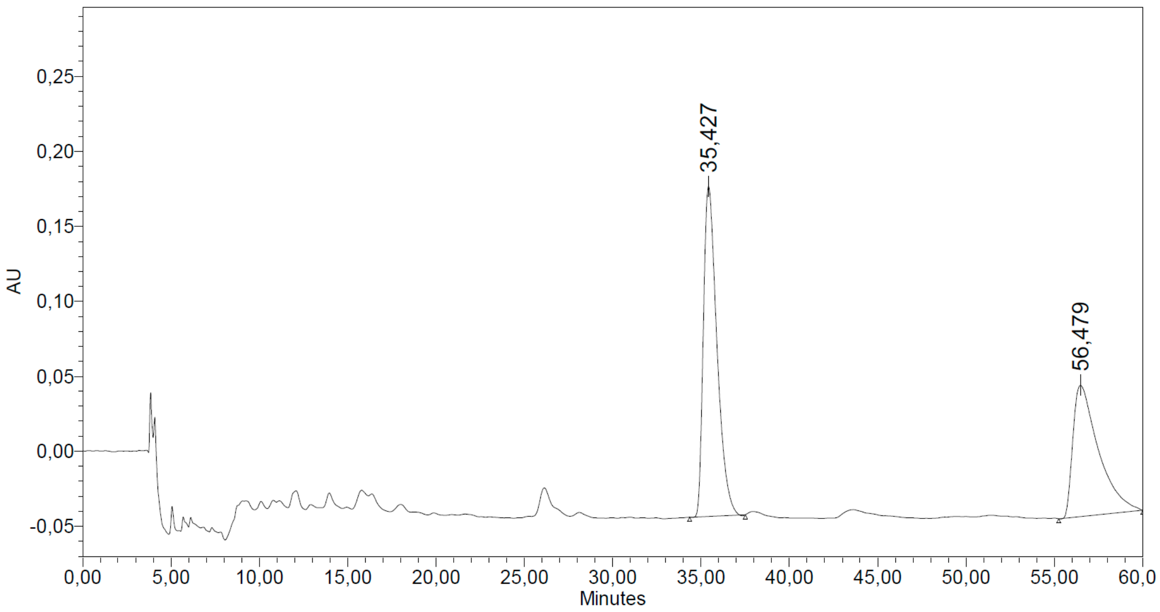

	Ret. Time	Start (min)	End (min)	Height	% Height	Area	% Area
1	35,43	34,36	37,51	220167	71,51	11756015	57,15
2	56,48	55,24	60,00	87713	28,49	8814513	42,85

(**c**)

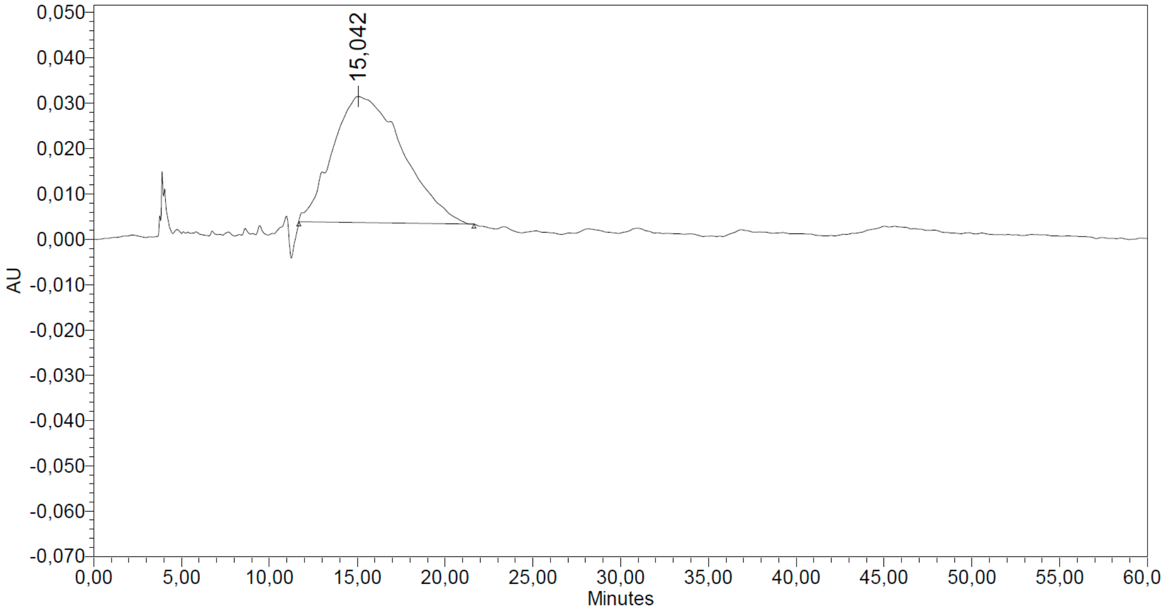

	Ret. Time	Start (min)	End (min)	Height	% Height	Area	% Area
1	15,04	11,68	21,65	27777	100,00	7796933	100,00

(**d**)

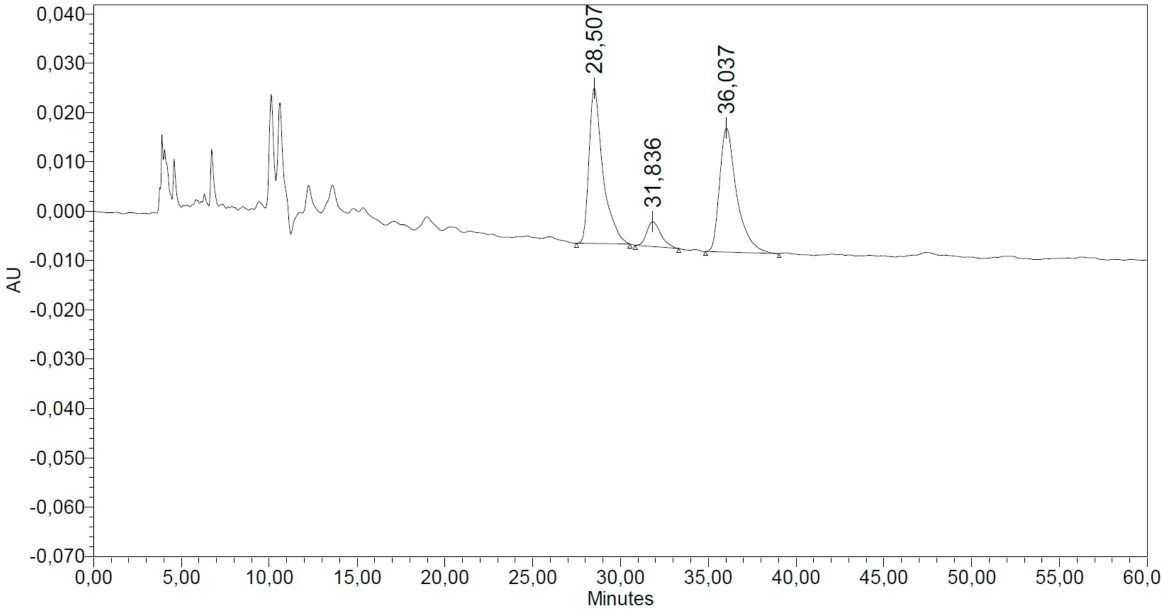

	Ret. Time	Start (min)	End (min)	Height	% Height	Area	% Area
1	28,51	27,50	30,53	31496	51,09	1737250	46,65
2	31,84	30,83	33,32	5018	8,14	281318	7,55
3	36,04	34,84	39,03	25131	40,77	1705795	45,80

(**e**)

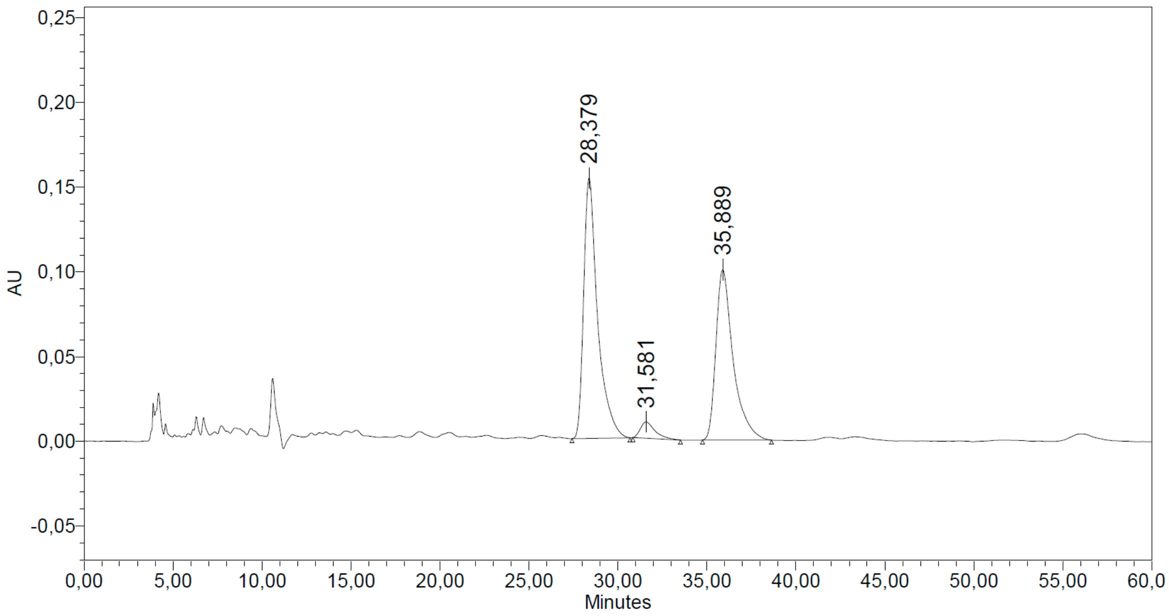

	Ret. Time	Start (min)	End (min)	Height	% Height	Area	% Area
1	28,38	27,42	30,68	153892	58,24	8404520	53,35
2	31,58	30,83	33,52	9625	3,64	552040	3,50
3	35,89	34,76	38,63	100724	38,12	6797000	43,15

(**f**)

Figure S67. Analysis of synthetic ***rac*-14f** and the product of the reaction between JerF and ***rac*-14f** on the analytical scale. As the stereoisomers present in ***rac*-14f** and ***rac*-15f** could not be separated under identical conditions, those were individually adjusted (**a**–**f**). (**a**) Analysis of synthetic ***rac*-14f** by conditions 4. Only two peaks are visible as the *syn*-diastereomers strongly dominate over the *anti*-diastereomers (see above); (**b**) Analysis of synthetic ***rac*-15f** by conditions 4; (**c**) Analysis of the assay product by conditions 5 after column chromatography; (**d**) Analysis of synthetic ***rac*-14f** by conditions 6; (**e**) Analysis of synthetic ***rac*-15f** by conditions 6; (**f**) Analysis of the assay product by conditions 6 after column chromatography.
